# Bridge to neuroscience workshop: An effective educational tool to introduce principles of neuroscience to Hispanics students

**DOI:** 10.1371/journal.pone.0225116

**Published:** 2019-12-12

**Authors:** Alexandra Colón-Rodríguez, Chelsea T. Tiernan, Eileen S. Rodriguez-Tapia, William D. Atchison

**Affiliations:** 1 Comparative Medicine and Integrative Biology Program, College of Veterinary Medicine, Michigan State University, East Lansing, Michigan, United States of America; 2 Department of Pharmacology and Toxicology, College of Veterinary Medicine, Michigan State University, East Lansing, Michigan, United States of America; 3 Department of Translational Science & Molecular Medicine, College of Human Medicine, Michigan State University, Grand Rapids, Michigan, United States of America; 4 Neuroscience Program, College of Natural Science, Michigan State University, East Lansing, Michigan, United States of America; Charles P. Darby Children's Research Institute, 173 Ashley Avenue, Charleston, SC 29425, USA, UNITED STATES

## Abstract

Neuroscience as a discipline is rarely covered in educational institutions in Puerto Rico. In an effort to overcome this deficit we developed the Bridge to Neuroscience Workshop (BNW), a full-day hands-on workshop in neuroscience education. BNW was conceived as an auxiliary component of a parent recruitment program called Bridge to the PhD in Neuroscience Program (BPNP). The objectives of BNW are to identify promising students for BPNP, and to increase awareness of neuroscience as a discipline and a career option. BNW introduces basic concepts in neuroscience using a variety of educational techniques, including mini-lectures, interactive discussions, case studies, experimentation, and a sheep brain dissection. Since its inception in 2011 BNW has undergone a series of transformations that continue to improve upon an already successful and influential educational program for underrepresented minorities. As of Fall 2018, we have presented 21 workshops, impacting 200 high school and 424 undergraduate students. BNW has been offered at University of Puerto Rico (UPR)-Arecibo, UPR-Cayey, UPR-Humacao, Pontificia Universidad Católica de Ponce, and Universidad Interamericana de Puerto Rico-Arecibo. A pre-and post evaluation was given to evaluate material comprehension and thus measure effectiveness of our one-day interactive workshop. Our results suggest that both high school and undergraduate students have little prior knowledge of neuroscience, and that participation in BNW improves not only understanding, but also enthusiasm for the discipline. Currently, our assessment has only been able to evaluate short-term effects (e.g. comprehension and learning). Therefore, our current focus is developing methods capable of determining how participation in BNW impacts future academic and career decisions.

## Introduction

The lack of ethnic diversity in neuroscience is a persistent problem. Most minority groups, including Hispanics, are underrepresented at all levels of higher education academic pipeline as compared to their corresponding representation in the U.S. population. In 2013 the National Science Foundation reported a total of 795 doctorate recipients in the field of neuroscience corresponding to US citizens and permanent residents. Hispanics comprised only 7.8% of degree recipients, but encompass 17.1% of the U.S. population [1]. This discrepancy becomes more pronounced at the level of tenured and tenure-track faculty. Among the top 100 U.S. biological science departments, Hispanics account for only 4.3%, 2.6%, and 1.8% of assistant, associate, and full professors, respectively [2]. This lack of ethnic diversity is of significant consequence. As a scientific community, we are losing critical talent and diverse perspectives that enhance creativity and innovation in scientific endeavors. If not remedied, underrepresentation of minorities could contribute to the loss of our country’s global technical and intellectual leadership.

One strategy to tackle the underrepresentation of minorities in neuroscience is community outreach and science education targeting specific minority populations during the formative stages of their academic scholarship. The University of Puerto Rico (UPR) public collegiate system is an ideal population of Hispanic students from which to recruit future neuroscientists. UPR consists of 11 campuses located throughout the Commonwealth of Puerto Rico, a U.S. territory in the northeastern Caribbean. Between 2009 and 2013, UPR became the 2^nd^ leading university to award doctoral degrees to Hispanic recipients [3]. Despite this achievement, exposure to neuroscience at the collegiate level in the UPR system remains low. A search of UPR curricular offering through the official UPR webpage reveals that only 2 of the 10 sub-graduate campuses that grant bachelor of science degrees offer a course specifically in neuroscience: UPR-Cayey and UPR-Rio Piedras. UPR-Rio Piedras has a National Institute of Health Enhancing Neuroscience Diversity through Undergraduate Research Education Experiences (NIH-ENDURE) program since 2011 which provides research opportunities in neuroscience to students in their institution. Outside of the UPR system, there are 2 private institutions with a neuroscience course as part of their bachelor of science curriculum: University of Turabo and Pontifical Catholic University of Puerto Rico. In 2015 the Bayamón Central University established a Bachelor’s Degree in Neuroscience, making it the first and only undergraduate institution in Puerto Rico with an academic program specializing in neuroscience. More recently, 2016, NeuroBoricuas was launched as a program that provides workshops in neuroscience related topics to K-12 and the community in general.

The extent to which neuroscience-related courses are offered in K-12 educational curricula in Puerto Rico may also contribute to the scarcity of Hispanic representation in the neurosciences. The public K-12 Science Program in Puerto Rico focuses on the scientific method and understanding the rationale for this process of investigation. The program classifies the sciences into four domains: life, physical, terrestrial, and spatial. Core concepts from each domain are first introduced at the elementary level, and then expanded upon at the intermediate and high school levels. Although it is plausible that specific science courses cover concepts in neuroscience, these topics would most likely be introduced superficially at the high school level. Students attending a subset of public magnet schools for science and mathematics have the highest likelihood of exposure to the neurosciences. These institutions offer more advanced science disciplines, such as organic and inorganic chemistry, genetics, microbiology, biochemistry, human anatomy and physiology. Therefore, although these schools still do not offer a specific course for neuroscience, basic concept in neuroscience are undoubtedly introduced (Author’s Note: This information was obtained from the official public policy about the organization and curricular offering for the Science Program in elementary, intermediate and superior levels of public schools in Puerto Rico) [4].

Taken together this information suggests that neuroscience as a discipline is rarely covered in educational institutions in Puerto Rico. In an effort to overcome this omission, we developed the Bridge to Neuroscience Workshop (BNW), a full-day hands-on workshop in neuroscience education. The BNW was conceived as an auxiliary component of a parent recruitment program called Bridge to the PhD in Neuroscience Program (BPNP). BPNP was developed by Dr. William D. Atchison at Michigan State University in 2010 after 12 years of collaboration with the UPR-Cayey. BPNP is a 4-year program spanning the final two years of undergraduate studies and the first two years of graduate studies. BPNP aims to provide professional development, neuroscience related research experience, increased awareness of neuroscience as a prospective discipline, and facilitate top-down student-directed mentorship.

As a supplement to BPNP, BNW was designed to identify promising students for BPNP, and to increase awareness of neuroscience as a discipline and a viable career option. BNW, in collaboration with BPNP, has helped identify and recruit promising students since its inception in 2011.

The purpose of this article is to (1) provide educators and students with access to the BNW educational materials to continue neuroscience education; and (2) provide evidence demonstrating the effectiveness of BNW as a tool to engage high school and undergraduate students in neuroscience education. As such, we describe the components of BNW including educational materials and activities. Secondly, we evaluate previous participants’ performance on pre- and post-evaluations, and discuss participant feedback.

### Bridge to neuroscience workshop

BNW was designed by nine graduate students at Michigan State University, under the direction of Dr. William D. Atchison. These graduate students wrote a grant to obtain funding for workshop materials and travel expenses, designed lecture and activity materials, and wrote the accompanying workbook. Additionally, most of these graduate students traveled to Puerto Rico on at least one occasion to lead the workshop.

#### Content description

BNW is comprised of: a) a written entrance evaluation given upon arrival to assess students’ prior knowledge of neuroscience; b) the workshop, which includes 4 sessions: 1) “Getting to Know Your Nervous System”, 2) “Your Nervous System at Work”, 3) “Common Diseases of the Nervous System”, and 4) “Sheep Brain Dissection”; and c) an exit evaluation given to assess comprehension of material discussed throughout the day.

#### Entrance and exit evaluation

The entrance and exit evaluations are described in the methods section below.

#### Workshop session 1: Getting to know your nervous system

The workshop begins with a session entitled “Getting to Know Your Nervous System”, in which basic concepts of the central nervous system are taught through mini-lectures and interactive activities. First, a “Matching Activity” uses comparative neurobiology to discuss gross anatomical features of the brain. Students correlate particular anatomical features with function to identify the brains of eight different animals. Next students model the basic structure of a neuron in the “Giant Rope Neuron” activity [5]. Then students utilize their working model to perform action potentials and synaptic transmission. In the final activity of the first session, students explore basic electrophysiology associated with action potentials using the SpikerBox [6], a device that allows students to visualize and listen to action potentials firing from the leg of a cockroach.

#### Workshop session 2: Your nervous system at work

The second session, “Your Nervous System at Work”, is an experiment-driven exploration of the sensory, motor, and autonomic systems, which expands upon the concepts introduced during Session 1. Students learn how to formulate testable hypotheses, design experiments, make observations, and report results. “Tasting With Your Nose” [5] examines the hypothesis that sensory systems integrate to produce particular perceptual experiences, in this case taste. “Reaction Time” [5] investigates how the central nervous system assimilates sensory input to regulate movement. Finally, “Experimentation with Blood Pressure”, gives students an opportunity to formulate their own unique hypotheses, and design experiments to test how specific environmental factors (e.g. caffeinated drinks, exercise, relaxation, etc.) modulate blood pressure (S1 and S2 Files).

#### Workshop session 3: Common diseases of the nervous system

The third session includes a mini lecture and case studies for the identification of “Common Diseases of the Nervous System”. In this session students learn about the symptoms, mechanism of pathogenesis, pathology, diagnosis and treatment of Parkinson’s disease, Alzheimer’s disease, Amyotrophic Lateral Sclerosis and stroke. The session starts with a brief lecture on the symptoms and pathology of each neurological disease, once finished the students are assigned a case study (1 per group of 5 students) and charged with identifying the neurological disease. A closing discussion explains mechanisms of pathogenesis and available treatments for each disease (S3 File).

#### Workshop session 4: Sheep brain dissection

The final session of the workshop is the “Sheep Brain Dissection”. The sheep brain dissection is an opportunity for students to explore a real brain. Participants are able to identify the structures of the brain discussed throughout the day and also perform dissections in order to identify internal structures.

#### Educational materials

In addition to workshop discussions and activities, a workbook is used throughout the workshop sessions to supplement oral lessons and engage the students (S4 and S5 Files). This booklet contains a summary of all the topics covered in the workshop, including organization of the nervous system, structure and function of neurons, generation and propagation of action potentials, synaptic transmission, sensation, movement, autonomic function, and diseases of the nervous system. There are descriptive figures, thought-provoking questions, and space available for data collection and observational notes associated with each activity or experiment.

Importantly, the workbook is written in English and Spanish to facilitate the understanding of material for students who are not fluent in English. BNW is conducted entirely in English in order to give students an opportunity to practice using the language in an academic setting. However, because most of the material is novel and challenging, without the aid of the Spanish workbook, attendee’s comprehension and ultimately their engagement in the workshop may have been hindered. At the end of the day, workshop attendees are encouraged to take their workbook home as an educational resource.

## Methods

### General description

**Fig 1:**
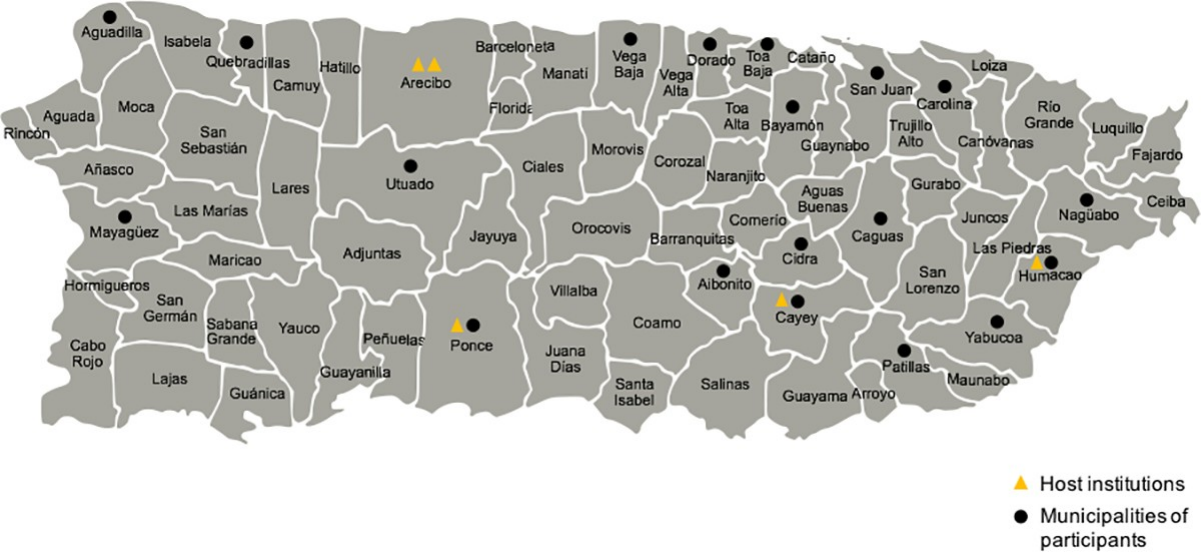
Map of Puerto Rico indicating the municipalities of BNW participating institutions. The municipalities of Arecibo, Cayey, Humacao and Ponce have hosted the 21 previously conducted workshops. The undergraduate institutions that served as hosts are; the University of Puerto Rico-Arecibo, Universidad Interamericana de Puerto Rico-Arecibo, University of Puerto Rico-Cayey, University of Puerto Rico-Humacao, and the Pontificia Universidad Católica de Ponce. High schools from 12 different municipalities have participated in the workshops. This is a representative map of Puerto Rico’s municipalities and it is for illustrative purposes only.

Since 2011 a total of 21 workshops have been conducted at five different sub-graduate institutions in Puerto Rico. The five institutions that have served as hosts for BNW are: University of Puerto Rico at Cayey (UPR-Cayey) and University of Puerto Rico at Arecibo (UPR-Arecibo), University of Puerto Rico at Humacao (UPR-Humacao), Pontifical Catholic University of Puerto Rico in Ponce and Universidad Interamericana de Puerto Rico in Arecibo (Fig 1). The workshops were conducted Saturday, Sunday or Monday (normal school day) from 9:00 am to 5:00 pm. Although multi-day learning experiences are inherently a more robust educational strategy, we designed BNW as a one-day workshop because of limited availability at host institutions. Weekday workshops would have interfered with regularly scheduled university courses and reduced student participation.

### Participants and volunteers

BNW participants included both high school and undergraduate students. High school participants were recruited by contacting science teachers employed at local schools surrounding host institutions and by word of mouth. Undergraduate participants were recruited primarily from the host institutions, through our website (https://www.msubpnp.com/) and Facebook page (Bridge to PhD in Neurosciences at Michigan State University). However, announcements were also made at neighboring collegiate institutions to encourage additional undergraduate participation. Recruited students were required to submit a signed parental authorization form through the program email prior to participation. High school science teachers from invited schools were strongly encouraged to participate by providing a certification of workshop completion and a monetary compensation.

Each workshop accommodated up to 30 students, and the same workshop was conducted for high school or undergraduate students, however high school and undergraduate student populations were never mixed. Participants were divided into small laboratory groups of 4–5 students. Embedded within each laboratory group was an undergraduate Puerto Rican student who had either previously attended BNW as a participant, or was currently enrolled in the parent BPNP. These advanced undergraduate students ensured that workshop participants understood the material being presented and assisted participants with BNW activities and experiments. Workshops were led by 3–4 graduate students recruited from the Michigan State University Neuroscience Program, the Department of Pharmacology & Toxicology, and the Comparative Medicine & Integrative Biology Program. These graduate students introduced the sessions with mini-lectures, demonstrated activities, and facilitated group discussions.

### Feedback from participants

#### Evaluations

All participants were given 15 minutes to complete a short evaluation at the beginning (pre-test) and at the end (post-test) of the workshop. The tests consisted of 11 multiple choice and short answer questions addressing recall and comprehension of the discussed material (S6 File). The following core concepts were assessed: unique structure and function of brain and neurons, generation and conduction of electrical signals, synaptic transmission, perception and integration of sensory stimuli, and role of autonomic nervous system in the control of fight-or-flight response. Evaluations were scored blinded to student ID as well as to the pre- and post-test score using a rubric (S7 File). Differences in the mean percentage of correct, incorrect, and incomplete responses between pre- and post-tests within high school and undergraduate students were compared and statistical significance was determined using two-way analysis of variance (ANOVA). When the omnibus test met the criterion for significance Sidak’s post-hoc test was used to make all possible comparisons. All tests were two-tailed, and the criterion for significance was set at *p* < 0.05. GraphPad Prism 6 software (GraphPad software, Inc.) was used for all statistical tests.

#### Feedback form

Feedback forms were administered at the end of the workshop to gauge interest and seek recommendations. These forms were purposefully short and open-ended, asking participants to describe two things that they liked from the workshop and one thing that they would change (“Two Stars and a Wish”). Feedback and pre- and post-test evaluation responses were anonymous; no personal information was gathered from any participant in any of the conducted workshops.

## Results

**Fig 2:**
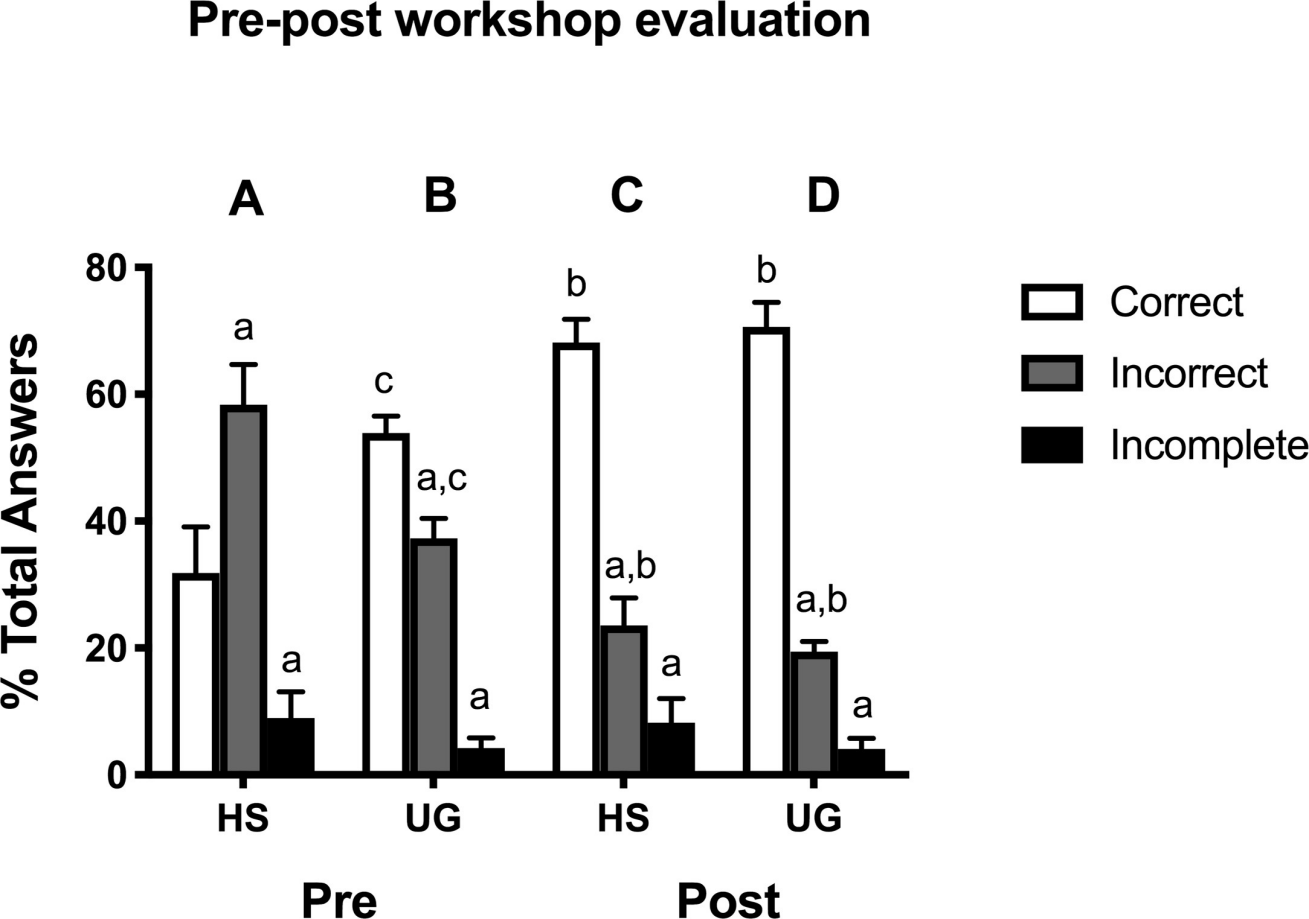
Comparison between high school and undergraduate knowledge about neuroscience prior to and after participation in BNW. High school students had a significantly higher mean percentage of incorrect answers prior to BNW attendance (A). Undergraduate students scored significantly higher on the pre-test as compared to high school students (B). Following completion of BNW, the number of correctly answered question increased significantly for high school (C) and undergraduate (D) students. a indicates a significant difference from “correct” within group. b indicates a significant difference from pre-test response within student population. c indicates a significant difference from high school pre-test group within response.

In order to identify students’ knowledge and understanding of neuroscience prior to the workshop and after the workshop an entrance (pre-test) and exit evaluation (post-test) was given. The evaluations had the same questions in order for us to make a direct comparison of the understanding of the neuroscience concepts taught, before and after the workshop. Evaluations were given in all 21 workshops by lead mentors. However, we do not have responses for all 624 attendees as evaluations that were left blank were not considered. In addition, several evaluations were lost before they were collected and shipped to Michigan State University for analysis. Responses from 129 high school and 303 undergraduate students are presented in Fig 2. Our data can be found on the open science framework under bridge to neuroscience workshop.

### Pre- and post-test evaluations

High school participants answered significantly more questions incorrectly (58.4 ± 6.3%) than correctly (31.8 ± 7.2%; p = 0.003) on the pre-test, and left 9.0 ± 4.1% of pre-test questions incomplete (Fig 2A). Conversely, undergraduate participants answered 54.4 ± 2.8% of the total questions correctly and 41.0 ± 2.2% incorrect (p = 0.0001), leaving 6.0 ± 2.0% incomplete (Fig 2B).

Post-test responses were used to assess participants’ comprehension of material discussed during the workshop. After attending BNW, the mean percentage of correctly answered questions increased from 31.8 ± 7.2% (pre-test) to 68.2 ± 3.7% (post-test) for high school students (p<0.0001; Fig 2C), and from 54.4 ± 2.8% (pre-test) to 73.8 ± 2.4% (post-test) for undergraduate students (p<0.0001; Fig 2D). Accordingly, the mean percentage of incorrectly answered questions on the post-test decreased from 58.4 ± 6.3% (pre-test) to 23.6 ± 4.3% (post-test) for high school students (p = 0.0001; Fig 2A v. 2C) and from 41.0 ± 2.2% (pre-test) to 20.6 ± 1.5% (post-test) for undergraduate students (p<0.0001; Fig 2B v. 2D). The mean percentage of incomplete answers did not differ significantly between the pre- and post-tests for either high school (8.9 ± 4.1% (pre-test) vs. 8.2 ± 3.8% (post-test); p>0.05) or undergraduate students (6.0 ± 2.0% (pre-test) vs. 5.6 ± 1.9% (post-test); p>0.05).

### High school and undergraduate participant performance on pre- and post-test evaluations

Comparison of high school and undergraduate participant pre-test performance revealed significant differences in prior knowledge between these cohorts (Fig 2A & 2B). Undergraduate students scored significantly more questions correctly on the pre-test (54.3 ± 2.8%) as compared to high school students (31.8 ± 7.2%; p = 0.0002). As such, the mean percentage of incorrect answers on the pre-test was significantly higher for high school students (58.4 ± 6.3%) than undergraduate students (41.0 ± 2.2%; p = 0.005). There was no difference in the mean percentage of incomplete pre-test responses between high school (8.9 ± 4.1%) and undergraduate students (6.0 ± 2.0%; p>0.05).

Post-test high school and undergraduate performance was very similar (Fig 2C & 2D). There was no significantly difference between the cohorts for the mean percentage of correct answers (68.2 ± 3.7% (high school) vs. 73.8 ± 2.4% (undergraduate); p>0.05), incorrect answers (23.6 ± 4.2% (high school) vs. 20.6 ± 1.5% (undergraduate); p>0.05), or incomplete answers (8.2 ± 3.8% (high school) vs. 5.6 ± 1.9% (undergraduate); p>0.05).

**Fig 3:**
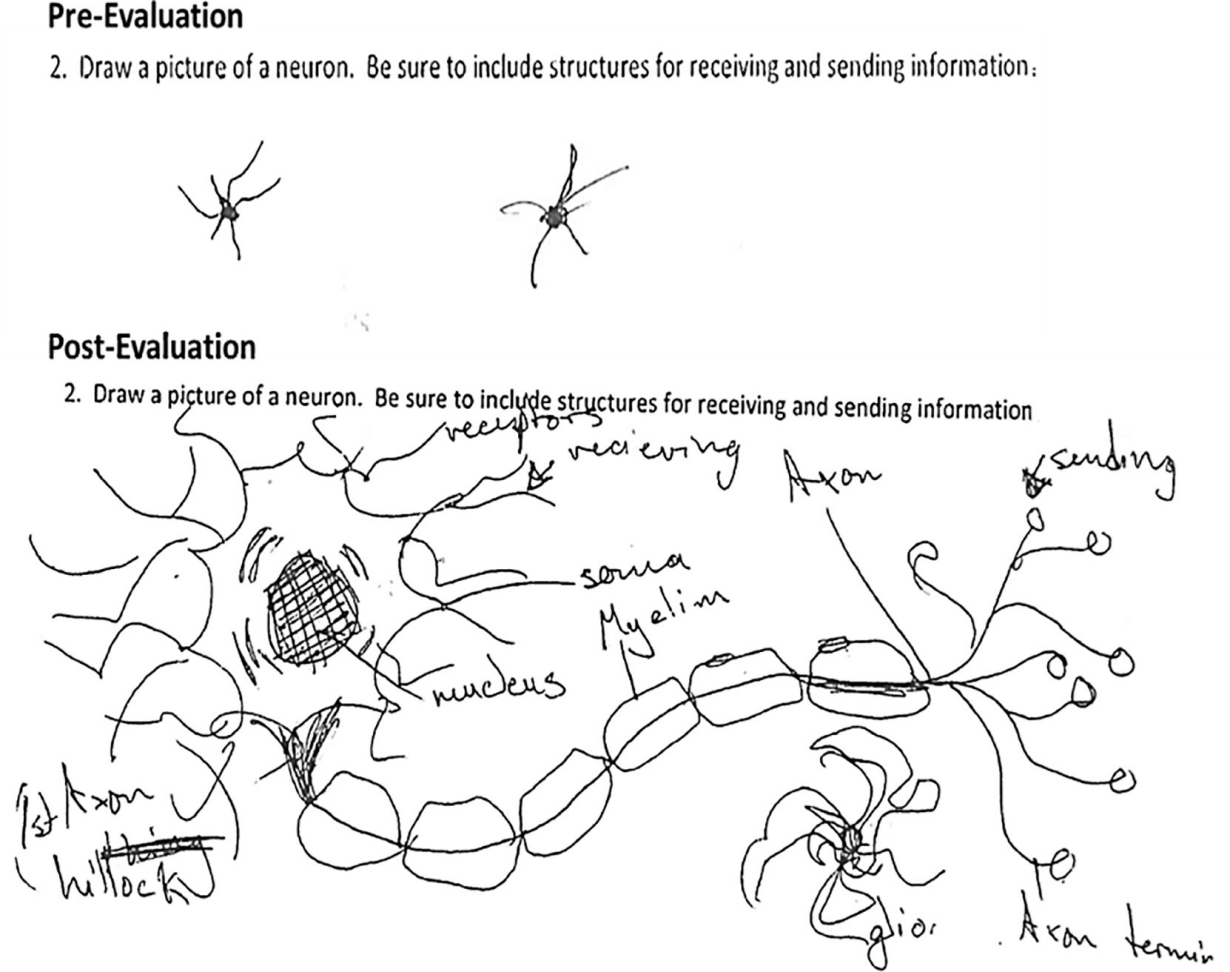
Representative response of a BNW participant. These drawings are the answer of a BNW participant to a question addressing understanding of the unique structure and function of neurons concept. The question is as follow: *Draw a picture of the neuron*. *Make sure to include structures for receiving and sending information*.

A representation of one pre- and post-test answer is shown in Fig 3. This question aimed to address understanding of the unique structure and function of neurons by asking the student to draw a model of a neuron including structures used for sending and receiving information. In the pre-test evaluation, the student had only a vague interpretation of a neuron. The student’s response during the post-test evaluation illustrates a much more comprehensive understanding of the structure and function of a neuron.

### Feedback form results

Our feedback form, “Two Stars and a Wish”, asked participants to describe two aspects of the workshop they enjoyed or found interesting, and one thing they would have changed. Responses from 336 students are summarized in Table 1. Participants enjoyed the hands-on experiments and activities (18%), liked the lecture topics (12%) and the interactive nature of the workshop (12%). Recommended changes included the suggestion to divide the workshop and have shorter lectures (14%), add more case studies for the “Common Diseases of the Nervous System” activity (11%), and implementing follow-up workshops (10%). Importantly, 21% of attendees indicated they would not change anything about the workshop. These responses and suggestions are being taken into consideration as we continue to modify the content for future workshops.

**Table 1:** Most common two stars and a wish response from 336 students.

Two Stars and a Wish	Comment from students	%
**Two things they liked from BNW**	Enjoyed the experiments & activities	18.3
	Like the lecture topics	12.8
	Llike the interactive, dynamic nature of the workshop	12.1
	Explanations were easy to understand/ Well organized	10.1
	Loved the dissection of the brain	9.8
	Like the neurological disease lecture and case studies	7
	Like the enthusiasm and energy of the presenters, knowledge of presenters	6.3
	Like the giant rope neuron activity	4.6
	Thought there was a good distribution of time and the workshop was well organized	1.9
	Like the jelly bean activity	1.7
**One thing they would change**	Would not change anything	21.2
	Divide the workshop and have shorter lectures	13.6
	Add more case studies of neurological diseases	11.2
	Increase the number of workshops, a whole series of workshops instead of only one day	10.4
	Add more hands on activities	10
	Use brains from different species for the brain dissection activity	5.2
	Would like to see a real human brain	3.2
	Change the language to Spanish	2.8
	Talk about the summer opportunities at Michigan State University	2.4
	Allow more more time for participants to perform the brain dissection	2

### BNW participant impact

Twenty-one workshops have been implemented during 12 different weekends between the years of 2011–2015. In that time, 200 high school students from 35 different schools and 424 undergraduate students from more than 10 different institutions have attended BNW (S1 Table). In addition, 20 high school teachers participated with their respective schools. Summary of participants is shown in Table 2.

**Table 2:** Schools and undergraduate institutions participating in BNW from 2011–2015.

Year	Date	Host institution	Teacher attendees	High school (HS) or undergraduate (U) attendees	Total participants
**2011**	24-Sep	UPR-Cayey	5	HS	25
	25-Sep	UPR-Cayey	5	HS	25
**2012**	18-Feb	UPR-Cayey	4	HS	25
	19-Feb	UPR-Cayey	1	HS	25
	20-Feb	UPR-Cayey	0	U	25
	Fall	UPR-Arecibo	0	U	20
	Fall	UPR-Arecibo	0	U	20
**2013**	27-Apr	UPR-Cayey	0	U	25
	28-Apr	UPR-Arecibo	0	U	25
	21-Sep	UPR-Cayey	0	U	25
	22-Sep	UPR-Cayey	0	U	25
**2014**	13-Dec	UPR-Cayey	0	U	14
	14-Dec	UPR-Cayey	1	11 HS/ 11 U	23
**2015**	28-Feb	UPR-Humacao	2	HS	30
	28-Feb	UPR-Humacao	0	HS	32
	1-Mar	UPR-Humacao	0	U	25
	1-Mar	UPR-Humacao	0	1 HS/ 27 U	28
	10-Apr	Pontificia Universidad Católica de Ponce	0	U	19
	10-Apr	Pontificia Universidad Católica de Ponce	0	U	23
	11-Apr	Pontificia Universidad Católica de Ponce	2	13 HS/ 9 U	22
	11-Apr	Pontificia Universidad Católica de Ponce	0	5 HS/ 16 U	21
	9-May	Universidad Interamericana de Arecibo	0	U	22
	9-May	Universidad Interamericana de Arecibo	0	8 HS/ 12 U	20
	11-May	UPR-Arecibo	0	U	25
	21-Nov	Pontificia Universidad Católica de Ponce	0	U	31
	22-Nov	UPR-Cayey	0	U	25
**Totals:**	**21 workshops**	**5 undergraduate host institutions**	**20**	**200 HS/ 424 U**	**624 total participants**

## Discussion

The lack of diversity in the neurosciences is well documented [7], and poses a significant hindrance to the innovation of our research, the progress of our field, and the societal impact of our discoveries. Collectively, the neuroscience community is responsible for developing engaging solutions to encourage the participation of underrepresented minorities in the neurosciences, and science in general. With this in mind, we developed BNW. Our results demonstrate that a one-day, hands-on workshop is an effective tool for increasing awareness of neuroscience as a discipline and potential career option. In addition to documenting the impact of BNW, we have detailed the structure of our workshop and provided open access to all the educational materials used for BNW so that other educators and outreach coordinators may build upon our ideas to continue diversifying the sciences.

Results of the pre- and post-evaluations revealed that completion of BNW improves comprehension of neuroscience in both high school and undergraduate students. Prior to attending the workshop, most participants had only superficial exposure to core neuroscience concepts. BNW successfully improved comprehension of many basic facets of the neurosciences, as determined by improved performance on post-evaluation scores as compared to pre-evaluation scores. The observation that a one-day interactive workshop enriched participants’ comprehension of neuroscience principles is remarkable, and demonstrates that BNW is an effective learning tool. However, one limitation to the present analysis is the truncated timeline for evaluating learning. At present, we do not have the resources or infrastructure necessary to track BNW participants longitudinally, and thus we are only able to assess comprehension immediately after completion of the workshop. In the future, we aim to develop a longitudinal survey evaluating the long-term impact of BNW on learning retention and participant career trajectories.

Participant feedback demonstrated that the workshop topics, hands-on experiments and presenter enthusiasm are the most well received aspects of the workshop, and motivate participant engagement and learning. Although the majority of students enjoyed the topics and overall workshop design, one major suggestion was to restructure the first session, “Getting to Know Your Nervous System”. Initially, the first session was primarily lecture-based and had four short experiments spaced between lecture topics. Based on information gathered from the feedback forms, we were able to identify specific modifications to improve to the workshop, including the addition of breaks in between the lecture and session 1 experiments, as well as added time for the brain dissection. We added three 5 min breaks in session 1, and shortened the peripheral nervous system part in session 2 allowing more time for the brain dissection activity.

The active learning, experiment-based approach of BNW is one of its most notable strengths. The activities and experiments used in BNW (included in supplemental material) request participants to immediately reflect upon concepts discussed in the mini-lectures, and use that information to solve problems and answer questions. Active learning and the associated intrinsic motivation are well-established educational methods for improving retention [8,9]. An example of one interactive activity used in BNW is the Giant Rope Neuron [5], which asks participants to model a neuron, highlighting the main compartments and functions, using rope, plastic rings, ping-pong balls, and other small household objects. To successfully complete this activity, participants need to use what they just learned about neurons to create a working model and correctly explain the models’ components and functions. The design of application activities, such as the aforementioned, has also been performed by other groups to reinforce comprehension of the concepts by students especially in pre-collegiate academic levels [6,10]. In addition, activities in BNW invite students to practice the scientific process. Some of the activities are designed with the purpose of making students think about a problem, construct a hypothesis, design a method to test that hypothesis, collect observations, make conclusions, and report those to the rest of the group. These types of activities greatly inspire the inquisitive nature of students and have been used by others to engage them in the scientific process while improving their critical thinking and effective communication skills [11]. It has also been found that scientist classroom visits have a great impact in students attitude towards science [12].

An additional key characteristic of BNW is the organization of our instructional teams, which relies on top-down student-directed mentorship. Essentially, graduate student instructors lead the workshops, senior undergraduate students moderate small groups of workshop attendees, and the participants themselves collaborate to learn from one another. This type of infrastructure has numerous educational, training and recruitment benefits both between and within each tier.

At the foundation of our model, workshop attendees are organized into small laboratory groups in an effort to promote an inclusive, peer-learning environment. BNW requires these student groups to utilize concepts introduced during the mini-lectures to cooperatively solve problems proposed during the activities. For example, during the first workshop session, students learn about basic structural features of a neuron and how those features impart function. Immediately thereafter, they are given a variety of household objects, such as bowls, ropes, and small plastic balls, to model a neuron. Only by interacting with and learning from one another can the students successfully complete this task. Although the workshop facilitators guide them, the students generate the final product. This instructional design gives participants ownership of the knowledge they acquire without potential intimidators, such as language barriers or inexperience.

The middle tier of our leadership design is composed of senior undergraduate students embedded within each attendee laboratory group. These senior undergraduates are enlisted from the BPNP to assist the graduate student instructors. Their primary responsibility is to ensure the participants comprehend material being discussed and engage in workshop activities. For many of these senior undergraduates, assisting with BNW is their first teaching experience. In this sense, the workshop is a unique training opportunity for BPNP undergraduates to begin developing verbal pedagogical skills. However, these BPNP undergraduates serve an addition critical role as mentors to the BNW attendees. Because these senior undergraduates share a similar ethnic and educational background with most of the BNW participants, they exemplify the first attainable phase of training to become a neuroscientist.

At the top, graduate students gain valuable experience designing and implementing neuroscience-related educational activities at both the high school and collegiate level. Considering the paucity of comprehensive teaching opportunities in most graduate programs, BNW is an invaluable training opportunity for those graduate students seeking careers in education and community outreach. Additionally, these graduate students serve as role models to both the BPNP senior undergraduates and the BNW student participants.

One reason proposed to explain the lack of ethnic and racial diversity in science is the low number of minorities among faculty ranks [2]. Undergraduate students of color are less likely to enter a university that employs a low number of faculty of color [13]. Furthermore, minority students are less likely to pursue careers within scientific disciplines if they lack appropriate mentors and role models [2]. Thus, until minority students are exposed to mentors from similar ethnic/racial backgrounds, we will never comprehensively diversify scientific disciplines. BNW, together with BPNP, represents one attempt to break this cycle. At each workshop, attendees witness students, not unlike themselves, successfully engaging in scientific scholarship and research. With each year, our programs recruit additional minority students, and with each year, these students advance to more senior positions.

BNW efforts will continue to focus on increasing exposure of minority students to neuroscience through our workshop and through established collaborations in neuroscience research through the BPNP summer program.

### Future directions

BNW as an outreach activity will continue in Puerto Rico with the overall goal of reaching a greater population of students. This will be achieved by bringing BNW to new institutions located closer to a population of students unable to attend previously conducted workshops due to transportation issues. BNW will also be conducted in the contiguous United States starting in the Fall of 2019. Two workshops will be conducted during each weekend visit to host institutions. One workshop will be conducted on Saturday and one on Sunday. Each visit will allow for the conduction of a workshop for 30 students. We aim to increase our impact by increasing our exposure not only in Puerto Rico but now implementing the workshops in the mainland U.S.

## Supporting information

S1 Table List of colleges and high schools of participants that have attended BNW. (DOCX)

S1 File BNW activities handout in English. (DOCX)

S2 File BNW activities handout in Spanish. (DOCX)

S3 File Neurological disease case studies. (DOCX)

S4 File BNW Workbook in English. (DOCX)

S5 File BNW Workbook in Spanish. (DOCX)

S6 File BNW Pre-and post-evaluation template. (DOC)

S7 File BNW Pre-and post-evaluation rubric. (DOC)
