# Supplementary material for: Bridge to neuroscience workshop: An effective educational tool to introduce principles of neuroscience to Hispanics students
Source: PLoS One. 2019 Dec 12;14(12):e0225116. doi: 10.1371/journal.pone.0225116 (PMC6907774; doi:10.1371/journal.pone.0225116)
Supplement: S1 Table — (DOCX) [file pone.0225116.s001.docx]

**S1 Table**. List of colleges and high schools of participants that have attended BNW.
