# Supplementary material for: Bridge to neuroscience workshop: An effective educational tool to introduce principles of neuroscience to Hispanics students
Source: PLoS One. 2019 Dec 12;14(12):e0225116. doi: 10.1371/journal.pone.0225116 (PMC6907774; doi:10.1371/journal.pone.0225116)
Supplement: S1 File — (DOCX) [file pone.0225116.s002.docx]

**YOUR NERVOUS SYSTEM AT WORK**

**Sensory Systems**

**EXPERIMENT #1: Jellybean Taste Test**

We use our **sensory system** to navigate and interact with the world around us. Humans have several sensory systems. You are probably familiar with the five basic senses – vision, hearing, touch, taste, and olfaction/smell. Other sensory systems include pain, balance, and temperature. Each sensory system consists of sensory receptors, a neural pathway, and a region of the brain devoted to sensory interpretation and perception. Sensory receptors detect a specific type of sensory stimulus (e.g. light, sound waves, chemical odorants) and convert the stimulus into an action potentials. A neural pathway then relays the electrical signal to the brain. Most sensory information is first filtered through an area of brain called the thalamus. From there the information is further processed in specialized areas in the cortex that are specific for different types of sensory information (Fig 14). However, there are connections between these areas which give a complete sensory perception of the environment.

**Figure 14. Summary of the major sensory systems in the human body.**

In this experiment we will explore how individual sensory systems work together. The perceptions of smell and taste begin when chemical molecules detach from substances and float into the nose or are put into the mouth where they are dissolved and bind to sensory receptors. Separate neural pathways transmit sensory information from the nose or mouth to the brain where we perceive odors or tastes, respectively. Although the neural systems (sensory receptors, neural pathways, and primary brain centers) for taste and smell are distinct from one another, the sensations of flavors and aromas often work together.

**MATERIALS:**

- Jellybeans

**METHODS:**

Work with a partner. Take turns being the experimenter and the subject. The experimenter gives the subject one jellybean according to the three conditions listed below. The subject guesses the jellybean flavor after each condition. The experimenter records two things: (1) what color (flavor) the jellybean is **before** giving it to the subject and (2) what the subject reports as the flavor after each condition. The three conditions are:

(1) Eyes closed and nose plugged

(2) Eyes closed and nose unplugged

(3) Eyes open and nose unplugged

**HYPOTHESIS:** *[Consider the following questions: Under which conditions do you think your partner will be able to correctly determine the jellybean flavor? Why?]*

**OBSERVATIONS:**

| **SUBJECT** | **CONDITION #1 GUESS** | **CONDITION #2 GUESS** | **CONDITION #3 GUESS** |
| --- | --- | --- | --- |
|  |  |  |  |
|  |  |  |  |

**CONCLUSION:**

**EXPERIMENT #2: Two Point Discrimination**

Sensory receptors in our skin allow us to identify several distinct types of tactile sensations, such as tapping, vibration, pressure, pain, and temperature. What allows us to make these distinctions? First, human skin contains many different kinds of sensory receptors that respond preferentially to various mechanical, thermal, or chemical stimuli, and then convert that specific type of sensory stimuli into action potentials (Fig 15A). Distinct types of tactile information are then transmitted to the brain along specific neural pathways where they converge in a brain region called the **somatosensory cortex**.

**A**


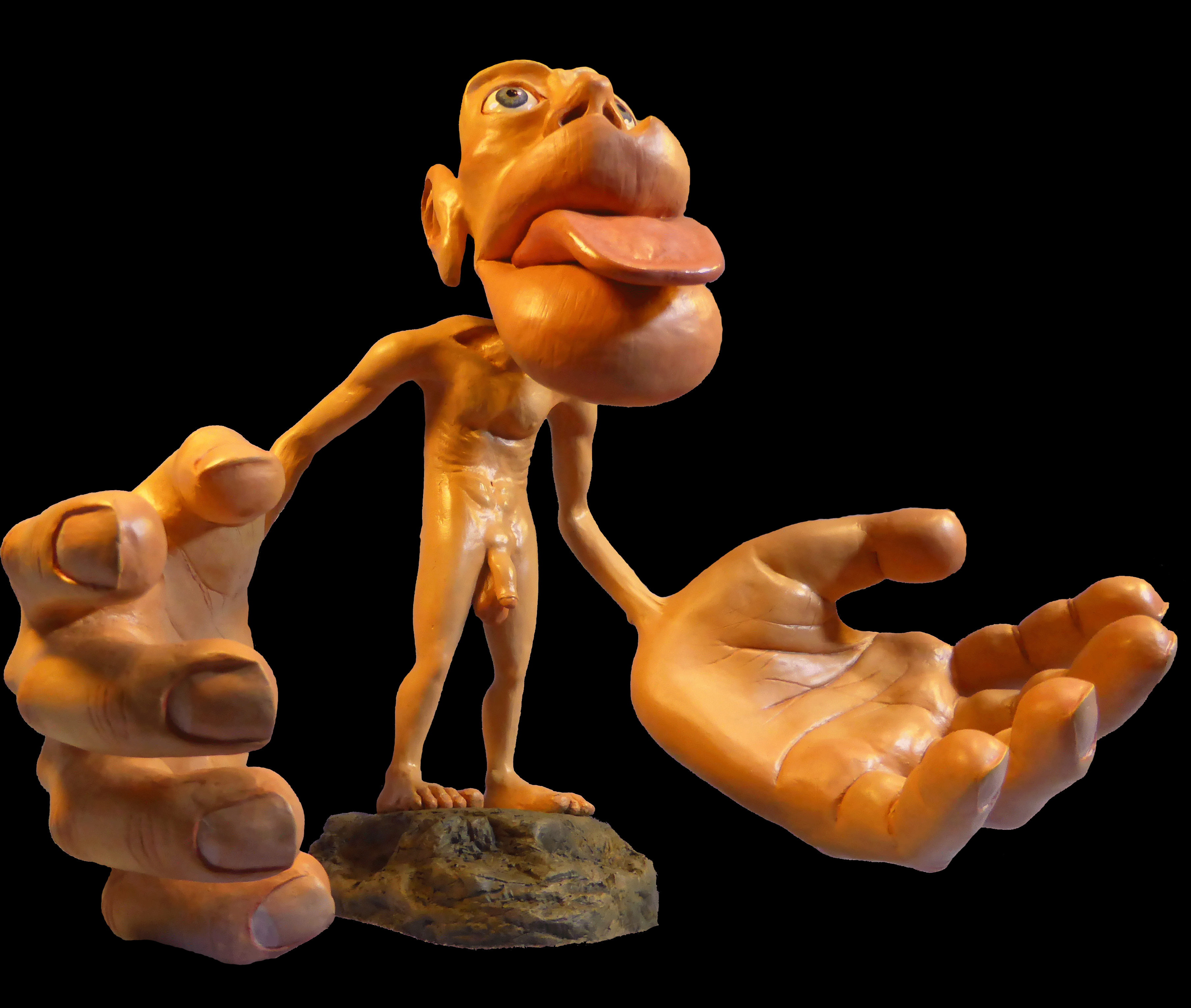
Sensory input is “mapped” along the somatosensory cortex in a **somatotopic arrangement**. This means that sensory information from each area of the body corresponds to a specific area of the somatosensory cortex. Furthermore, information is organized according to location. For example, neurons in the somatosensory cortex that receive input from the right thumb neighbor neurons that receive input from the right index finger. In this way, a sensory "map" of the body surface is created on a section of the brain surface. *Somatotopy* also means that areas of the body that are finely controlled (i.e. have more sensory receptors within a smaller surface area) have larger portions of the somasensory cortex, whereas regions of the body under coarse control have smaller portions of the somatosensory cortex. For example, the fingertips contain about 100 times more receptors per square centimeter than the skin on the back. Because of this, more neurons must be devoted to receiving fingertip sensations, and consequently the cortical area that receives input from the fingertips is much larger than to the area that receives input from skin on the back. The *sensory homunculus* is a distorted image of the human body that reflects the relative space each body parts occupies on the somatosensory cortex (Fig 15B). In this experiment, we will test the sensation of touch, and evaluate which areas of the body are under fine and coarse control.

**Figure 15. A) Representation of the different types of sensory receptors and the type of sensory information they detect. B) The sensory homunculus** (image by Mpj29 shared under the Creative Commons Attribution-Share Alike 4.0 International license).

**B**

**MATERIALS:**

- Drawing compass

**METHODS:**

Work with a partner. Take turns being the experimenter and the subject. The subject closes his/her eyes and sits still. The experimenter uses the compass to lightly touch the two compass points simultaneously to their partner’s skin. The experimenter then asks the subject if he/she feels one or two points. The experimenter should begin with the compass points at a great distance and continue to adjust the compass points closer and closer together until the subject reports feeling only one point. The experimenter records the smallest distance between the two points of the compass at which the subject can detect both points.

**HYPOTHESIS:**

**OBSERVATIONS:**

| **SKIN AREA TESTED** | **MINIMUM DISTANCE FOR**  **TWO POINT**  **DISCRIMINATION**  in millimeters (mm) |
| --- | --- |
| Tip of index finger |  |
| Thumb |  |
| Palm |  |
| Cheek |  |
| Forehead |  |
| Back of calf/lower leg |  |
| Forearm |  |


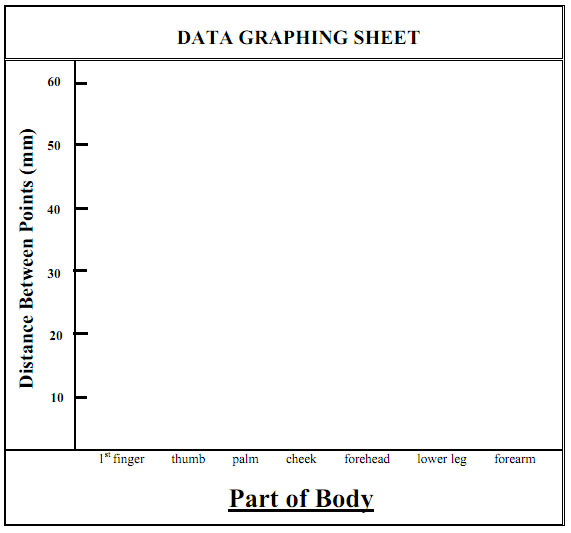


**CONCLUSIONS:**

**QUESTIONS:**

1. What determines our ability to discriminate two points?

2. What is the relationship between the number of sensory receptors in the skin and the size of the somatosensory cortex devoted to interpreting sensation from that body area?

3. How much of the somatosensory cortex is devoted to perceiving sensation in each of the regions you tested (circle one):

Index Finger Large Medium Small

Cheek Large Medium Small

Forehead Large Medium Small

Back of calf Large Medium Small

Forearm Large Medium Small

4. What could you do to change the perception of touch in this experiment? Would this change take place at the level of the skin or the brain?

**Motor System**

**EXPERIMENT #3: Patellar (Knee-Jerk) Reflex**

The **motor system** is the part of the nervous system responsible for the contraction and coordination of muscles. The neuromuscular junction (NMJ) is the area where the axon terminal of a motor neuron makes a connection with a muscle fiber. Upon receiving a signal from the brain, motor neurons release neurotransmitters at an NMJ to cause contraction of the muscle fiber. The more muscle fibers are activated by motor neurons, the stronger the magnitude of the contraction.

**Knee-Jerk (Patellar) Reflex**

There are different types of muscle (skeletal, cardiac, and smooth muscle) that have different functions in the body. Therefore, the motor system is responsible for a variety of tasks, including mechanical movement, heart muscle contraction, and intestinal muscle contraction.

**Figure 16. The knee-jerk patellar reflex test.**

The following exercise will demonstrate how reflex muscle contractions work. Reflexes are involuntary and almost instantaneous muscle movements that have an important role in the protection of the human body. Their fast onset is possible because they do not require integration and instruction from the brain. The knee-jerk or patellar reflex is one type of reflex muscle contraction (Fig 16). It will show you how the sensory and motor systems work together in a matter of milliseconds!

**MATERIALS:**

- Side of your hand
- A partner sitting on a table with legs hanging freely

**METHODS:**

Work with a partner. Take turns being the experimenter and the subject. The subject sits on a table, so that their leg is able to swing freely. The experimenter hits the subject’s leg just below the knee with the side of his/her hand.

**OBSERVATIONS:**

**CONCLUSIONS:**

**QUESTION:**

1. Why do doctors perform this test when you go for an annual check-up?

**EXPERIMENT #4: Reaction Time**

Effective communication between your sensory and motor systems enables you to protect yourself against harmful situations. Whenever someone throws a ball at you, your first instinct is to raise your arms to protect your body. If you touch something hot, you do not have to think twice about removing your hand from the hot object. These and many other reflex movements are dependent on the fast communication between neurons of the sensory system and neurons of the motor system. The speed of this communication process depends in part on the distance the signal has to travel.

In this experiment, we will examine the coordination between the visual sensory system and the motor system. You will be measuring your reaction time to a particular stimulus, which in this case is the drop of a ruler. When performing this exercise, keep in mind how important it is to have quick responses to stimuli every day.

**MATERIALS:**

- Ruler

**METHODS:**

Work with a partner. Take turns being the experimenter and the subject. The experimenter holds the ruler with the 0 inches marker at the bottom. The subject places their hand at the bottom of the ruler with the thumb and fingers around but not touching the 0 inches mark. The experimenter randomly drops the ruler 3 times, and the subject grasps it as quickly as possible (Note: The experimenter should not indicate or gesture when he/she will be dropping the ruler). Record the measurement just above the subject’s thumb where they catch the ruler each time. Convert reaction time from inches to seconds using the table available on the next page.

**HYPOTHESIS:**

**DATA COLLECTION:**

| **REACTION TIME (INCHES)** | | |
| --- | --- | --- |
| **TRIAL #1** | **TRIAL #2** | **TRIAL #3** |

| Distance | Time |
| --- | --- |
| **2 in** | 0.10 sec (100 ms) |
| **4 in** | 0.14 sec (140 ms) |
| **6 in** | 0.17 sec (170 ms) |
| **8 in** | 0.20 sec (200 ms) |
| **10 in** | 0.23 sec (230 ms) |
| **12 in** | 0.25 sec (250 ms) |
| **17 in** | 0.30 sec (300 ms) |
| **24 in** | 0.35 sec (350 ms) |
| **31 in** | 0.40 sec (400 ms) |
| **39 in** | 0.45 sec (450 ms) |
| **48 in** | 0.50 sec (500 ms) |
| **69 in** | 0.60 sec (600 ms) |

| **REACTION TIME (SECONDS)** | | | |
| --- | --- | --- | --- |
| **TRIAL #1** | **TRIAL #2** | **TRIAL #3** | **AVERAGE** |
|  |  |  |  |

**QUESTIONS:**

1. What factors or conditions would affect reaction time?

**CONCLUSION:**

**Nervous System**

**EXPERIMENT #5: External Factors That Modulate Blood Pressure**

The **autonomic division of the peripheral nervous system (ANS)** is responsible for control of internal viscera, cardiac muscle, and glands. There are two major divisions of the ANS: the **sympathetic** and **parasympathetic divisions of the ANS** (Fig 17). For most body functions, these two divisions work in opposite directions to accurately control organ function. For example, the sympathetic division increases heart rate, whereas the parasympathetic division decreases heart rate.

In this exercise, we’re going to explore how external factors, such as drugs or exercise, affect the two divisions of the ANS using blood pressure as an endpoint. You have been learning how to develop and test hypotheses, so here is your chance to formulate and test your own! Do you think caffeine activates the sympathetic or parasympathetic nervous system? How would you test this? What about exercise? Stress? Anything else?

**Figure 17. Peripheral Nervous System.**

**MATERIALS:**

- Blood pressure cuffs
- Caffeine drinks
- Stop watches
- Stairs
- Be creative: consider what items you have in your backpack, could those alter blood pressure somehow?

**METHODS:**

Work with a partner. Using the materials available (or anything else you have available) formulate a testable research question and hypothesize how some external factor (i.e. drug (caffeine), exercise, stress) affects the balance between the sympathetic and parasympathetic divisions of the ANS. Then test your hypothesis.

**HYPOTHESIS:**

**OBSERVATIONS:**

**CONCLUSIONS:**
