# Supplementary material for: Bridge to neuroscience workshop: An effective educational tool to introduce principles of neuroscience to Hispanics students
Source: PLoS One. 2019 Dec 12;14(12):e0225116. doi: 10.1371/journal.pone.0225116 (PMC6907774; doi:10.1371/journal.pone.0225116)
Supplement: S2 File — (DOCX) [file pone.0225116.s003.docx]

**CÓMO TRABAJA TU SISTEMA NERVIOSO?**

**Sistema Sensorial**

**EXPERIMENTO #1: Jellybean Taste Test**

Usamos nuestro **sistema sensorial** para navegar e interactuar con el mundo que nos rodea. El sistema sensorial humano incluye visión, audición, tacto, gusto, y olfato. El sistema sensorial puede recibir señales de una gran variedad de estímulos. Esta información sensorial es convertida en una señal eléctrica que es retransmitida al cerebro. La mayor parte de la información sensorial es filtrada en un área del cerebro llamada el **tálamo**. La información es procesada aún más en áreas especializadas del cerebro que procesan diferentes tipos de información sensorial (Fig 13). Sin embargo, estas áreas no están aisladas; existen conexiones entre estas áreas del cerebro que te permiten obtener una percepción sensorial completa del ambiente.

En este experimento vamos a explorar cómo los sistemas sensoriales individuales trabajan en sincronía. Los procesos de olfato y gusto surgen cuando moléculas químicas se separan de sustancias y flotan hacia la nariz o llegan a nuestra boca donde se unen a células sensoriales y estimulan células nerviosas. Estas células sensoriales transmiten mensajes a centros en el cerebro que nos hacen percibir olores y/o sabores. Aunque los sistemas de neuronas (células sensoriales, vías de nervios, y centros primarios en el cerebro) son distintos, las sensaciones de sabor y olores usualmente trabajan en conjunto.

**Figura 13. El Sistema Sensorial.**

**MATERIALES:**

- Jellybeans

**MÉTODO:**

Encuentra un/a compañero/a. Deberán turnarse para ser experimentador/a y sujeto. El experimentador le dará un jelly bean al sujeto bajo las tres condiciones especificadas abajo. El sujeto tratará de adivinar el sabor del jelly bean bajo cada condición. El experimentador apuntará dos observaciones: (1) de qué color (sabor) es el jelly bean antes de dárselo al sujeto, y (2) el sabor que el sujeto reporta bajo cada condición. Las tres condiciones son:

(1) Con los ojos y nariz cerrados

(2) Con los ojos cerrados, pero nariz abierta

(3) Con los ojos y nariz abiertos

**HIPÓTESIS:** [Considera las siguientes preguntas: ¿Bajo que condiciones tu crees que tu compañer@ va a poder determinar el sabor del Jellybean correcto? ¿Por qué?]

**OBSERVACIONES:**

| **SUJETO** | **SABOR BAJO CONDICIÓN #1** | **SABOR BAJO CONDICIÓN #2** | **SABOR BAJO CONDICIÓN #3** |
| --- | --- | --- | --- |
|  |  |  |  |
|  |  |  |  |

**CONCLUSIÓN:**

**EXPERIMENTO #2: Discriminación de Dos Puntos**

**A**


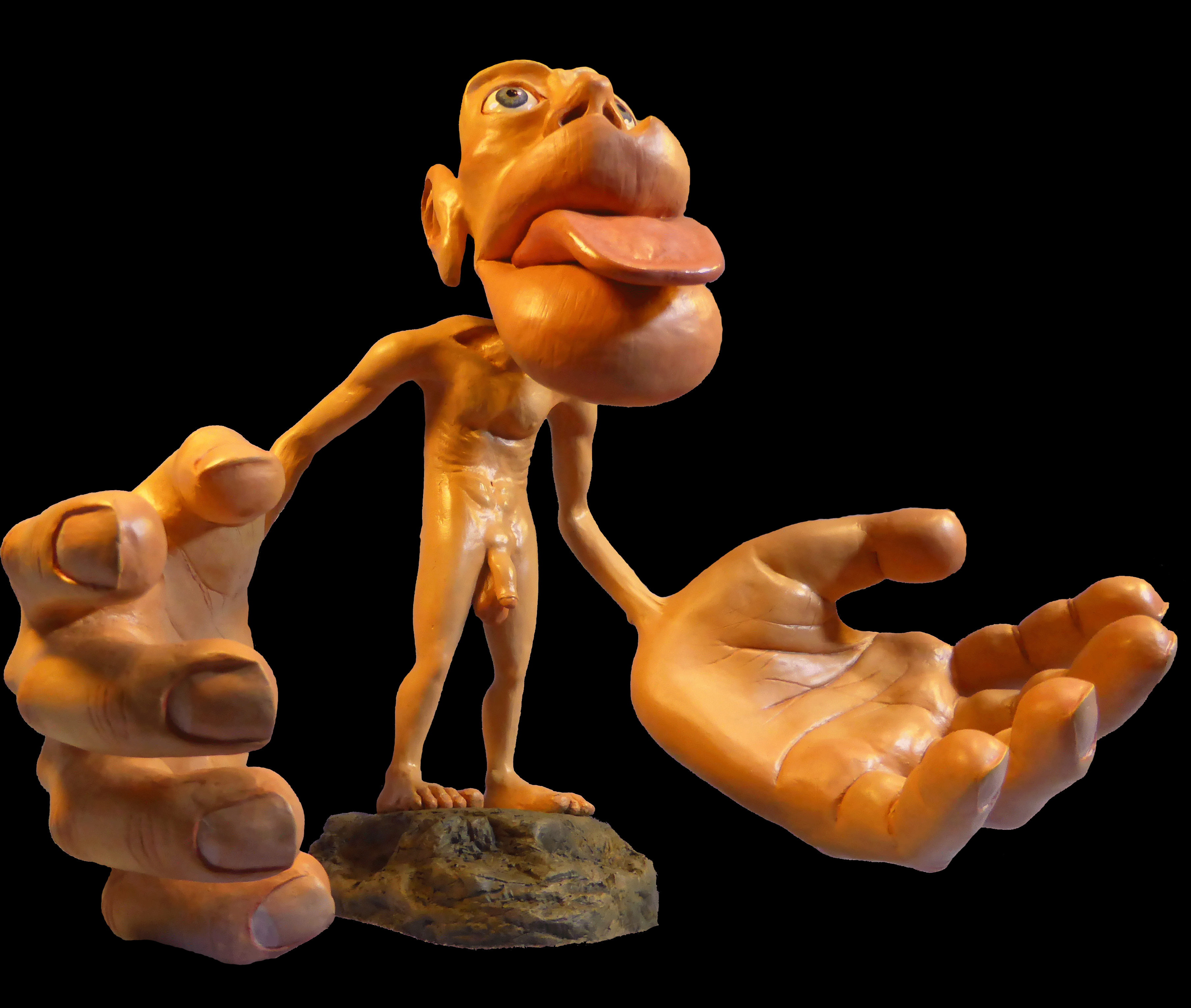
La información que obtenemos de nuestra piel nos permite identificar varios tipos de sensaciones, tales como tacto, vibraciones, presión, dolor, y temperatura. ¿Qué nos permite distinguir entre estos tipos de sensaciones? Primero, la piel humana contiene diferentes tipos de receptores sensoriales que responden preferentemente a varios estímulos mecánicos, termales, o químicos (Fig 14A). Segundo, las células sensoriales transmiten esta información al cerebro y médula espinal a través de nervios que convierten las energías mecánicas, termales, y químicas a señales eléctricas. La manera que interpretamos sensaciones depende no solo de las propiedades de los receptores sensoriales y neuronas, sino también de pasadas experiencias almacenadas en nuestro cerebro.

La información sensorial que recibe el cerebro se procesa en áreas específicas. La información de cada receptor en la piel se transporta a lo largo de una vía, formada de muchos axones de neuronas, a una parte del cerebro llamada la corteza somatosensorial. Esta área está empacada con neuronas que reciben la información de la piel en diferentes partes del cuerpo. La información sensorial es organizada en el cerebro de manera topográfica. Esto significa que las neuronas que reciben la información de las neuronas sensoriales en el dedo pulgar estarán localizadas en el cerebro en gran proximidad a las que reciben la información del dedo índice. De esta manera, un mapa sensorial de la superficie del cuerpo es creado en una sección de la superficie del cerebro. *El homúnculo sensorial* (Fig 14B) es una imagen que se utiliza para describir un modelo de un humano que refleja el espacio relativo que diferentes partes del cuerpo ocupan en la corteza somatosensorial. Por ejemplo, la punta de los dedos contiene 100 veces más receptores por centímetro cuadrado que la piel en tu espalda, y por tanto más neuronas del cerebro están dedicadas a recibir sensaciones de la punta de tus dedos. Consecuentemente, el área de la corteza somatosensorial que recibe información de la punta de tus dedos es mucho más grande comparada con el área que recibe información de la piel en tu espalda. En este experimento, vamos a examinar el sentido táctil de tu piel, que nos permite distinguir entre diferentes tipos de estímulos en la superficie de tu cuerpo.

**Figura 14. A) Representación de diferentes tipos de receptores y los tipos de sensaciones que detectan. B) El homúnculo sensorial** (imagen por Mpj29 compartida bajo la licencia Creative Commons Attribution-Share Alike 4.0 International).

**B**

**MATERIALES:**

- Compás

**MÉTODO:**

Consigue un compañero/a. Deberán turnarse para ser experimentador/a y sujeto. El sujeto cerrará sus ojos, mientras el experimentador usará el compás para tocar la piel del sujeto ligeramente. El experimentador le preguntará al sujeto si siente uno o dos puntos. El experimentador continuará ajustando el compás hasta que el sujeto reporte que solamente siente un punto. El experimentador apuntará cuál es la distancia más pequeña a la cual el sujeto pudo detectar los dos puntos.

**HIPÓTESIS:**

**OBSERVACIONES:**

| **ÁREA DE LA PIEL EXAMINADA** | **MÍNIMA DISTANCIA DE DISCRIMINACIÓN DE DOS PUNTOS**  En milímetros (mm) |
| --- | --- |
| Frente |  |
| Mejilla |  |
| Antebrazo |  |
| Palma de la mano |  |
| Dedo índice |  |
| Pantorrilla/pierna |  |
| Dedo pulgar |  |

**CONCLUSIONES:**

**PREGUNTAS:**

1. ¿Qué crees que determina tu habilidad de distinguir entre uno y dos puntos?

2. ¿Cuál es la relación entre el número de receptores en la piel y el tamaño de la corteza somato sensorial que está encargada de la interpretación de la sensación de esa parte del cuerpo?

3. ¿Cuánta área de la corteza somato sensorial está dedicada a recibir información sensorial de cada una de las regiones que investigaste? (circula una respuesta):

Dedo pulgar: Grande Mediana Pequeña

Mejilla: Grande Mediana Pequeña

Frente: Grande Mediana Pequeña

Pantorrila/pierna: Grande Mediana Pequeña

Antebrazo: Grande Mediana Pequeña

5. ¿Qué podrías hacer para cambiar la percepción de tacto en este experimento? ¿Harías este cambio a nivel de la piel o del cerebro?

**Sistema Motor**

**EXPERIMENTO #3: Reflejo Rotuliano**

El **Sistema Motor** es la parte del Sistema Nervioso que es responsable por la contracción y coordinación de los músculos. La unión neuromuscular es un área donde el terminal de un axón de una neurona motora conecta con una fibra muscular. Cuando reciben una señal del cerebro, las neuronas motoras liberan neurotransmisores a uniones neuromusculares para causar la contracción de fibras musculares. Mientras más fibras musculares se activan, más fuerte es la magnitud de la contracción.

Hay diferentes tipos de músculos (esquelético, cardiaco, músculo liso) con diferentes funciones en el cuerpo. Por tanto, el Sistema Motor es responsable por diferentes tareas tales como movimientos mecánicos, contracción del músculo cardiaco, contracción del músculo intestinal, entre otras.

**Figura 15. Reflejo rotuliano o patelar.**

El siguiente ejercicio demostrará cómo las contracciones de reflejo muscular ocurren. Los reflejos son movimientos musculares involuntarios y casi instantáneos que tienen un rol importante en la protección del cuerpo humano. Su rápido inicio de acción se debe a que no requieren del análisis e instrucción del cerebro para que ocurran. El reflejo rotuliano o patelar es un tipo de reflejo muscular (Fig 15). Te enseñará cómo el sistema sensorial y motor trabajan juntos en cuestión de milisegundos!

**MATERIALES:**

- Tu mano
- Una persona sentada con sus piernas cruzadas

**MÉTODO:**

Haz que tu compañer@ se sienta con sus piernas cruzadas, de manera que su pierna pueda oscilar libremente. Golpea a tu compañer@ justo debajo de su rodilla con el lado de tu mano. Si lo haces correctamente, la pierna de tu compañer@ dará una patada casi inmediatamente.

**OBSERVACIONES:**

**CONCLUSIONES:**

**PREGUNTA:**

1. ¿Por qué los doctores verifican la función del reflejo rotuliano/patelar?

**EXPERIMENTO #4: Tiempo de Reacción:**

La comunicación eficaz entre tu sistema sensorial y sistema motor te permite protegerte de situaciones perjudiciales. Cuando alguien te tira un bola, tu primer instinto es el de subir tus manos para proteger tu cuerpo. Si tocas algo bien caliente no tienes que pensar dos veces para retirar tu mano. Estos y otros reflejos musculares dependen de la comunicación rápida entre las neuronas del sistema sensorial y las neuronas del sistema muscular. La velocidad de esta comunicación depende en parte de la distancia que la señal tiene que recorrer.

En este experimento vamos a estudiar la coordinación entre el sistema sensorial y el sistema motor. Vas a medir el tiempo de reacción a un estímulo en particular, en este caso la caída de una regla. Cuando hagas este ejercicio ten en cuenta lo importante que es el tener respuestas rápidas a los estímulos que enfrentas cada día.

**MATERIALES:**

- Regla

**METODO:**

Haz que tu compañer@ sostenga una regla. Mantén tus dedos índice y pulgar abiertos en la parte de debajo de la regla (cerca de la marca de 0 pulgadas). Tu compañer@ dejara caer la regla al azar 3 veces y tú tienes que agarrar la regla lo más pronto posible (Nota: Tu compañer@ no debe decirte cuando va a dejar caer la regla). Apunta la marca en pulgadas donde agarraste la regla cada vez. Convierte las pulgadas a segundos con la tabla.

**HIPÓTESIS:**

**OBSERVACIONES:**

| **TIEMPO DE REACCIÓN (PULGADAS)** | | |
| --- | --- | --- |
| **PRUEBA #1** | **PRUEBA #2** | **PRUEBA #3** |

| Distancia (pulgadas) | Tiempo (segundos) |
| --- | --- |
| **2** | 0.10 (100 ms) |
| **4** | 0.14 (140 ms) |
| **6** | 0.17 (170 ms) |
| **8** | 0.20 (200 ms) |
| **10** | 0.23 (230 ms) |
| **12** | 0.25 (250 ms) |
| **17** | 0.30 (300 ms) |
| **24** | 0.35 (350 ms) |
| **31** | 0.40 (400 ms) |
| **39** | 0.45 (450 ms) |
| **48** | 0.50 (500 ms) |
| **69** | 0.60 (600 ms) |

| **TIEMPO DE REACCIÓN (SEGUNDOS)** | | | |
| --- | --- | --- | --- |
| **PRUEBA #1** | **PRUEBA #2** | **PRUEBA #3** | **PROMEDIO** |
|  |  |  |  |

**PREGUNTAS:**

1. Cuales otros factores y/o condiciones crees que podrían afectar el tiempo de reacción?

**CONCLUSIÓN:**

**Sistema Nervioso Autonómico**

**EXPERIMENTO #5: Presión Sanguínea**

El **Sistema Nervioso Autonómico** (SNA) es la división del Sistema Nervioso que es responsable por el control del movimiento de tus intestinos, ritmo cardiaco, y glándulas. Existen dos divisiones del SNA: el **sistema nervioso simpático** y **parasimpático** (Fig 16). Para la mayoría de las funciones corporales, estas dos divisiones trabajan en direcciones opuestas para controlar exactamente la función de tus órganos. Por ejemplo, el sistema nervioso simpático aumenta el ritmo cardiaco, mientras que el sistema nervioso parasimpático disminuye el ritmo cardiaco.

En este ejercicio vamos a explorar como factores externos, como drogas o ejercicios, pueden afectar la función de las dos divisiones del SNA; usaremos medidas de presión sanguínea para determinar esto. Has aprendido en las pasadas lecturas como desarrollar y probar hipótesis. ¡Ahora tienes la oportunidad de formular y probar tu hipótesis! ¿Crees que la cafeína activa el sistema nervioso simpático o parasimpático? ¿Cómo lo comprobarías? ¿Y que tal el ejercicio? ¿Estrés? ¿Algún otro factor?

**Figura 16. Sistema Nervioso Autonómico.**

**MATERIALES:**

- Manga para medir presión sanguínea
- Bebidas con cafeína
- Cronógrafo
- Escaleras
- Sé creativo/a…

**MÉTODO:**

Usando los materiales que tienes disponible o cualquier otro material que se te ocurra, formula una pregunta de investigación que puedas probar. También formula y prueba una hipótesis acerca de cómo un factor externo (drogas (cafeína), ejercicio, estrés) afecta el balance entre las divisiones simpáticas y parasimpáticas del SNA.

**HIPÓTESIS:**

**OBSERVACIONES:**

**CONCLUSIONES:**
