# Supplementary material for: Bridge to neuroscience workshop: An effective educational tool to introduce principles of neuroscience to Hispanics students
Source: PLoS One. 2019 Dec 12;14(12):e0225116. doi: 10.1371/journal.pone.0225116 (PMC6907774; doi:10.1371/journal.pone.0225116)
Supplement: S3 File — (DOCX) [file pone.0225116.s004.docx]

**NEUROLOGICAL DISEASES**

**Pathophysiology of Parkinson disease (PD):**

PD results from the loss of pigmented cells in the substantia nigra (SN), an area of the brain known to contain dopaminergic neurons. These neurons project to the basal ganglia, where they coordinate and modulate voluntary movement. Additionally, protein aggregations within neuronal cell bodies, called Lewy Bodies, are observed throughout the brain.

**Pathophysiology of Alzheimer’s disease (AD):**

The causes and mechanisms of the brain abnormalities underlying AD are not yet fully understood. There are two characteristic pathophysiological features: 1) the loss of neurons and synapses in the cerebral cortex and hippocampus, which results in extreme shrinkage, or *atrophy*, of the brain; and 2) the abnormal accumulation of beta-amyloid plaques and neurofibrillary tangles. The small fibrillary peptide, *beta amyloid*, aggregates in the spaces around synapses (*neuritic plaques*) and a modified form of the protein tau accumulates in the cell bodies of neurons (*neurofibrillary tangles*). In all forms of AD plaques and tangles mostly develop in brain regions important for memory and intellectual functions, including the hippocampus, basal forebrain, and cerebral cortex.

**Pathophysiology of Amyotrophic Lateral Sclerosis (ALS):**

ALS is a type of motor neuron disease characterized by the gradual degeneration and death of motor neurons. Upper motor neurons in the brain transmit signals to lower motor neurons in the brainstem and spinal cord, which in turn send the message on to particular muscles. In ALS, both upper and lower motor neurons degenerate.

ALS is present in two forms: familial ALS (FALS) and sporadic ALS (SALS). However, whether a patient obtained the disease sporadically or by familial origin is indistinguishable. Familial ALS forms 10% of ALS cases, and 20% of these patients have a mutation in the Cu^2+^/ Zn^2+^ superoxide dismutase 1 (SOD1). Sporadic ALS forms 90% of the ALS cases and 12% of these patients also show the SOD1 mutation. Studies have shown that FALS and SALS have the same pathology, and it is hoped that therapies effective in SOD1 models can translate and be useful to treat to both familial and sporadic ALS.

**Pathophysiology of Cerebrovascular disease (stroke):**

A stroke occurs when either a blood vessel bringing oxygen and nutrients to the brain bursts or is clogged by a blood clot or some other particle. This deprives the brain of blood, causing the death of neurons within minutes. The initial insult triggers a cascade of cell death events, including oxidative stress and inflammation, which can increase the infarct size (area of tissue death) and lead to permanent brain damage. For these reasons, time is of the essence! The faster a patient received medical treatment, the less damage is likely to occur.

**Figure 4**. Pathophysiology of stroke.

**CASE STUDIES**

http://memphisvascular.com/patient-education/stroke/

**Case Study #1:**

A 65 year-old man presented with a resting tremor in both hands. The tremor began approximately 10 years earlier in his right hand and had become increasingly more pronounced. He also described a “stiff, slow” feeling throughout his body, which prevented him from performing rapid coordinated movements. His posture was stooped and there was tendency to shuffle when he walked. Examination revealed that movements were difficult to initiate and performed slowly (bradykinesia).

Over the course of five years, these motor symptoms became progressively worse, affecting the patient’s ability to perform everyday tasks, such as dialing a telephone number or walking from the kitchen to the living room unaided. Treatment was able to relieve symptoms and slow the progression of the disease; however the dose of the medication had to be increased gradually. Ultimately the treatment itself began to impair quality of life. In the final stages of the disease, the patient suffered not only from severe motor symptoms, but also cognitive and behavior symptoms, including dementia and depression. The man survived into his 79^th^ year, passing away from an infection of pneumonia.

Can you identify this disorder?

**Case Study #2:**

The patient was a 72-year-old woman who was moderately overweight (BMI of 28) and had been smoking one pack of cigarettes a day for the past 40 years. She awoke one morning with weakness on the right side of her body. She was moderately confused, seeing double, and slurring her speech. When she attempted to walk to the bathroom, she stumbled and fell twice. Her daughter immediately called 911. The patient was unresponsive when emergency personnel arrived 20 minutes later, but slowly regained consciousness over the following two days. Once fully alert, she was experiencing paralysis and loss of sensation on the right side of her face and arm and an inability to speak but was responsive to verbal commands (e.g. blink once for yes, twice for no).

The patient was monitored over the following five years. Physical therapy helped to improve the symptoms of right hemi paralysis dramatically; the patient was able to use her arm and hand for tasks such as buttoning a shirt, drinking water from a cup, and writing. Additionally, the patient regained partial sensation in the affected areas. Her ability to speak was also recovered; however her speech was slightly slurred and slowed.

Can you identify this disorder?

**Case Study #3:**

The patient was a 53-year-old male who presented with the complaint of a left foot drop that was affecting his ability to walk normally. Additionally he reported muscle twitching and cramping in both lower limbs. Examination revealed mild weakness in all extremities, and mild difficult with speech (dysarthria) and swallowing (dysphagia).

The patient was monitored over the course of the next three years. During this time, motor symptoms became more pronounced and severe weight loss was noted due to muscle atrophy. Eventually the patient was unable to stand or walk, he had complete loss of the ability to use his hands and arms, and chewing and swallowing was increasingly difficult. Cognitive function remained intact, however because of the progressive motor loss, the patient became anxious and depressed. Respiration became increasingly labored, as the diaphragm and intercostal muscles (rib cage) weakened. These symptoms were treated for a short period of time, however the disease progression soon overcome all respiratory treatment options. The patient elected to enter hospice care and died in his 55^th^ year of respiratory failure.

Can you identify this disorder?

**Case Study #4:**

The patient was a 72 year old woman with a one year history of confusion, apathy, and insomnia. Six months prior to the initial visit she had moved from California to Michigan and had great difficulty learning to navigate in her new surroundings. She had a history of depression, but no other serious neurological diseases. Examination noted intact attention, intact reading and writing, and intact long-term memory. Mood stabilizers were prescribed to treat her depressive mood.

She was seen occasionally over the next few years to follow-up on her depression. Notes from a visit in her 75^th^ year said that her daughter was having trouble getting her dressed and out of the house. She had decreased energy and outbursts of aggression. The neurological examination noted her thoughts wandered easily from topic to topic and her reading and writing skills were diminished. She was hospitalized at the age of 78 for dementia and paranoia. She moved to a care facility at 79 and died within a year due to an infection arising from a pressure ulcer.

Can you identify this disorder?
