# Supplementary material for: Bridge to neuroscience workshop: An effective educational tool to introduce principles of neuroscience to Hispanics students
Source: PLoS One. 2019 Dec 12;14(12):e0225116. doi: 10.1371/journal.pone.0225116 (PMC6907774; doi:10.1371/journal.pone.0225116)
Supplement: S4 File — (DOCX) [file pone.0225116.s005.docx]

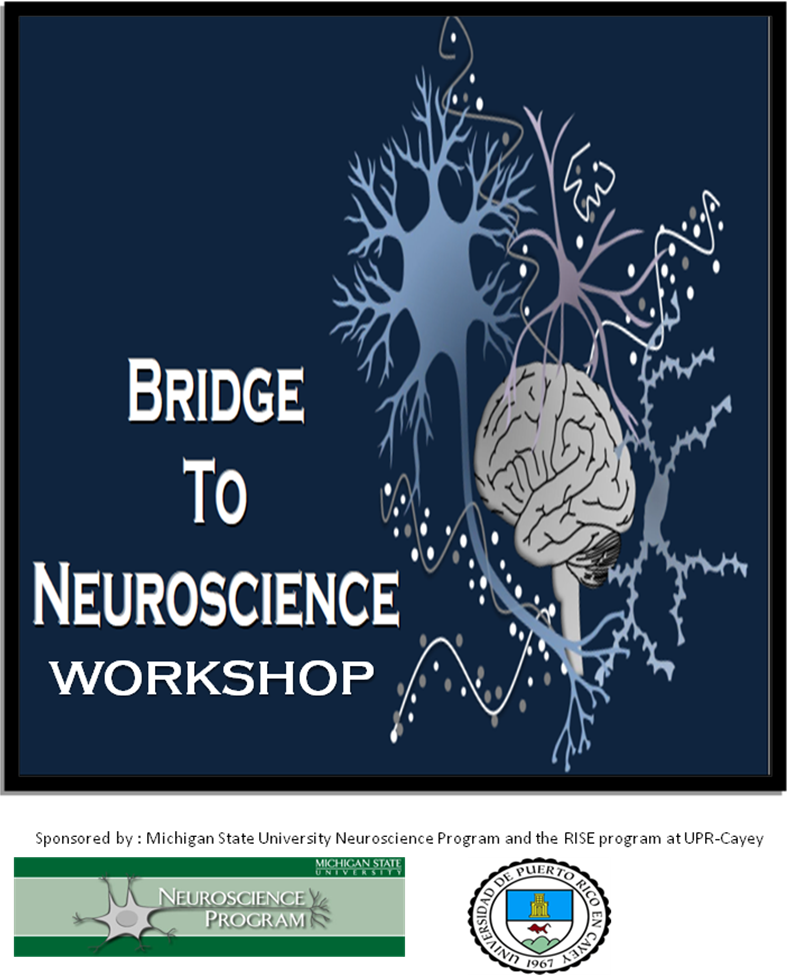


Sponsored by: Michigan State University Neuroscience Program.


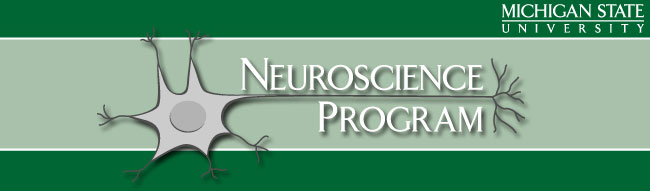


To find pictures of today’s workshop and for more information go to:

**https://www.msubpnp.com/workshops**

**Bridge to Neuroscience Workshop-Book**

**Contributors**

Alexandra Colón-Rodríguez, PhD

Eileen S. Rodriguez Tapia, PhD

Chelsea Tiernan, PhD

Brenda Marrero-Rosado, PhD

Chelsea Hutch, PhD

Carla Dams, MS

**Editors**

Alexandra Colón-Rodríguez, PhD

Chelsea Tiernan, PhD

**BPNP Director**

William D. Atchison, PhD

Michigan State University

1355 Bogue Street

B307 Life Science Bldg.

East Lansing, MI 48824

atchiso1@msu.edu

**THE NERVOUS SYSTEM**

Think about your favorite sports team or a symphony orchestra. What do they have in common? Both are composed of a group of people under the direction of one leader- a coach in the case of a sports team and a conductor in the case of the orchestra. This leader aims to influence and coordinate the behavior of the group. In addition, this person also receives and integrates information from every member of the group to produce the desired outcome (i.e. winning a championship or performing a delightful concert).

The nervous system (NS) can be seen as the coach or conductor of our body. It receives information from all parts of the body, and then integrates that information to influence and coordinate the functions of the many different components of our body. As a result, our body functions at an optimal level, and we are able to navigate and interact with the external environment surrounding our body.

**Divisions of the nervous system**


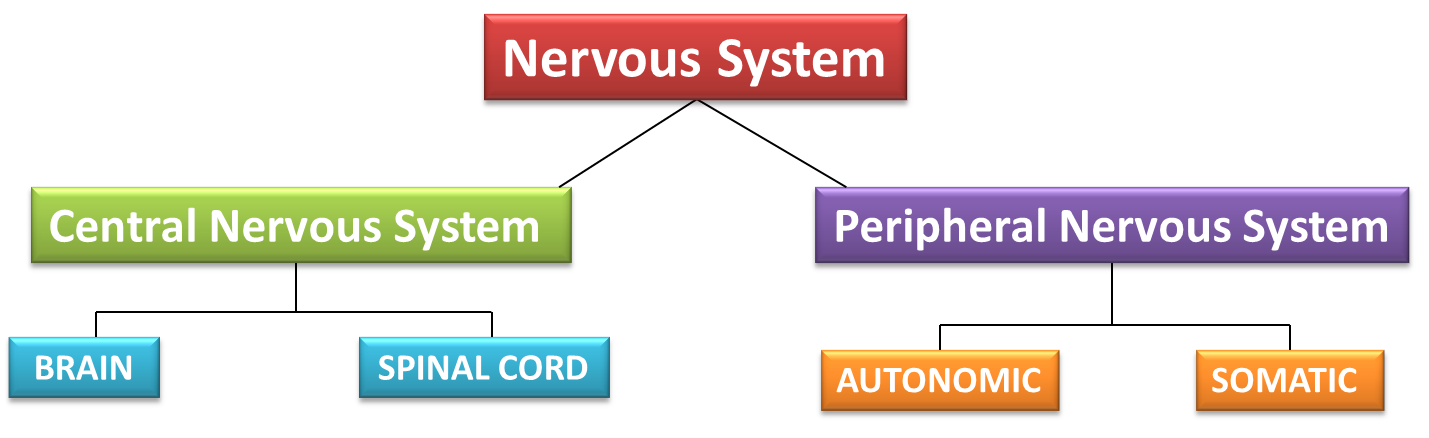
 The nervous system has two main divisions: the **central nervous system** and the **peripheral nervous system** (Fig 1). The central nervous system is composed of the **brain** and the **spinal cord**. The peripheral nervous system is composed of the nerves that innervate our organs to carry sensory and motor information to and from the central nervous system. The peripheral nervous system is further subdivided in two divisions, the **autonomic nervous system** and the **somatic nervous system**.

**Figure 1. Divisions of the nervous system.**

Central nervous system

The central nervous system (CNS) is known as central because it receives information from all parts of the body and uses it to modulate the behaviors and functions of the body, in turn. The CNS is composed of the brain and spinal cord.

*Central nervous system: Brain*

The **brain** is the control center of the body (Fig 2). On average the human brain weighs 3 pounds. The brain is composed of two **cerebral hemispheres** known as the left and right hemispheres. The surface of each cerebral hemisphere consists of “grooves” known as **sulci** and “bumps” known as **gyri**. These structures allow a greater amount of cerebral cortex to be contained inside the skull. This is important because as the area of the cerebral cortex increases, the capacity of the brain to receive and process information increases as well. The cerebrum is divided in four lobes: **frontal**, **parietal**, **temporal**, and **occipital**. Each lobe is associated with a particular set of functions that enable us to perceive and interact with our surrounding environment.


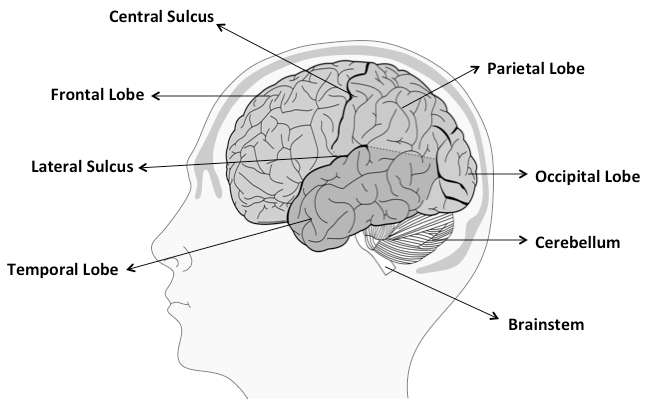
 The **frontal lobe** is located in the anterior (front) part of the brain. It processes information related to planning, reasoning, problem-solving, emotional expression, behavioral control, judging, and movement. Behind the frontal lobe is the **parietal lobe***,* this part is mainly responsible for the perceptions of stimuli coming from our skin such as touch, temperature, pain, and pressure. The **central sulcus**, a very prominent landmark in the cerebrum, separates these two lobes. The **temporal lobe** is located below the frontal and parietal lobes, and is responsible of processing auditory information and memory. Another prominent landmark in the cerebrum is the **lateral sulcus**, which separates the temporal lobe from the frontal and parietal lobes above. Lastly, the **occipital lobe,** located in the posterior (back) region of the brain, is responsible for processing visual information.

**Figure 2. Structures of the brain.**

**Figure 2. Structure of the brain**


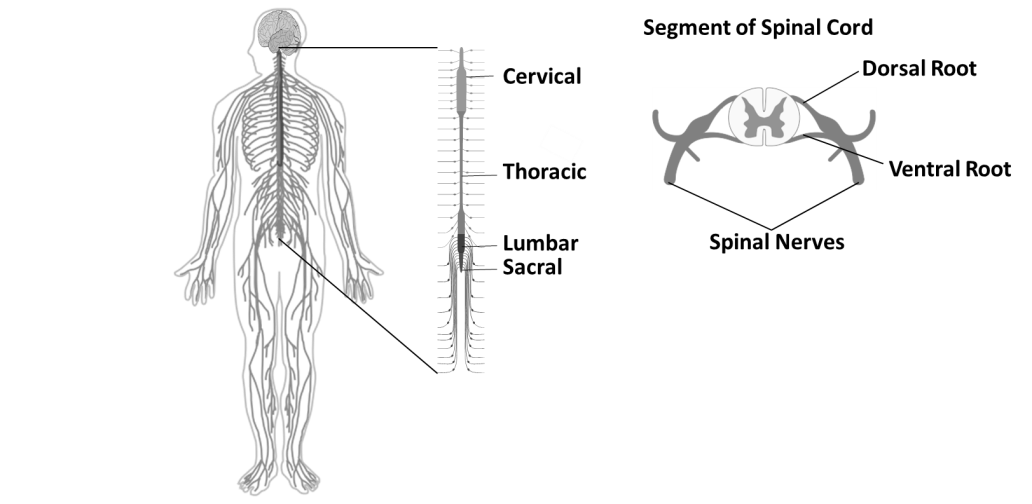
 The **cerebellum** (Latin for “little brain”) is located in the posterior region, below the cerebral hemispheres. This brain structure is responsible for coordination, precision, and fine motor movements. However, it does not initiate motor movements. The **brainstem** is located at the base of the brain, and is continuous with the spinal cord below. The brainstem includes three structures, the midbrain, pons, and medulla oblongata, which control breathing, heart rate, arousal and consciousness, and sleep-wake cycles.

**B**

**B**

**A**

*Central nervous system: Spinal cord*

The **spinal cord** is the main “highway” connecting the brain to the peripheral nervous system, and thus is a major pathway for information exchange between these two parts of the NS. The spinal cord is 40 to 50 cm long and 1 to 1.5 cm in diameter. In humans, the spinal cord is protected by a bony structure called the vertebral column. Bones that make up the vertebral column are called vertebrae. There are a total of 31 segments: 8 cervical, 12 thoracic, 5 lumbar, 5 sacral and 1 coccygeal (Fig 3A). A pair of spinal nerves emanate from the right and left side of each segment making a total of 31 pairs of spinal nerves. Each spinal nerve is composed of two roots: a ventral and a dorsal root (Fig 3B). Nerves within the ventral root convey motor information to skeletal muscles, whereas nerves within the dorsal root convey sensory information to the spinal cord and brain.

**Figure 3. Structure of the spinal cord. A) Divisions of the vertebral column. B) Cross-section of the spinal cord.**

Peripheral nervous system

The peripheral nervous system (PNS) is composed of nerves that reside outside the brain and spinal cord. The PNS is not protected by a bony column, and thus it is more vulnerable to damage following accidents, injuries, or illness. The PNS is divided in the autonomic and somatic divisions.

*Peripheral nervous system: autonomic nervous system*

The **autonomic nervous system** modulates the functions of internal organs, such as the heart, stomach, intestine, and glands. Two subdivisions of the ANS work in opposition to one another to control the activity of these organs in response to two different types of situations. The **sympathetic division of the ANS** stimulates organs of the body in response to stressful, emergency situations. This is often referred to as the “fight or flight” response. The **parasympathetic division of the ANS** relaxes the body in response to non-emergency situations, and is sometimes called the “rest and digest” response. The neurons in each division of the ANS communicate with the same muscles and glands; however, their actions within those muscles and glands are opposite. For example, if you see a shark while swimming in the ocean, the sympathetic division will increase your blood pressure, your heart will beat faster, and the digestion of food in your stomach will slow down. These actions enable you to cope with presence of the shark. Once you have safely (and quickly!) returned to shore, the parasympathetic division reverses the actions of the sympathetic division. Your blood pressure and heart rate will slow down, your breathing will return to a normal rate, and you will resume digestion.

The autonomic nervous system includes a third subdivision called the **enteric nervous system** (ENS). The ENS is also known as the “little brain in your gut” because all the neurons that control the main functions of the digestive system reside within the walls of the gastrointestinal tract, extending from the esophagus to the anus. The ENS contains 100 million neurons, as many neurons as there are in the spinal cord. They work together to control important functions of digestion, such as consecutive contraction and relaxation movements that propel contents along the gastrointestinal tract, absorption of nutrients, and secretion of hormones.

*Peripheral nervous system: somatic nervous system*

The second division of the PNS is called the **somatic nervous system**. Contrary to the autonomic nervous system the somatic nervous system regulates the control of voluntary body movements mediated by skeletal muscles. This system includes nerves that relay sensory information towards the CNS (the spinal cord and the brain). These nerves relaying information towards the CNS are called **afferents**. The somatic nervous system also includes nerves that initiate the contractions of skeletal muscles. These nerves that communicate to the muscle or convey motor information away from the CNS are called **efferent**.

To better understand how the nervous system works you should be familiar with the basic unit of this system: the neuron. In the next section, you will find information about the unique structure and function of the neuron. You will also learn about all of the key features that allow neurons to work together to make the nervous system work!

**THE NEURON**


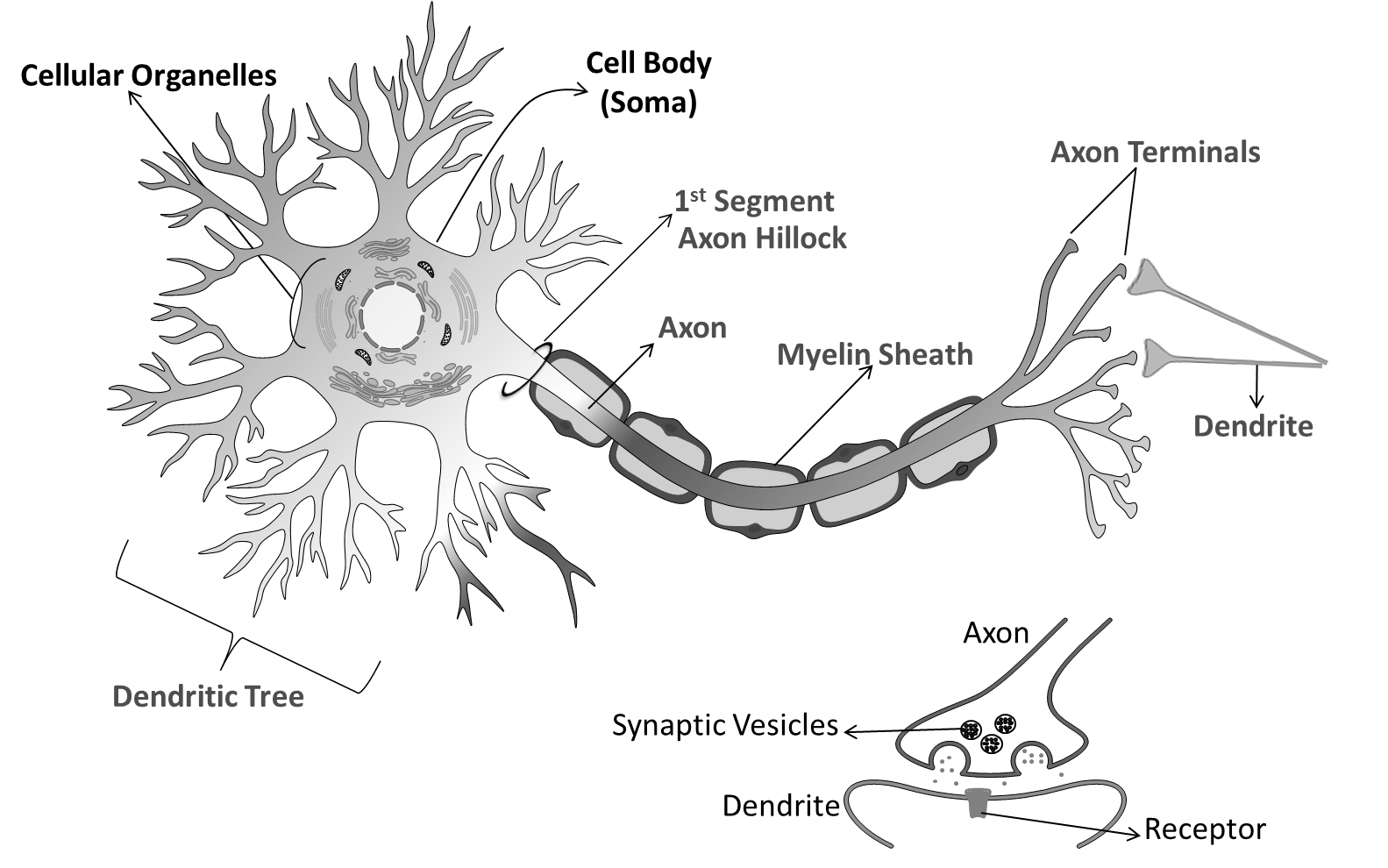


**Figure 1**

**Figure 4. Structure of a neuron (motor neuron).**

The neuron is the basic structural and functional unit of the nervous system. It performs the most important function of the nervous system, transmitting information both within (intracellular) and between (intercellular) cells. Neurons are able to carry out this essential task of communication because of a collection of specialized structural elements (Fig 4):

1. The **soma**, also called the **cell body**, is the control center of the neuron. It receives electrical signals from the dendrites, and conducts electrical signals to the axon. The soma also contains many cellular organelles found in non-neuronal cells, including the nucleus, mitochondria, rough and smooth endoplasmic reticulum, Golgi apparatus, ribosome and others required for protein synthesis and other metabolic processes.
2. **Dendrites** are short processes that arise from and branch off the soma. Dendrites are considered to be input centers because they receive communication from adjacent neurons, and then transfer this received information to the soma. It is the expression of specific neurotransmitter receptors that enables dendrites to receive input from other neurons.
3. The **axon** is a single process that originates from the soma to make contact with other neurons, muscles cells, or glands. It is typically longer than a dendrite, however its length varies from a few micrometers to a few meters, depending on the type of neuron type and the organism in which it resides. The axon is considered to be the transmission center because it propagates electrical signals called action potential from the cell body to the axon terminal. The first part of the neuron is a triangular region called the axon hillock. This is where action potentials are generated. At its distal end, the axon usually branches extensively to make contact with multiple target cells. The extreme tips of each axon branch are called the **axon terminal** (D) (i.e. synaptic terminal, nerve terminal). This region of the axon contain synaptic vesicles packed with neurotransmitters and the cellular machinery required for synthesis, storage, release and re-uptake of neurotransmitters at the synapse (described below).

**Axonal Transport**

In neurons, the synthesis of protein occurs almost exclusively in the soma and proximal dendrites, including those proteins that are needed in other cellular compartments such as the nerve terminal. **Axonal transport** is a mechanism by which material can be carried between the cell body and the nerve terminal. The process of axonal transport is **bi-directional**: anterograde transport moves proteins from the cell body to nerve terminal, whereas retrograde transport moves proteins from the nerve terminal to the cell body. Retrograde transport serves to either degrade or re-use material or to deliver signals to the cell body.

**There are two types** of axonal transport (Fig 5). Large membranous organelles (secretory vesicles, synaptic vesicle precursor membranes, large dense-core vesicle containing neuropeptides, mitochondria, etc.) are transported by **fast axonal transport.** Organelles are attached to *microtubules* and shuttled to their designation by motor proteins, kinesin for anterograde and dynein for retrograde transport. The process of fast axonal transport is similar to a train moving along its track, where microtubules represent the train tracks, the motor protein represents a train engine, and the organelle being transported along the axon is the cargo held with a train car pulled by the engine. **Slow axonal transport** results from the gradual flow of fluid within the axon, called *axoplasm*, and does not rely on microtubules or motor proteins. Cytosolic proteins (enzymes responsible to synthesize small molecule neurotransmitters) and cytoskeletal elements (neurofilaments, microtubules) are transported by **slow axonal transport**.


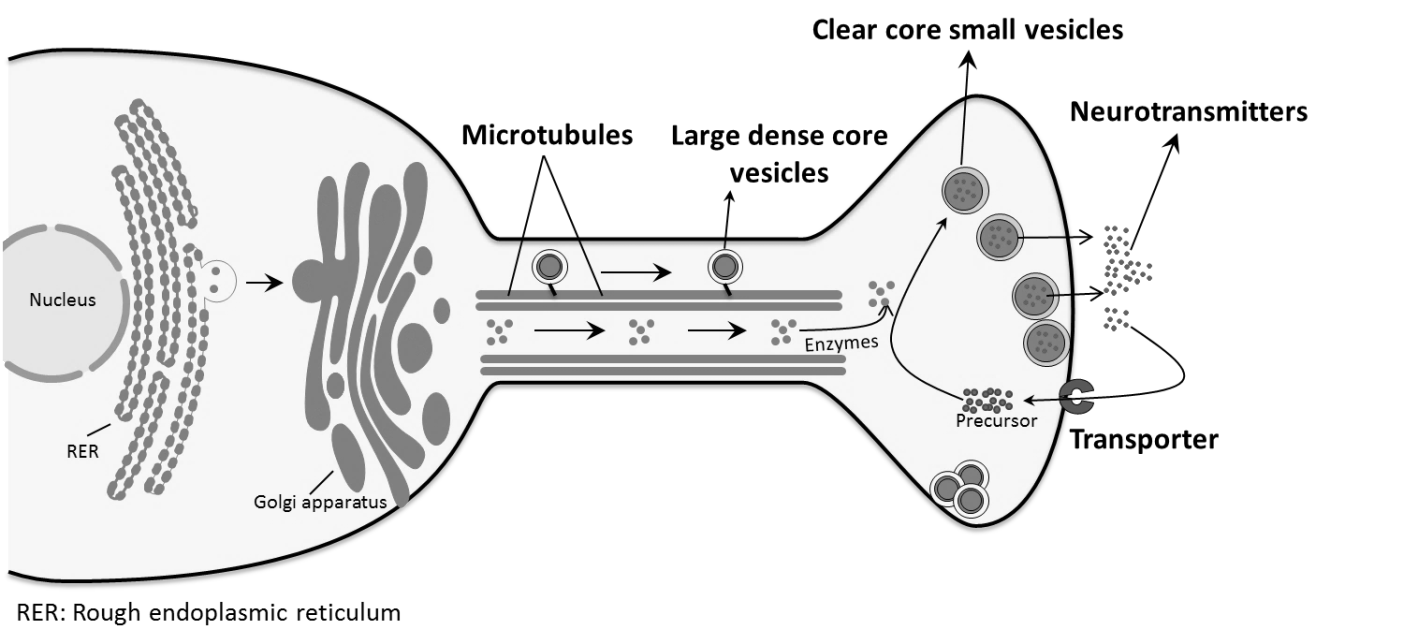


**Figure 5. Axonal transport.**

**Resting membrane potential**

Neurons can generate and propagate electrical signals, which is dependent on the movement of ions (charged particles) across the cell membrane. All cells have a negative membrane potential (*Vm*) which arises from the uneven distribution of ions across the cell membrane. The *Vm* in a neuron at rest is between -40 mV to -90 mV (Fig 6). This *Vm* is called the **resting membrane potential.** Potassium (K^+^) is the predominant cation (positively charged ion) in the intracellular environment. Sodium (Na^+^) is the predominant cation in the extracellular environment. Chloride (Cl^-^) is the predominant anion (negatively charged ion) in the extracellular environment, and impermeable anions (proteins, nucleic acids, lipids) are sequestered to the interior of the cell. This unequal distribution of ions creates a **concentration gradient.** For example, K^+^ is more highly concentrated inside the cell, and Na^+^ is more concentrated outside the cell. Because ions have a charge, the unequal distribution of ions across the cell membrane also creates an **electrical gradient**. There are more negatively charged ions inside of the cells, and therefore the intracellular environment has a negative charge relative to the extracellular space. Together the concentration gradient and electrical gradient are referred to as the **electrochemical gradient**.

**Factors that contribute to the electrochemical gradient**

The Na^+^/K^+^ ATPase pumps Na^+^ out and K^+^ into the cell (against their concentration gradients) maintaining the steep concentration gradients for these ions. This requires active transport utilizing energy released from ATP hydrolysis. The Cl^-^ pump works by pumping chloride ions out of the cell to maintain the steep concentration gradient for Cl^-^. One final important factor in the electrochemical gradient is the permeability of the cell membrane. The cell membrane has pores formed by transmembrane proteins, which enable ions to cross the membrane. These pores are called *ion channels*. The K^+^ “leak channels” are always open, and as a result, when the neuron is at rest, K^+^ will move down its concentration gradient and flow from the intracellular to extracellular space. Complete loss of K^+^ inside the cell is prevented by electrostatic interaction with impermeable anions inside the cell. This will leave an excess of negative charges inside the cell compared to the outside making the resting *Vm* negative.

**Figure 6. The distribution of ions across the plasma membrane of a neuron.**


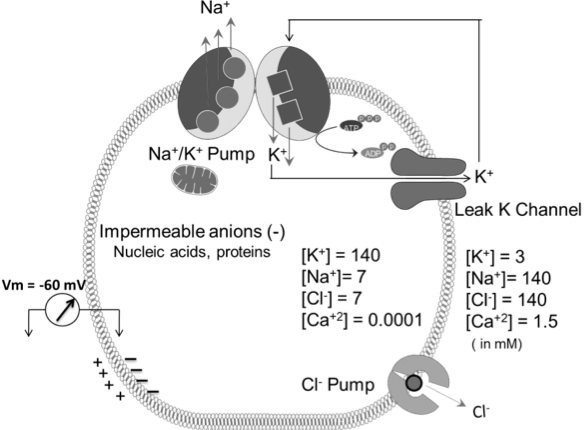


The *Vm* of a neuron changes in response to synaptic input from other neurons. **Depolarization** occurs when the *Vm* becomes more positive than resting Vm. **Hyperpolarization** occurs when the *Vm* becomes more negative than the resting *Vm*.

**Action potential**

Active membrane potential or the generation of an action potential requires an initial depolarization of the resting *Vm*. This can be produced by the action of some neurotransmitters, injection of current into the neuron or entry of Na^+^ ions through Na^+^ channels. Two channels are involved in the generation of an action potential: *voltage-gated Na^+^* and *voltage-gated K^+^ channels* (Fig 7). The proteins forming these channels are sensitive to the voltage across the membrane. When *Vm* is depolarized these voltage-sensitive channels change their shape, and open a gate that allows either Na^+^ or K^+^ (depending on the type of channel) to flow down their concentration gradient. Voltage-gated Na^+^ channel will open when the *Vm* is depolarized to -50 mV. Voltage-gated K^+^ channel will open when the *Vm* is depolarized to 0 mV.


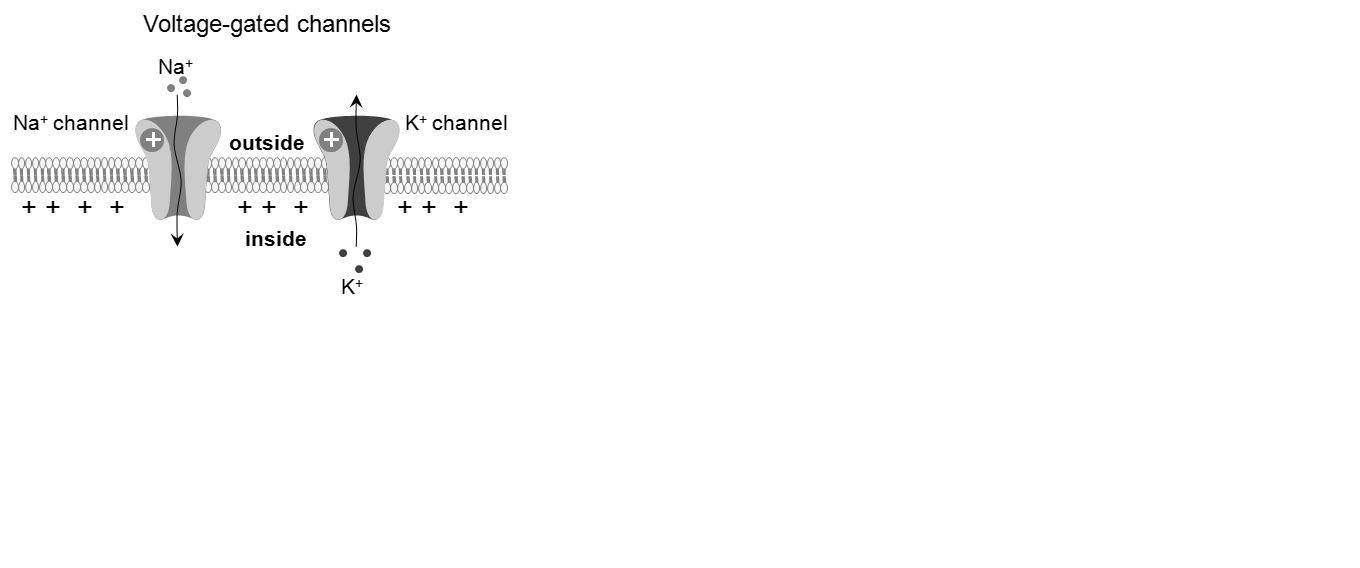


**Figure 7. Ion channels involved in the generation of an action potential.**


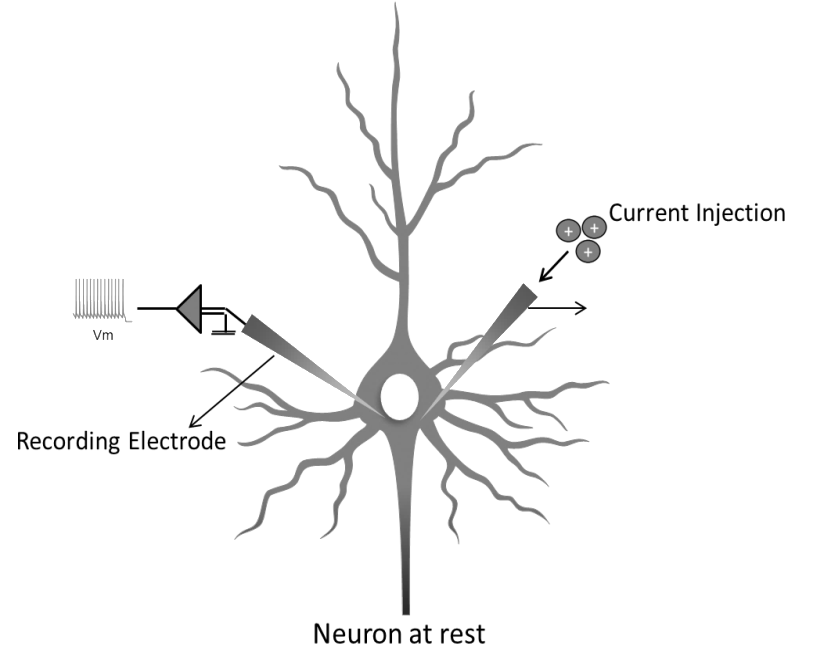
 We can record *Vm* and stimulate a neuron to generate an action potential using glass microelectrodes (ME; Fig 8). When a recording ME is inserted into the cytosol, close to the axon hillock of a neuron and connected to an amplifier, the *Vm* can be recorded. If the neuron is at rest the *Vm* will be close to -65 mV. If a stimulating ME is also inserted close to the recording ME and positive current is injected, the *Vm* will depolarize. When the *Vm* reaches **-50 mV**, an action potential will be generated **(that is the threshold for action potential generation)**. When the magnitude of the injected current is increased more action potentials will be generated (higher frequency; Fig 9).

Neuron at rest

**Figure 8. Example of an electrophysiological recording.**


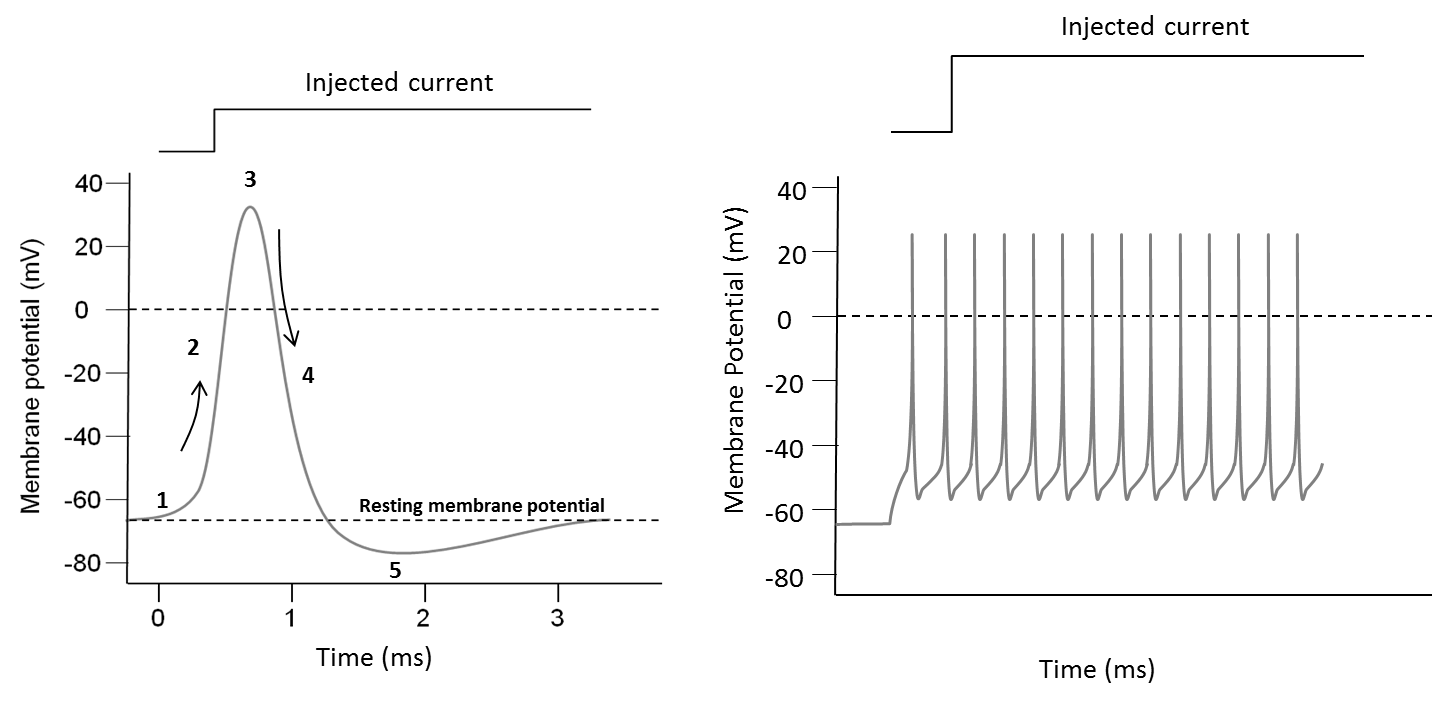


**Figure 9. Generation of an action potential by current stimulation.**

**Figure 9. Generation of an action potential by current stimulation**

Ionic basis of an action potential

When a stimulus depolarizes the *Vm* to -50 mV, the voltage-gated Na^+^ channel will open and Na^+^ will rush into the neuron further depolarizing the neuron and producing the ***rising phase*** ***(2)*** of the action potential. Na^+^ ions continue to enter the neuron until the concentration of Na^+^ ions reaches equilibrium across the membrane (~ at 40 mV), which corresponds to the ***overshoot (3)*** phase of the action potential. At this time, voltage-gated K^+^ channels open, K^+^ ions rush into the extracellular space, which produces the ***falling phase (4)*** of the action potential and returns the *Vm* to a negative potential. Because the membrane is even more permeable to K^+^ (there are more K^+^ channels open: leak and voltage-gated channel), an ***undershoot phase (5)*** is generated, in which the *Vm* becomes more negative than the resting *Vm*. The hyperpolarization of the *Vm* during the undershoot phase closes voltage-gated K^+^ channels and de-activate voltage-gated Na^+^ channels. Additionally, Na^+^/K^+^ ATPase will restore the resting electrochemical gradient in preparation for another action potential.

Propagation of an action potential: myelinated vs. unmyelinated axons

The rate at which an action potential is conducted limits the flow of information throughout the nervous system. Action potential propagation requires both active and passive current (Fig 7 and 8). Depolarization opens Na^+^ channels initiating an action potential. The resulting inward current diffuses passively along the axon depolarizing an adjacent region, opening Na^+^ channels, and generating an action potential in this new location. The farther the diffusion of the passive current, the faster the propagation of the action potential. The extent of diffusion of passive current is limited by how much passive current leak out through the membrane.


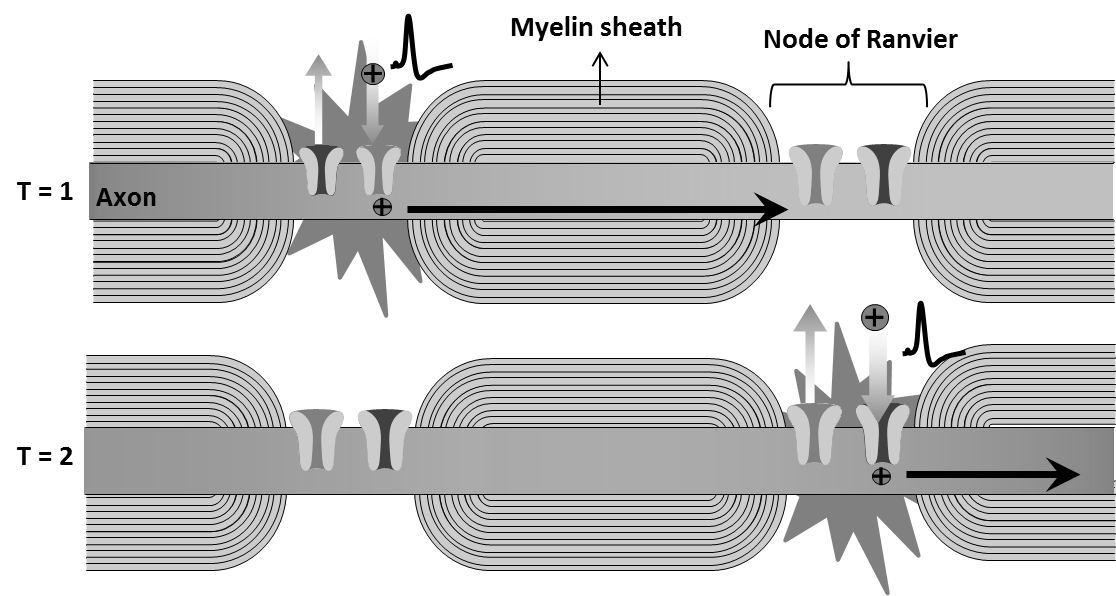


**Figure 10. Propagation of an action potential in a myelinated axon.**

Insulation of the axonal membrane is a strategy to improve the passive flow of electrical current along the axon since it will decrease the amount of current that leaks out through the membrane. Myelination (multiple layers of closely oppose glial membranes) of the axon act as an electrical insulator and greatly speeds up action potential conduction. Unmyelinated axons have a conduction velocity of 0.5 - 10 m/s, whereas myelinated axons can conduct at a velocity up to 150 m/s.

In myelinated axons, action potential generation occurs only at specific sites along the axon, called **nodes of Ranvier** (Fig 10)***.*** At this point there is a gap in the myelin sheath. In contrast unmyelinated axons generate action potentials at multiple points and in the context of neurons this is a “time-consuming process” (Fig 11).

The passive current elicited after the occurrence of an action potential in one node will travel through the myelinated segment of the axon until reaching the next node to generate another action potential at that point. The cycle is repeated until the action potential arrives at the nerve terminal. This type of propagation is called **saltatory conduction** because the action potential *“jumps”* from node to node.


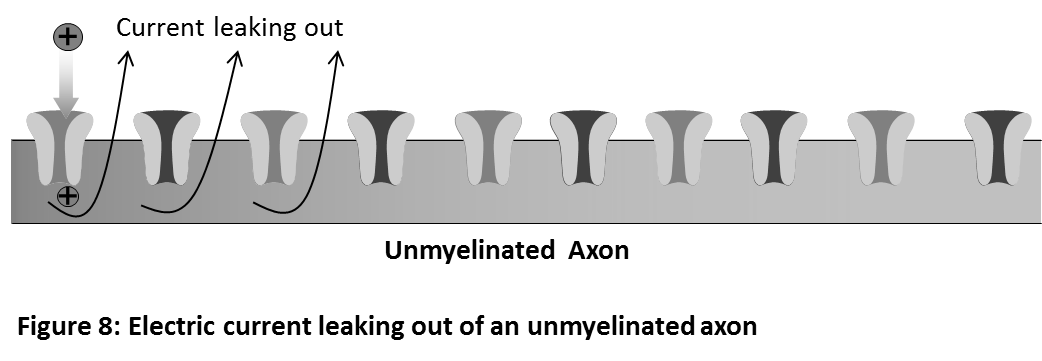


**Figure 11. Electric current leaking out of an unmyelinated axon.**

**The chemical synapse**

In order for a neuron to transmit information between two cells, or *intercellular*, the action potential must be converted into a chemical signal that can bridge the gap between the axon terminal and its target cell. This gap is called the **synaptic cleft** (i.e. synaptic gap). When the action potential arrives at the axon terminal it will induce the release of **neurotransmitters** from **synaptic vesicles** in the axon terminal that diffuse across the synaptic cleft to bind receptors on the surface of the target cell.

Characteristics of the chemical synapse:

1. Synapses have pre-synaptic and post-synaptic membranes that are separated by a **synaptic cleft** that is 20-50 nm wide.
2. The synaptic cleft is filled with a matrix of fibrous extracellular proteins that help maintain adhesion between the pre- and post-synaptic membranes.
3. Pre-synaptic component is usually an axon terminal containing **synaptic vesicles**: small clear-core vesicles (50 nm diameter) that contain small neurotransmitter molecules and large dark-core vesicles (100 nm diameter) that contain large peptides called neuropeptides.
4. **Active zones** are protein accumulations in the pre-synaptic membrane that is exactly opposed to the post-synaptic membrane density. This is the actual site of neurotransmitter release and the proteins expressed at this zone are all involved in this process.
5. **Post**-**synaptic densities** are protein accumulations along the surface of the post-synaptic target that contain neurotransmitter receptors responsible for converting the intercellular signal into an intracellular signal within the post-synaptic cell.
6. The nature of the post-synaptic responses can vary depending on the type of neurotransmitter being released and the type of receptor that is being activated.


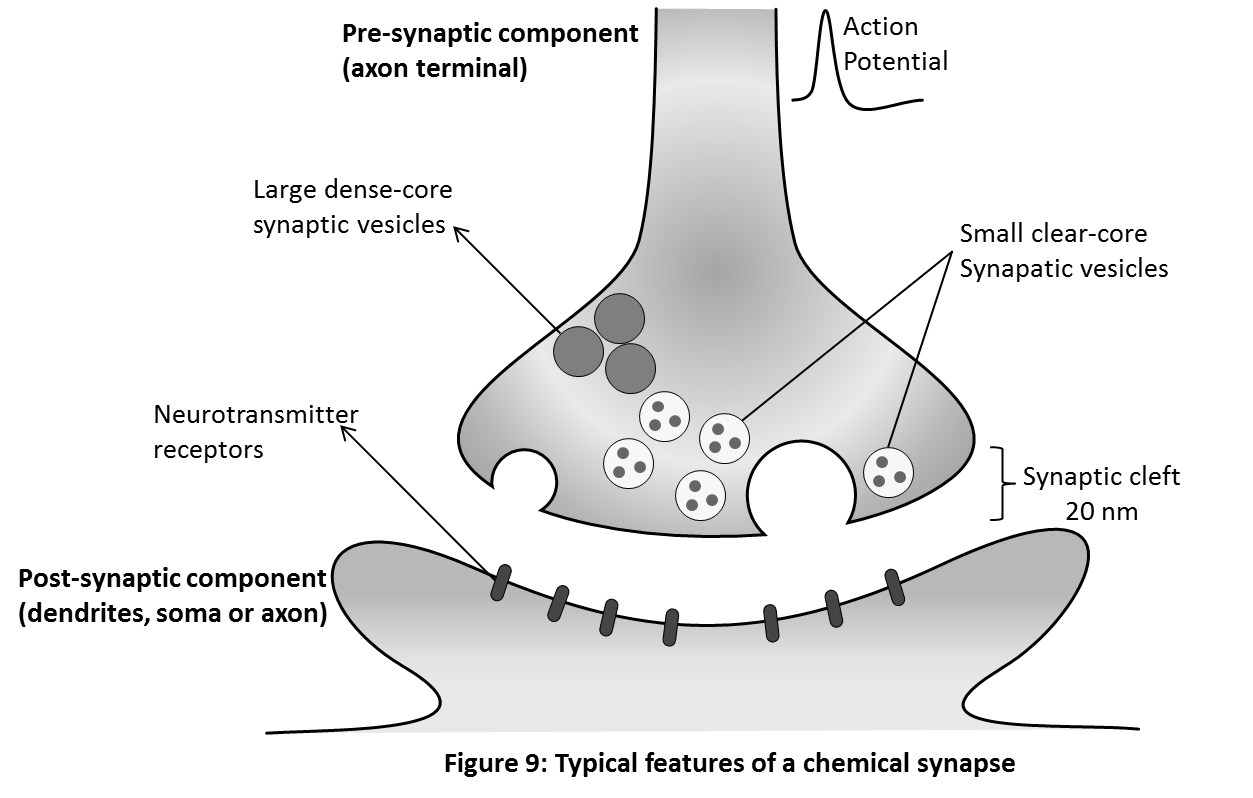


**Figure 12. Typical features of a chemical synapse.**

**Neurotransmission at the chemical synapse:**

The process by which an action potential is converted into a chemical signal and transmitted intercellularly is known as neurotransmission. This process is characterized by a specific sequence of events:

1. An action potential invades the nerve terminal of the pre-synaptic neuron.
2. The change in *Vm* caused by the arrival of the action potential opens voltage-gated Ca^+2^ channels present in the pre-synaptic membrane.
3. Ca^+2^ ions rush into the synaptic terminal.
4. Ca^+2^ ions bind to and mobilize synaptic vesicles to travel to the pre-synaptic terminal membrane.
5. Synaptic vesicles fuse with the terminal membrane and release stored neurotransmitter into the synaptic cleft.
6. Neurotransmitters diffuse across the synaptic cleft and bind to specific receptors on the post-synaptic membrane.
7. Activation of post-synaptic receptors either directly (through ligand-gated ion channel) or indirectly (through G-protein coupled receptor) changes the flow of ions across the post-synaptic membrane increasing (depolarization) or decreasing (hyperpolarization) the probability of an action potential firing.
8. The action of neurotransmitters within the synaptic cleft is terminated by one of three mechanisms:
   - Degradation by enzymes
     1. E.g. Acetylcholine is degraded by the enzyme choline acetyltransferase within the synaptic cleft. Acetylcholinesterase converts acetylcholine into the inactive metabolites choline and acetate, which stop acetylcholine from continuously activating receptors on the post-synaptic membrane.
   - Re-uptake of the neurotransmitter or its precursor by transporters present in the pre-synaptic terminal or glial support cells
     1. Re-uptake is driven by an electrochemical gradient (Na^+^/Cl^-^)
     2. Each neurotransmitter has a specific transporter
     3. Once recaptured, the neurotransmitter can be repackaged for another round of neurotransmission or it can be metabolized by enzymes present in the pre-synaptic terminal
     4. E.g. Dopamine is recaptured by the dopamine transporter, expressed in the pre-synaptic membrane, and then either repackaged into vesicles or metabolized by the enzyme monoamine oxidase.
   - Diffusion away from synapse
9. Retrieval of vesicular membrane from the plasma membrane for re-use.


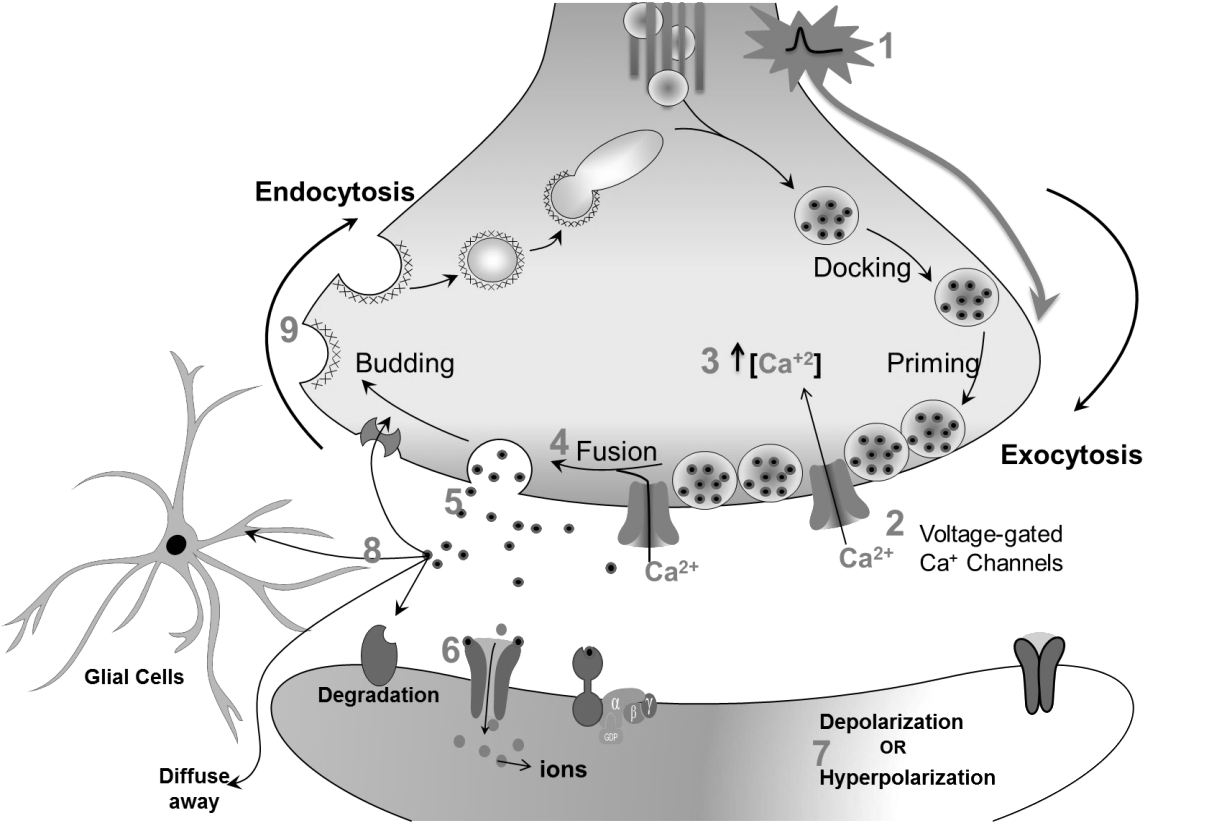
**YOUR NERVOUS SYSTEM AT WORK**

**Figure 13. Summary of the steps involved in synaptic transmission.**

**Sensory Systems**

**EXPERIMENT #1: Jellybean Taste Test**

We use our **sensory system** to navigate and interact with the world around us. Humans have several sensory systems. You are probably familiar with the five basic senses – vision, hearing, touch, taste, and olfaction/smell. Other sensory systems include pain, balance, and temperature. Each sensory system consists of sensory receptors, a neural pathway, and a region of the brain devoted to sensory interpretation and perception. Sensory receptors detect a specific type of sensory stimulus (e.g. light, sound waves, chemical odorants) and convert the stimulus into an action potentials. A neural pathway then relays the electrical signal to the brain. Most sensory information is first filtered through an area of brain called the thalamus. From there the information is further processed in specialized areas in the cortex that are specific for different types of sensory information (Fig 14). However, there are connections between these areas which give a complete sensory perception of the environment.

**Figure 14. Summary of the major sensory systems in the human body.**

In this experiment we will explore how individual sensory systems work together. The perceptions of smell and taste begin when chemical molecules detach from substances and float into the nose or are put into the mouth where they are dissolved and bind to sensory receptors. Separate neural pathways transmit sensory information from the nose or mouth to the brain where we perceive odors or tastes, respectively. Although the neural systems (sensory receptors, neural pathways, and primary brain centers) for taste and smell are distinct from one another, the sensations of flavors and aromas often work together.

**MATERIALS:**

- Jellybeans

**METHODS:**

Work with a partner. Take turns being the experimenter and the subject. The experimenter gives the subject one jellybean according to the three conditions listed below. The subject guesses the jellybean flavor after each condition. The experimenter records two things: (1) what color (flavor) the jellybean is **before** giving it to the subject and (2) what the subject reports as the flavor after each condition. The three conditions are:

(1) Eyes closed and nose plugged

(2) Eyes closed and nose unplugged

(3) Eyes open and nose unplugged

**HYPOTHESIS:** *[Consider the following questions: Under which conditions do you think your partner will be able to correctly determine the jellybean flavor? Why?]*

**OBSERVATIONS:**

| **SUBJECT** | **CONDITION #1 GUESS** | **CONDITION #2 GUESS** | **CONDITION #3 GUESS** |
| --- | --- | --- | --- |
|  |  |  |  |
|  |  |  |  |

**CONCLUSION:**

**EXPERIMENT #2: Two Point Discrimination**

 Sensory receptors in our skin allow us to identify several distinct types of tactile sensations, such as tapping, vibration, pressure, pain, and temperature. What allows us to make these distinctions? First, human skin contains many different kinds of sensory receptors that respond preferentially to various mechanical, thermal, or chemical stimuli, and then convert that specific type of sensory stimuli into action potentials (Fig 15A). Distinct types of tactile information are then transmitted to the brain along specific neural pathways where they converge in a brain region called the **somatosensory cortex**.

**A**


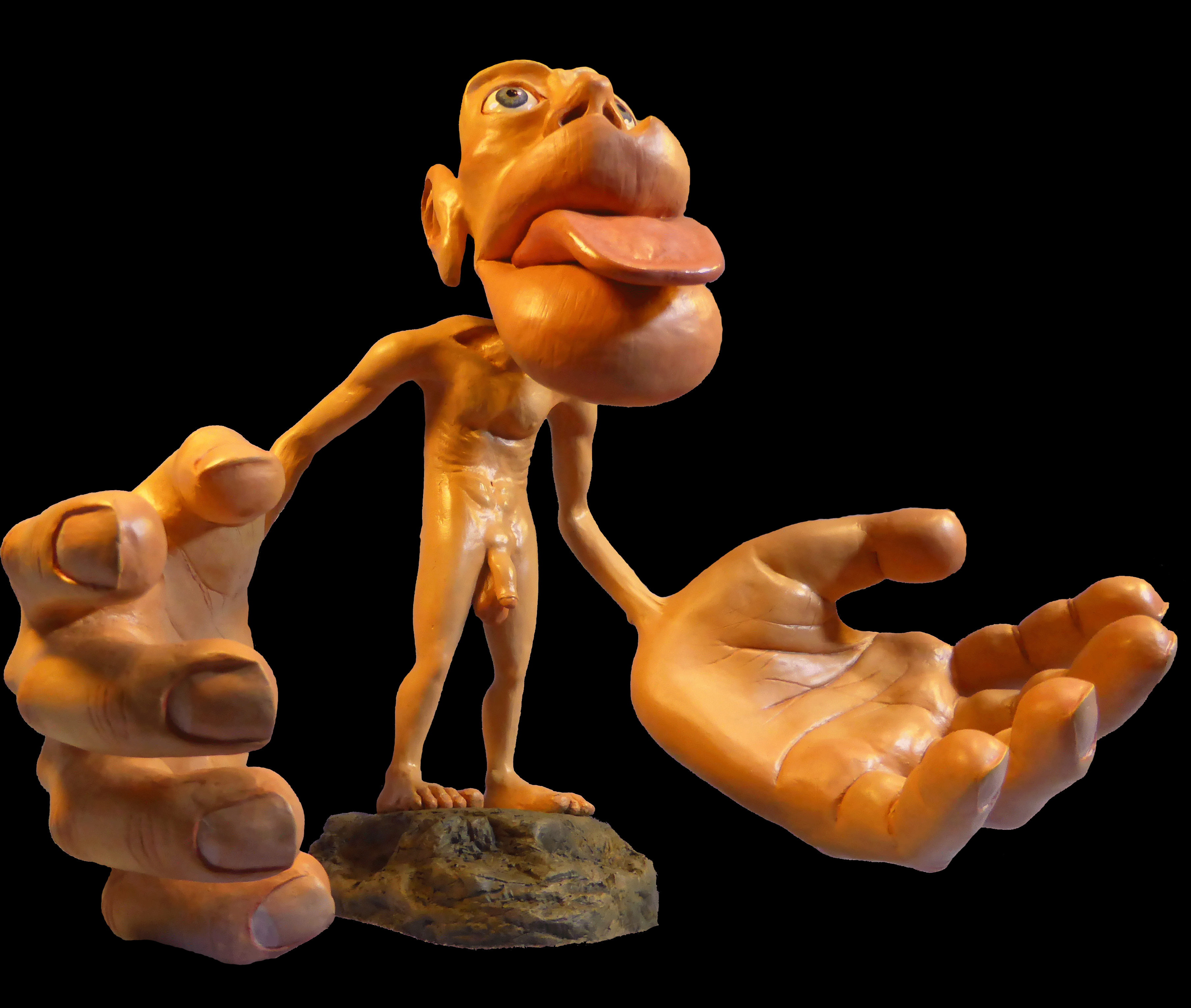
 Sensory input is “mapped” along the somatosensory cortex in a **somatotopic arrangement**. This means that sensory information from each area of the body corresponds to a specific area of the somatosensory cortex. Furthermore, information is organized according to location. For example, neurons in the somatosensory cortex that receive input from the right thumb neighbor neurons that receive input from the right index finger. In this way, a sensory "map" of the body surface is created on a section of the brain surface. *Somatotopy* also means that areas of the body that are finely controlled (i.e. have more sensory receptors within a smaller surface area) have larger portions of the somasensory cortex, whereas regions of the body under coarse control have smaller portions of the somatosensory cortex. For example, the fingertips contain about 100 times more receptors per square centimeter than the skin on the back. Because of this, more neurons must be devoted to receiving fingertip sensations, and consequently the cortical area that receives input from the fingertips is much larger than to the area that receives input from skin on the back. The *sensory homunculus* is a distorted image of the human body that reflects the relative space each body parts occupies on the somatosensory cortex (Fig 15B). In this experiment, we will test the sensation of touch, and evaluate which areas of the body are under fine and coarse control.

**Figure 15. A) Representation of the different types of sensory receptors and the type of sensory information they detect. B) The sensory homunculus** (image by Mpj29 shared under the Creative Commons Attribution-Share Alike 4.0 International license).

**B**

**MATERIALS:**

- Drawing compass

**METHODS:**

Work with a partner. Take turns being the experimenter and the subject. The subject closes his/her eyes and sits still. The experimenter uses the compass to lightly touch the two compass points simultaneously to their partner’s skin. The experimenter then asks the subject if he/she feels one or two points. The experimenter should begin with the compass points at a great distance and continue to adjust the compass points closer and closer together until the subject reports feeling only one point. The experimenter records the smallest distance between the two points of the compass at which the subject can detect both points.

**HYPOTHESIS:**

**OBSERVATIONS:**

| **SKIN AREA TESTED** | **MINIMUM DISTANCE FOR**  **TWO POINT**  **DISCRIMINATION**  in millimeters (mm) |
| --- | --- |
| Tip of index finger |  |
| Thumb |  |
| Palm |  |
| Cheek |  |
| Forehead |  |
| Back of calf/lower leg |  |
| Forearm |  |


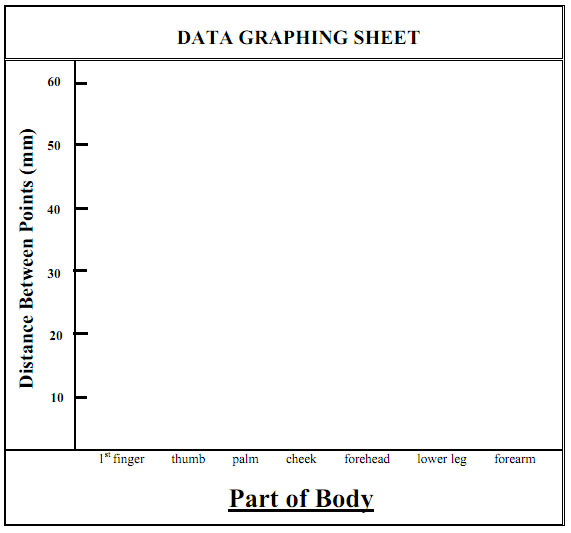


**CONCLUSIONS:**

**QUESTIONS:**

1. What determines our ability to discriminate two points?

2. What is the relationship between the number of sensory receptors in the skin and the size of the somatosensory cortex devoted to interpreting sensation from that body area?

3. How much of the somatosensory cortex is devoted to perceiving sensation in each of the regions you tested (circle one):

Index Finger Large Medium Small

Cheek Large Medium Small

Forehead Large Medium Small

Back of calf Large Medium Small

Forearm Large Medium Small

4. What could you do to change the perception of touch in this experiment? Would this change take place at the level of the skin or the brain?

**Motor System**

**EXPERIMENT #3: Patellar (Knee-Jerk) Reflex**

The **motor system** is the part of the nervous system responsible for the contraction and coordination of muscles. The neuromuscular junction (NMJ) is the area where the axon terminal of a motor neuron makes a connection with a muscle fiber. Upon receiving a signal from the brain, motor neurons release neurotransmitters at an NMJ to cause contraction of the muscle fiber. The more muscle fibers are activated by motor neurons, the stronger the magnitude of the contraction.

**Knee-Jerk (Patellar) Reflex**

There are different types of muscle (skeletal, cardiac, and smooth muscle) that have different functions in the body. Therefore, the motor system is responsible for a variety of tasks, including mechanical movement, heart muscle contraction, and intestinal muscle contraction.

**Figure 16. The knee-jerk patellar reflex test.**

The following exercise will demonstrate how reflex muscle contractions work. Reflexes are involuntary and almost instantaneous muscle movements that have an important role in the protection of the human body. Their fast onset is possible because they do not require integration and instruction from the brain. The knee-jerk or patellar reflex is one type of reflex muscle contraction (Fig 16). It will show you how the sensory and motor systems work together in a matter of milliseconds!

**MATERIALS:**

- Side of your hand
- A partner sitting on a table with legs hanging freely

**METHODS:**

Work with a partner. Take turns being the experimenter and the subject. The subject sits on a table, so that their leg is able to swing freely. The experimenter hits the subject’s leg just below the knee with the side of his/her hand.

**OBSERVATIONS:**

**CONCLUSIONS:**

**QUESTION:**

1. Why do doctors perform this test when you go for an annual check-up?

**EXPERIMENT #4: Reaction Time**

Effective communication between your sensory and motor systems enables you to protect yourself against harmful situations. Whenever someone throws a ball at you, your first instinct is to raise your arms to protect your body. If you touch something hot, you do not have to think twice about removing your hand from the hot object. These and many other reflex movements are dependent on the fast communication between neurons of the sensory system and neurons of the motor system. The speed of this communication process depends in part on the distance the signal has to travel.

In this experiment, we will examine the coordination between the visual sensory system and the motor system. You will be measuring your reaction time to a particular stimulus, which in this case is the drop of a ruler. When performing this exercise, keep in mind how important it is to have quick responses to stimuli every day.

**MATERIALS:**

- Ruler

**METHODS:**

Work with a partner. Take turns being the experimenter and the subject. The experimenter holds the ruler with the 0 inches marker at the bottom. The subject places their hand at the bottom of the ruler with the thumb and fingers around but not touching the 0 inches mark. The experimenter randomly drops the ruler 3 times, and the subject grasps it as quickly as possible (Note: The experimenter should not indicate or gesture when he/she will be dropping the ruler). Record the measurement just above the subject’s thumb where they catch the ruler each time. Convert reaction time from inches to seconds using the table available on the next page.

**HYPOTHESIS:**

**DATA COLLECTION:**

| **REACTION TIME (INCHES)** | | |
| --- | --- | --- |
| **TRIAL #1** | **TRIAL #2** | **TRIAL #3** |

| Distance | Time |
| --- | --- |
| **2 in** | 0.10 sec (100 ms) |
| **4 in** | 0.14 sec (140 ms) |
| **6 in** | 0.17 sec (170 ms) |
| **8 in** | 0.20 sec (200 ms) |
| **10 in** | 0.23 sec (230 ms) |
| **12 in** | 0.25 sec (250 ms) |
| **17 in** | 0.30 sec (300 ms) |
| **24 in** | 0.35 sec (350 ms) |
| **31 in** | 0.40 sec (400 ms) |
| **39 in** | 0.45 sec (450 ms) |
| **48 in** | 0.50 sec (500 ms) |
| **69 in** | 0.60 sec (600 ms) |

| **REACTION TIME (SECONDS)** | | | |
| --- | --- | --- | --- |
| **TRIAL #1** | **TRIAL #2** | **TRIAL #3** | **AVERAGE** |
|  |  |  |  |

**QUESTIONS:**

1. What factors or conditions would affect reaction time?

**CONCLUSION:**

**Nervous System**

**EXPERIMENT #5: External Factors That Modulate Blood Pressure**

The **autonomic division of the peripheral nervous system (ANS)** is responsible for control of internal viscera, cardiac muscle, and glands. There are two major divisions of the ANS: the **sympathetic** and **parasympathetic divisions of the ANS** (Fig 17). For most body functions, these two divisions work in opposite directions to accurately control organ function. For example, the sympathetic division increases heart rate, whereas the parasympathetic division decreases heart rate.

In this exercise, we’re going to explore how external factors, such as drugs or exercise, affect the two divisions of the ANS using blood pressure as an endpoint. You have been learning how to develop and test hypotheses, so here is your chance to formulate and test your own! Do you think caffeine activates the sympathetic or parasympathetic nervous system? How would you test this? What about exercise? Stress? Anything else?

**Figure 17. Peripheral Nervous System.**

**MATERIALS:**

- Blood pressure cuffs
- Caffeine drinks
- Stop watches
- Stairs
- Be creative: consider what items you have in your backpack, could those alter blood pressure somehow?

**METHODS:**

Work with a partner. Using the materials available (or anything else you have available) formulate a testable research question and hypothesize how some external factor (i.e. drug (caffeine), exercise, stress) affects the balance between the sympathetic and parasympathetic divisions of the ANS. Then test your hypothesis.

**HYPOTHESIS:**

**OBSERVATIONS:**

**CONCLUSIONS:**

**NEUROLOGICAL DISEASES**

**Pathophysiology of Parkinson disease (PD):**

PD results from the loss of pigmented cells in the substantia nigra (SN), an area of the brain known to contain dopaminergic neurons. These neurons project to the basal ganglia, where they coordinate and modulate voluntary movement. Additionally, protein aggregations within neuronal cell bodies, called Lewy Bodies, are observed throughout the brain.

**Pathophysiology of Alzheimer’s disease (AD):**

The causes and mechanisms of the brain abnormalities underlying AD are not yet fully understood. There are two characteristic pathophysiological features: 1) the loss of neurons and synapses in the cerebral cortex and hippocampus, which results in extreme shrinkage, or *atrophy*, of the brain; and 2) the abnormal accumulation of beta-amyloid plaques and neurofibrillary tangles. The small fibrillary peptide, *beta amyloid*, aggregates in the spaces around synapses (*neuritic plaques*) and a modified form of the protein tau accumulates in the cell bodies of neurons (*neurofibrillary tangles*). In all forms of AD plaques and tangles mostly develop in brain regions important for memory and intellectual functions, including the hippocampus, basal forebrain, and cerebral cortex.

**Pathophysiology of Amyotrophic Lateral Sclerosis (ALS):**

ALS is a type of motor neuron disease characterized by the gradual degeneration and death of motor neurons. Upper motor neurons in the brain transmit signals to lower motor neurons in the brainstem and spinal cord, which in turn send the message on to particular muscles. In ALS, both upper and lower motor neurons degenerate.

ALS is present in two forms: familial ALS (FALS) and sporadic ALS (SALS). However, whether a patient obtained the disease sporadically or by familial origin is indistinguishable. Familial ALS forms 10% of ALS cases, and 20% of these patients have a mutation in the Cu^2+^/ Zn^2+^ superoxide dismutase 1 (SOD1). Sporadic ALS forms 90% of the ALS cases and 12% of these patients also show the SOD1 mutation. Studies have shown that FALS and SALS have the same pathology, and it is hoped that therapies effective in SOD1 models can translate and be useful to treat to both familial and sporadic ALS.

**Pathophysiology of Cerebrovascular disease (stroke):**

A stroke occurs when either a blood vessel bringing oxygen and nutrients to the brain bursts or is clogged by a blood clot or some other particle. This deprives the brain of blood, causing the death of neurons within minutes. The initial insult triggers a cascade of cell death events, including oxidative stress and inflammation, which can increase the infarct size (area of tissue death) and lead to permanent brain damage. For these reasons, time is of the essence! The faster a patient received medical treatment, the less damage is likely to occur.

**Figure 4**. Pathophysiology of stroke.

**CASE STUDIES**

http://memphisvascular.com/patient-education/stroke/

**Case Study #1:**

A 65 year-old man presented with a resting tremor in both hands. The tremor began approximately 10 years earlier in his right hand and had become increasingly more pronounced. He also described a “stiff, slow” feeling throughout his body, which prevented him from performing rapid coordinated movements. His posture was stooped and there was tendency to shuffle when he walked. Examination revealed that movements were difficult to initiate and performed slowly (bradykinesia).

Over the course of five years, these motor symptoms became progressively worse, affecting the patient’s ability to perform everyday tasks, such as dialing a telephone number or walking from the kitchen to the living room unaided. Treatment was able to relieve symptoms and slow the progression of the disease; however the dose of the medication had to be increased gradually. Ultimately the treatment itself began to impair quality of life. In the final stages of the disease, the patient suffered not only from severe motor symptoms, but also cognitive and behavior symptoms, including dementia and depression. The man survived into his 79^th^ year, passing away from an infection of pneumonia.

Can you identify this disorder?

**Case Study #2:**

The patient was a 72-year-old woman who was moderately overweight (BMI of 28) and had been smoking one pack of cigarettes a day for the past 40 years. She awoke one morning with weakness on the right side of her body. She was moderately confused, seeing double, and slurring her speech. When she attempted to walk to the bathroom, she stumbled and fell twice. Her daughter immediately called 911. The patient was unresponsive when emergency personnel arrived 20 minutes later, but slowly regained consciousness over the following two days. Once fully alert, she was experiencing paralysis and loss of sensation on the right side of her face and arm and an inability to speak but was responsive to verbal commands (e.g. blink once for yes, twice for no).

The patient was monitored over the following five years. Physical therapy helped to improve the symptoms of right hemi paralysis dramatically; the patient was able to use her arm and hand for tasks such as buttoning a shirt, drinking water from a cup, and writing. Additionally, the patient regained partial sensation in the affected areas. Her ability to speak was also recovered; however her speech was slightly slurred and slowed.

Can you identify this disorder?

**Case Study #3:**

The patient was a 53-year-old male who presented with the complaint of a left foot drop that was affecting his ability to walk normally. Additionally, he reported muscle twitching and cramping in both lower limbs. Examination revealed mild weakness in all extremities, and mild difficult with speech (dysarthria) and swallowing (dysphagia).

The patient was monitored over the course of the next three years. During this time, motor symptoms became more pronounced and severe weight loss was noted due to muscle atrophy. Eventually the patient was unable to stand or walk, he had complete loss of the ability to use his hands and arms, and chewing and swallowing was increasingly difficult. Cognitive function remained intact, however because of the progressive motor loss, the patient became anxious and depressed. Respiration became increasingly labored, as the diaphragm and intercostal muscles (rib cage) weakened. These symptoms were treated for a short period of time, however the disease progression soon overcome all respiratory treatment options. The patient elected to enter hospice care and died in his 55^th^ year of respiratory failure.

Can you identify this disorder?

**Case Study #4:**

The patient was a 72 year old woman with a one year history of confusion, apathy, and insomnia. Six months prior to the initial visit she had moved from California to Michigan and had great difficulty learning to navigate in her new surroundings. She had a history of depression, but no other serious neurological diseases. Examination noted intact attention, intact reading and writing, and intact long-term memory. Mood stabilizers were prescribed to treat her depressive mood.

She was seen occasionally over the next few years to follow-up on her depression. Notes from a visit in her 75^th^ year said that her daughter was having trouble getting her dressed and out of the house. She had decreased energy and outbursts of aggression. The neurological examination noted her thoughts wandered easily from topic to topic and her reading and writing skills were diminished. She was hospitalized at the age of 78 for dementia and paranoia. She moved to a care facility at 79 and died within a year due to an infection arising from a pressure ulcer.

Can you identify this disorder?

**References**

1. Bear MF, Connors BW, Paradiso MA. (2006). Neuroscience Exploring the Brain. Third edition, Lippincott Williams and Wilkins.

2. Kandel ER, Schwartz JH, and Jessell TM. (2000). Principles of Neural Science. Fourth edition, New York: McGraw Hill Health Professions Division.

3. Dana Foundation. www.dana.org

4. Brain Facts. www.brainfacts.org

5. Neuroscience for Kids. www.faculty.washington.edu/chudler/neurok.html
