# Supplementary material for: Bridge to neuroscience workshop: An effective educational tool to introduce principles of neuroscience to Hispanics students
Source: PLoS One. 2019 Dec 12;14(12):e0225116. doi: 10.1371/journal.pone.0225116 (PMC6907774; doi:10.1371/journal.pone.0225116)
Supplement: S5 File — (DOCX) [file pone.0225116.s006.docx]

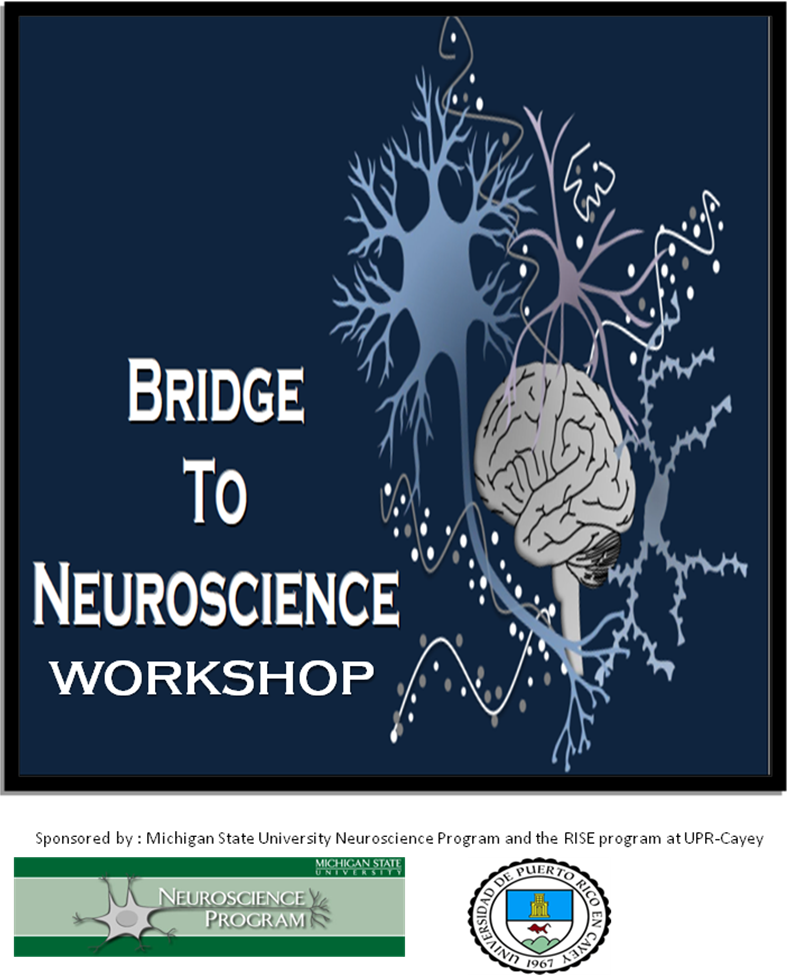


Sponsored by: Michigan State University Neuroscience Program.


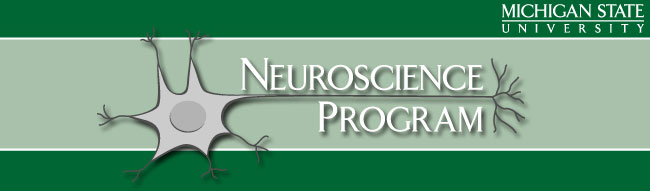


To find pictures of today’s workshop and for more information go to:

**https://www.msubpnp.com/workshops**

**Bridge to Neuroscience Workshop-Book**

**Contributors**

Alexandra Colón-Rodríguez, PhD

Eileen S. Rodriguez Tapia, PhD

Chelsea Tiernan, PhD

Brenda Marrero-Rosado, PhD

Chelsea Hutch, PhD

Carla Dams, MS

**Editors**

Alexandra Colón-Rodríguez, PhD

Chelsea Tiernan, PhD

**BPNP Director**

William D. Atchison, PhD

Michigan State University

1355 Bogue Street

B307 Life Science Bldg.

East Lansing, MI 48824

atchiso1@msu.edu

**EL SISTEMA NERVIOSO**

Piensa en tu equipo deportivo favorito o en algún coro que hayas escuchado recientemente. Puedes pensar en algo que estos tengan en común? Ambos están compuestos por un por un grupo de personas y por un director principal: un entrenador en el caso del equipo deportivo y un director en el caso del coro. Esta persona líder tiene como objetivo influir y coordinar el comportamiento del grupo. En adición, esta persona recibe e integra información de cada miembro del grupo para poder llegar a la meta deseada: ganar un campeonato o llevar a cabo un concierto musical encantador.

El sistema nervioso (SN) puede ser visto como un entrenador o director de nuestro cuerpo porque este trabaja de una manera muy similar. Por ejemplo, el SN recibe información de todas las partes del cuerpo para mantener las funciones de nuestro cuerpo trabajando en óptimas condiciones todos los días.

**Divisiones del sistema nervioso**


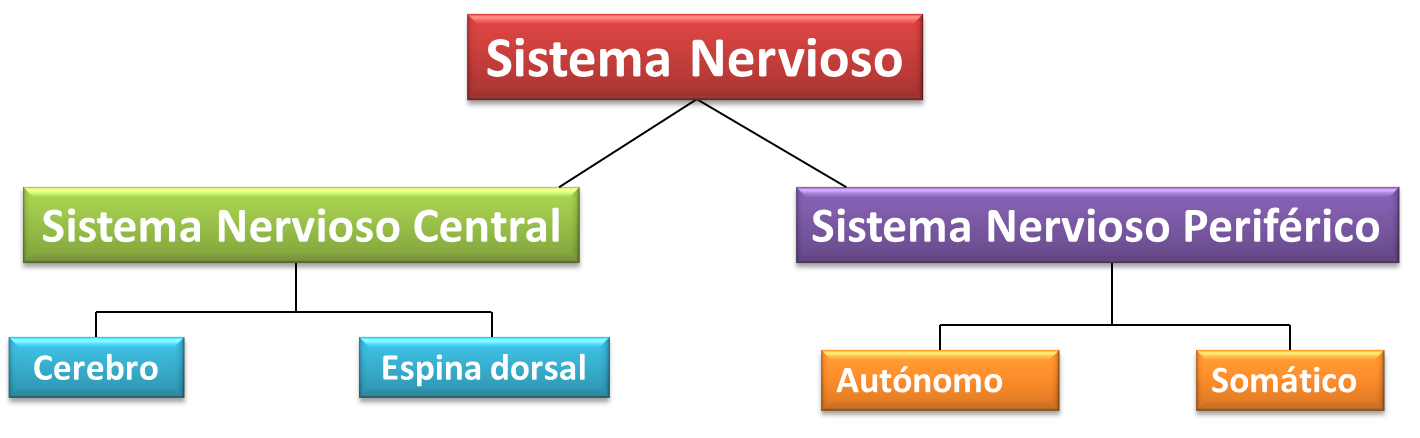
 El SN está dividido principalmente en el sistema nervioso central y el sistema nervioso periférico. El sistema nervioso central está compuesto por el cerebro y la espina dorsal. El sistema nervioso periférico está compuesto por todos los nervios y ganglios que se comunican con los órganos. El sistema nervioso periférico esta subdividido en el sistema nervioso autónomo y el sistema nervioso somático. La fig 1 hace referencia a la organización del SN.

**Figura 1. Divisiones del sistema nervioso.**

*Sistema nervioso central*

El sistema nervioso central (SNC) se conoce como central porque es responsable de recibir información de todas las partes de nuestro cuerpo la cual utiliza para regular el comportamiento y la función de todas las partes del cuerpo. A continuación encontraras una descripción de los dos componentes del SNC.

*Sistema nervioso central: Cerebro*

El cerebro es el centro de control del cuerpo. El cerebro humano pesa un promedio de 3 libras y este está compuesto de dos hemisferios cerebrales: derecho e izquierdo. La superficie de cada hemisferio cerebral contiene unos espacios llamados surcos y unas estructuras parecidas a “colinas” llamadas giros. Estas estructuras permiten que una mayor área de corteza cerebral pueda mantenerse en el interior del cráneo. Esto es muy importante porque a medida que el área de la corteza cerebral aumenta también aumenta la capacidad del cerebro para recibir y procesar información.


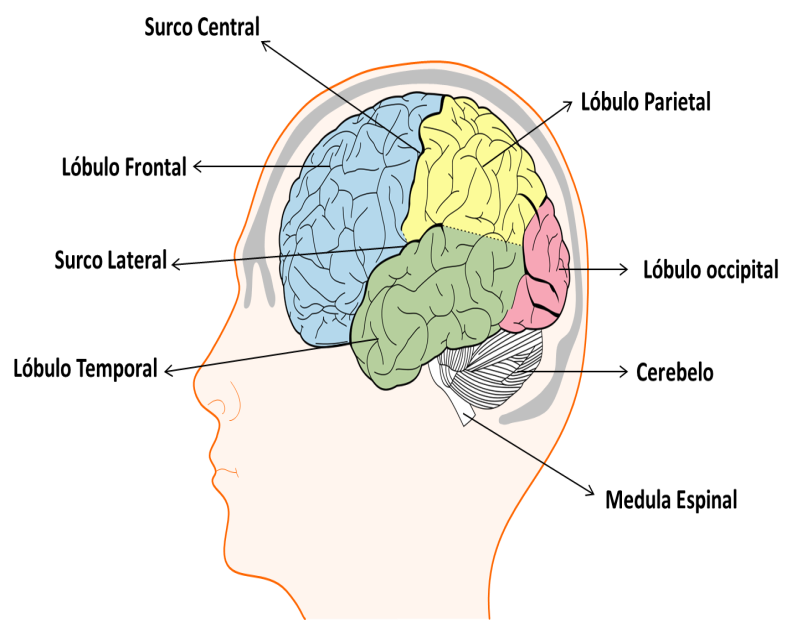


**Figura 2. Estructuras del cerebro.**

El patrón de los surcos y giros dividen cada hemisferio cerebral en cuatro lóbulos cerebrales: frontal, parietal, temporal y occipital (Fig 2). Cada lóbulo está asociado con una serie de funciones específicas que nos permiten percibir e interactuar con lo que nos rodea. El lóbulo frontal está localizado en la parte delantera del cerebro y procesa información relacionada a la planificación, el razonamiento, las expresiones emocionales, el control del comportamiento, información que nos ayuda a juzgar y información relacionada al movimiento. El lóbulo parietal se encuentra en la parte posterior del lóbulo frontal y esta parte es responsable por la percepción de estímulos en la piel incluyendo el tacto, la temperatura, el dolor y la presión. El surco central es una marca guía en el cerebro y separa el lóbulo frontal del parietal. El lóbulo temporal está localizado inferior al lóbulo frontal y parietal y este es responsable de procesar información del sistema auditivo y la memoria. Otra marca guía en el cerebro es el surco lateral y este separa el lóbulo temporal del lóbulo frontal y el parietal. En la parte posterior del cerebro se encuentra el lóbulo occipital y este procesa información asociada con la visión.

**B**

**A**


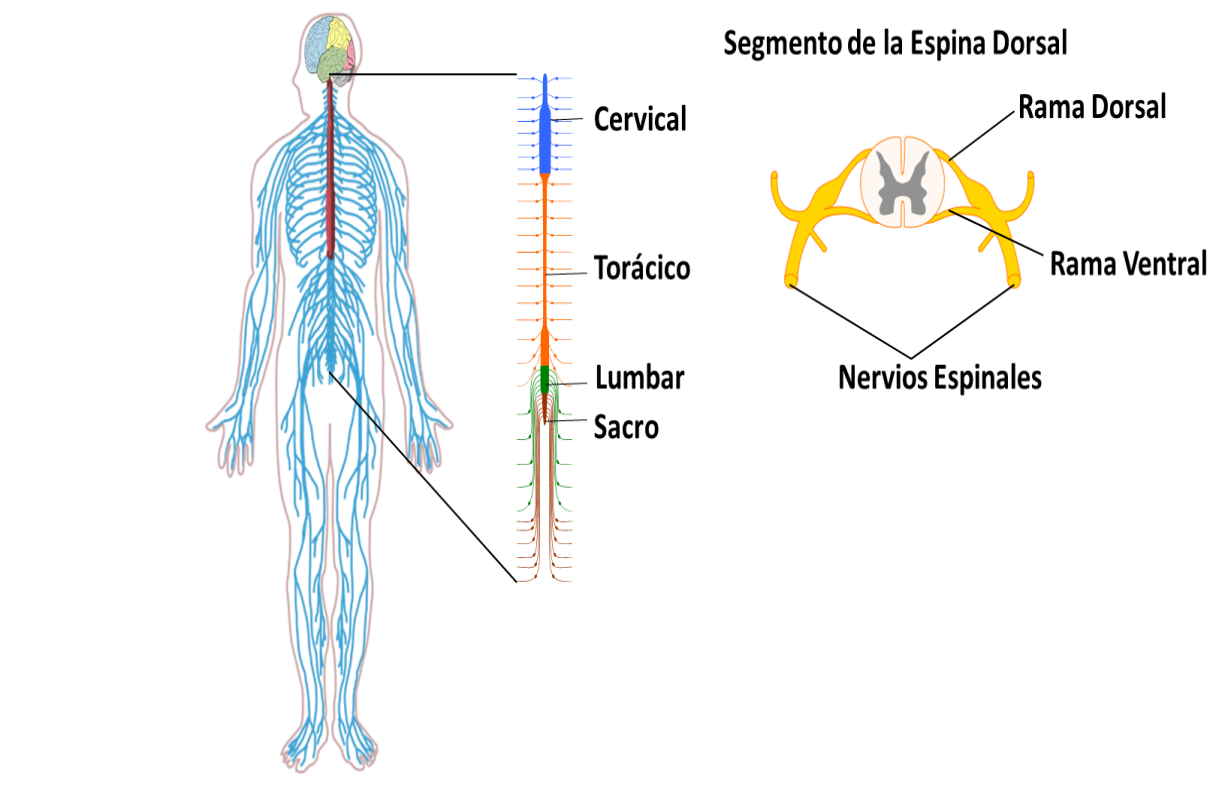
 El cerebelo (palabra del latín que significa “cerebro pequeño”) está localizado en la parte inferior de los hemisferios cerebrales. Esta parte del cerebro no inicia movimiento pero regula la coordinación y precisión en tiempo y espacio de las actividades motoras. El tallo cerebral (medula espinal) se encuentra en la parte posterior del cerebro y se extiende formando la espina dorsal. En humanos el tallo cerebral incluye el mesencéfalo, el puente troncoencefálico y la médula oblonga. El tallo cerebral es responsable de controlar la respiración, los latidos del corazón, el estado de conciencia y los ciclos del sueño.

**Figura 3. Estructura de la espina dorsal. A) Divisiones de la columna vertebral. B) Segmento de la espina dorsal.**

Sistema nervioso central: Espina dorsal

La espina dorsal representa la conexión principal entre el cerebro y el sistema nervioso periférico y por lo tanto es la ruta principal para el intercambio de información entre estas dos partes del SN. La espina dorsal mide de 40 a 50 cm de largo y 1 a 1.5 cm de diámetro. En humanos la espina dorsal está protegida por la columna vertebral. Los huesos que construyen la columna vertebral son conocidos como vertebras. Existen 31 segmentos en la columna vertebral: 8 cervicales, 12 torácicos, 5 lumbares, 5 sacros y 1 coxígeo (Fig 3A). Un par de nervios espinales se originan en cada segmento de la columna vertebral llegando a un total de 31 nervios espinales. Cada nervio espinal está compuesto por dos raíces: la raíz ventral y la raíz dorsal (Fig 3B). Los nervios en la raíz ventral llevan información motora hacia los músculos esqueletales. Los nervios en la raíz ventral traen información sensorial a diferentes partes del cerebro.

El sistema nervioso periférico

El sistema nervioso periférico (SNP) es la división del SN que contiene los nervios que residen fuera del cerebro y la espina dorsal. El SNP no está protegido por una columna de hueso, lo cual lo hace más vulnerable a toxinas y a daños físicos.

*El sistema nervioso periférico: el sistema nervioso* autónomo

La división autónoma del SNP regula las funciones de los órganos internos como el corazón, estomago, intestino y glándulas. Dos divisiones del sistema autónomo controlan nuestras respuestas en dos tipos de situaciones diferentes. El sistema nervioso simpático (SNS) prepara el cuerpo para responder apropiadamente a situaciones de emergencia que causan estrés. Esto se conoce como la respuesta: “fight or flight”. El sistema nervioso parasimpático (SNP) prepara al cuerpo para responder a situaciones que no son de emergencia. Esta respuesta es llamada: “rest and digest”. Las neuronas en cada sistema se comunican con los mismos músculos y glándulas, sin embargo, sus acciones son opuestas. Por ejemplo, durante una situación de emergencia el SNS aumenta la presión sanguínea, los latidos del corazón y disminuye la digestión para ayudarte a manejar la situación. Durante una situación no estresante el SNP trabaja para ahorrar energía; bajando la presión sanguínea, modulando los latidos del corazón y aumenta el proceso de digestión.

La última división del sistema autónomo nervioso es el sistema nervioso entérico (SNE). El SNE es también conocido como el “pequeño cerebro del intestino” porque las neuronas que controlan las funciones principales del sistema digestivo residen dentro en la pared del tracto gastrointestinal desde el esófago hasta el ano. El SNE contiene 100 millones de neuronas, cantidad similar a la espina dorsal. Estas neuronas trabajan juntas para controlar las funciones principales del sistema digestivo: contracciones y relajaciones para mover el contenido intestinal, absorción de nutrientes y secreción de hormonas.

*El sistema nervioso periférico: el sistema nervioso* somático

La segunda división del SNP es el sistema nervioso somático. Contrario al sistema nervioso autónomo, el sistema somático regula el control del movimiento voluntario del cuerpo. Este sistema incluye nervios que llevan información sensorial al SNC: espina dorsal y cerebro. Estos nervios que comunican información hacia el SNC se les conoce como nervios aferentes. En adición, el sistema somático incluye nervios que “hablan” con el musculo esqueletal para producir la contracción del mismo. Estos nervios que se comunican con el músculo y que lleva información hacia fuera del SNC se llaman nervios eferentes.

Para obtener un conocimiento más completo sobre como el sistema nervioso trabaja se necesita conocer la unidad básica de éste sistema: **la neurona**. A continuación encontraras información sobre la función y la estructura única de las neuronas. ¡Todas estas características le permiten a las neuronas trabajar juntas para hacer que el sistema nervioso funcione!


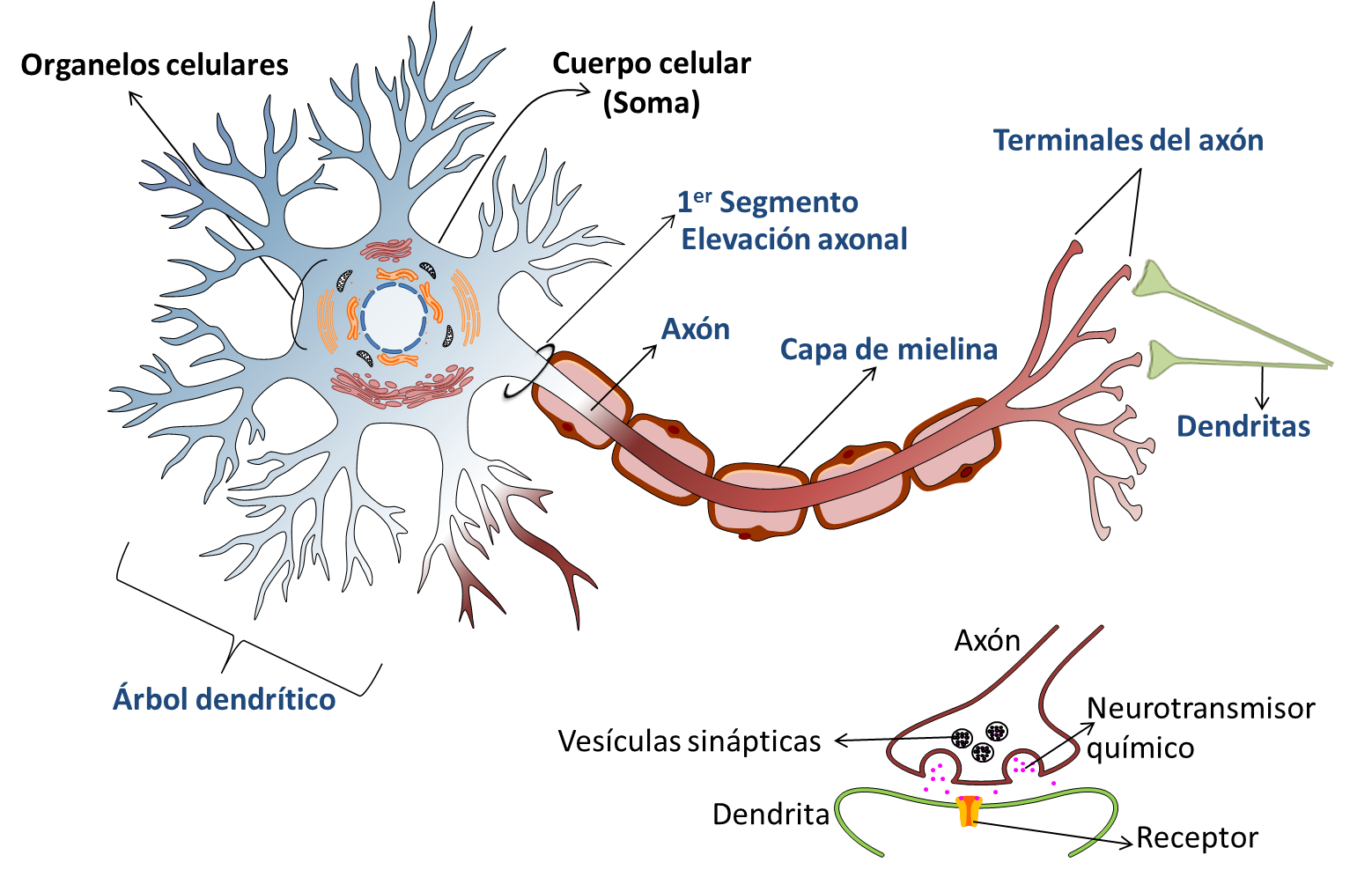
**LA NEURONA**

**Figura 4. Estructura de las neuronas.**

Las neuronas son responsables de llevar a cabo la función más importante del sistema nervioso, la cual es: transferencia de información ya sea intercelular o intracelular. Estas células cuentan con estructuras que las especializan para la transferencia de información (Fig 4). Estas estructuras son:

1. El **soma**, también llamado cuerpo celular, es el centro de control de la neurona. Recibe señales eléctricas de las dendritas y conduce señales eléctricas al axón. El soma también contiene muchos orgánulos celulares que se encuentran en las células no neuronales, incluidos el núcleo, las mitocondrias, el retículo endoplásmico rugoso y liso, el aparato de Golgi, el ribosoma y otros organelos subcelulares necesarios para la síntesis de proteínas y otros procesos metabólicos.
2. **Dendritas:** se originan en el cuerpo celular o soma y se ramifican varias veces formando un árbol dendrítico. Estas estructuras expresan receptores para neurotransmisores y esta característica hace que las dendritas se especialicen en recibir información de otras neuronas.
3. **Axón:** se origina en el cuerpo celular o soma y es capaz de generar y propagar señales eléctricas, las cuales se le conoce como: Potenciales de acción. Este potencial de acción se origina en el primer segmento del axón o el “axón hillock” y se propaga hasta llegar al terminal del axón o terminal nervioso. El axón contiene proteínas que atraviesan la membrana, (proteínas transmembranales), que capacitan a este a generar el potencial de acción. El largo del axón puede ser unos cuantos micrómetros o puede llegar a medir varios metros dependiendo del tipo de neurona y el organismo.
4. **Terminal del axón:** también llamado terminal nervioso o terminal sináptico. Contiene las vesículas sinápticas las cuales a su vez contienen los neurotransmisores (molécula que transmite el mensaje de neurona a neurona). También contiene la maquinaria necesaria para producir, almacenar y liberar los neurotransmisores.

**Transporte Axonal**

En las neuronas, cada compartimiento posee una función importante durante la transmisión sináptica. Por ejemplo, las dendritas reciben información, el axón genera y propaga señales eléctricas llamadas potencial de acción y el terminal sináptico posee los neurotransmisores y los libera. Para que cada compartimiento neuronal ejerza su función efectivamente tiene que tener las proteínas necesarias, unos ejemplos serian: los receptores en dendritas o los canales iónicos en el axón.

La mayor parte de las proteínas son producidas en el cuerpo celular de las neuronas, por lo tanto, esta tiene que desarrollar una estrategia para que cada compartimiento pueda tener el contenido proteico que se requiera para poder funcionar normalmente. El ***transporte axonal*** es un proceso que ayuda a mantener comunicación entre el cuerpo celular de una neurona y el terminal nervioso de la misma manera que ayuda a transportar material desde el cuerpo celular o soma al terminal o viceversa.

*¿Cómo funciona?*

El axón contiene microtúbulos, el cual forma parte de su citoesqueleto, y este forma el “camino” para el transporte. Proteínas motoras también participan en el transporte cargando el material (o cargo) a transportarse. Estas proteínas motoras serian el “auto” que carga a los pasajeros los cuales serían el cargo (Fig 5). Dos ejemplos de proteínas motoras serian: kinesina (transporta cargo desde el cuerpo celular hasta el terminal) y dineina (transporta cargo desde el terminal hasta el cuerpo celular).


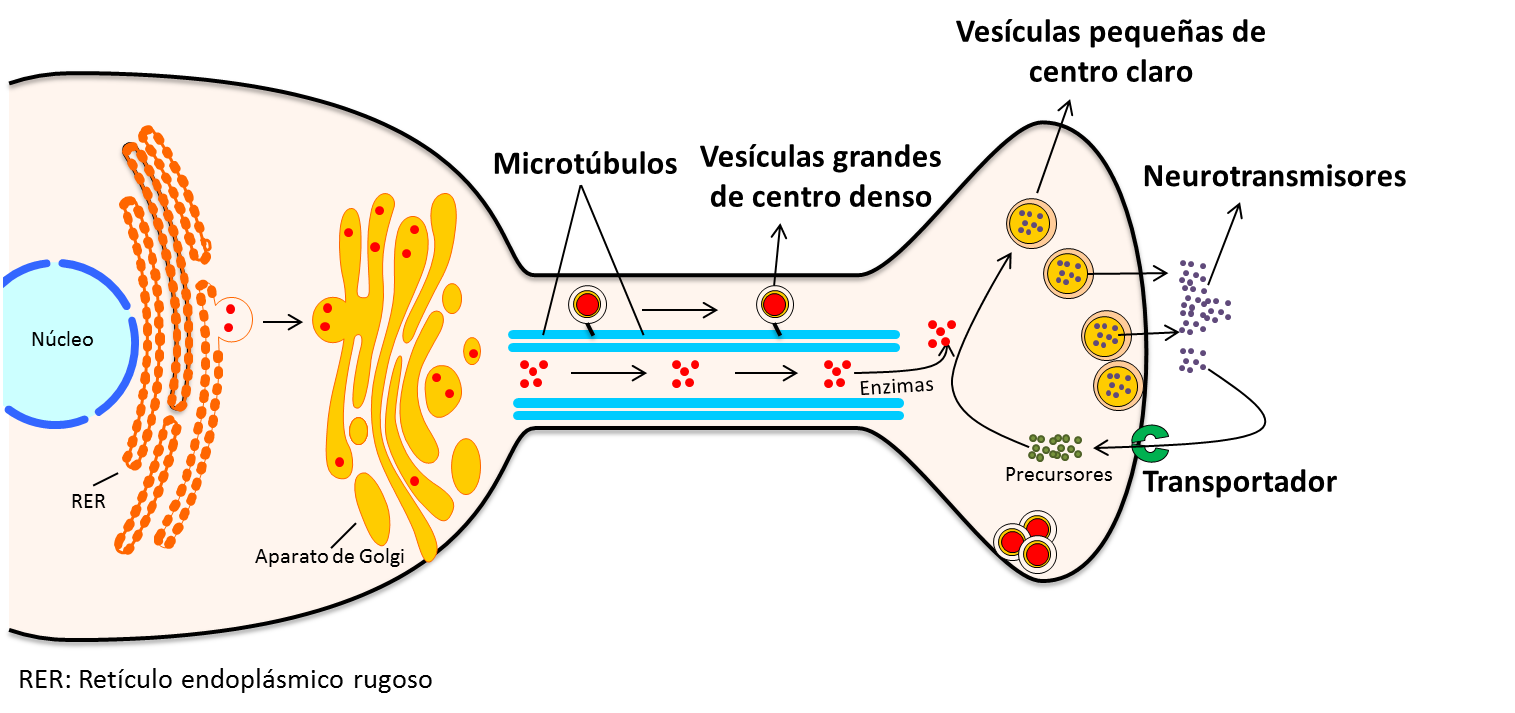
 Existen dos tipos de transporte axonal: rápido y lento. Transporte axonal rápido transporta organelos membranales grandes como por ejemplo, vesículas secretoras, precursores de vesículas sinápticas, mitocondrias y neuropéptidos almacenados en vesículas secretoras. Transporte axonal lento transporta proteínas citosólicas (enzimas) como las que se encargan de producir algunos neurotransmisores y elementos citoesquelatales como por ejemplo neurofilamentos, micro túbulos, etc.

**Figura 5. Transporte axonal**

**Señales eléctricas en neuronas**

**Figura 5. Transporte axonal.**

Las células nerviosas pueden generar y producir señales eléctricas: ***“todo se basa en el movimiento de iones a través de la membrana celular”***. Todas las células, nerviosas y no nerviosas, tienen un potencial membranal (Vm) negativo a causa de la distribución desigual de iones a través de la membrana.

Las células nerviosas en reposo poseen un Vm que puede estar en el rango desde -40 mV hasta -90 mV y a este se le conoce como potencial membranal de reposo (Vm de reposo).

*¿Cómo se genera el potencial membranal de reposo?*

Dos factores son sumamente importantes en la generación del Vm de reposo. Al primero se le conoce como la *distribución desigual de iones* a través de la membrana. Por ejemplo, potasio (K^+^) es el ion predominante en el interior de la célula. Sodio (Na^+^) es el ion predominante en el exterior de la célula. Cloro (Cl^-^) es el anión predominante el exterior de la célula. Aniones impermeables como proteínas, ácidos nucleicos y lípidos están secuestrados en el interior de la célula. Esto crea un ***gradiente de concentración*** y también genera un gradiente eléctrico (Fig 6).

Ahora bien, ¿que determina y ayuda a mantener este gradiente de concentración? Lo que ayuda a crear y a mantener este gradiente de concentración son los transportes activos: la bomba de Na^+^/K^+^ y la bomba de Cl^-^. La bomba de Na^+^/K^+^ constantemente transporta iones de Na^+^ hacia el exterior de la célula para mantener la concentración extracelular de Na^+^ mucho más alta que la concentración intracelular. También transporta iones de K^+^ hacia dentro de la célula para mantener la concentración de K^+^ mucho más alta en el interior que en el exterior de la célula. Esta bomba transporta tres iones de Na^+^ hacia fuera por cada dos iones de K^+^ que transporta hacia dentro de la neurona.


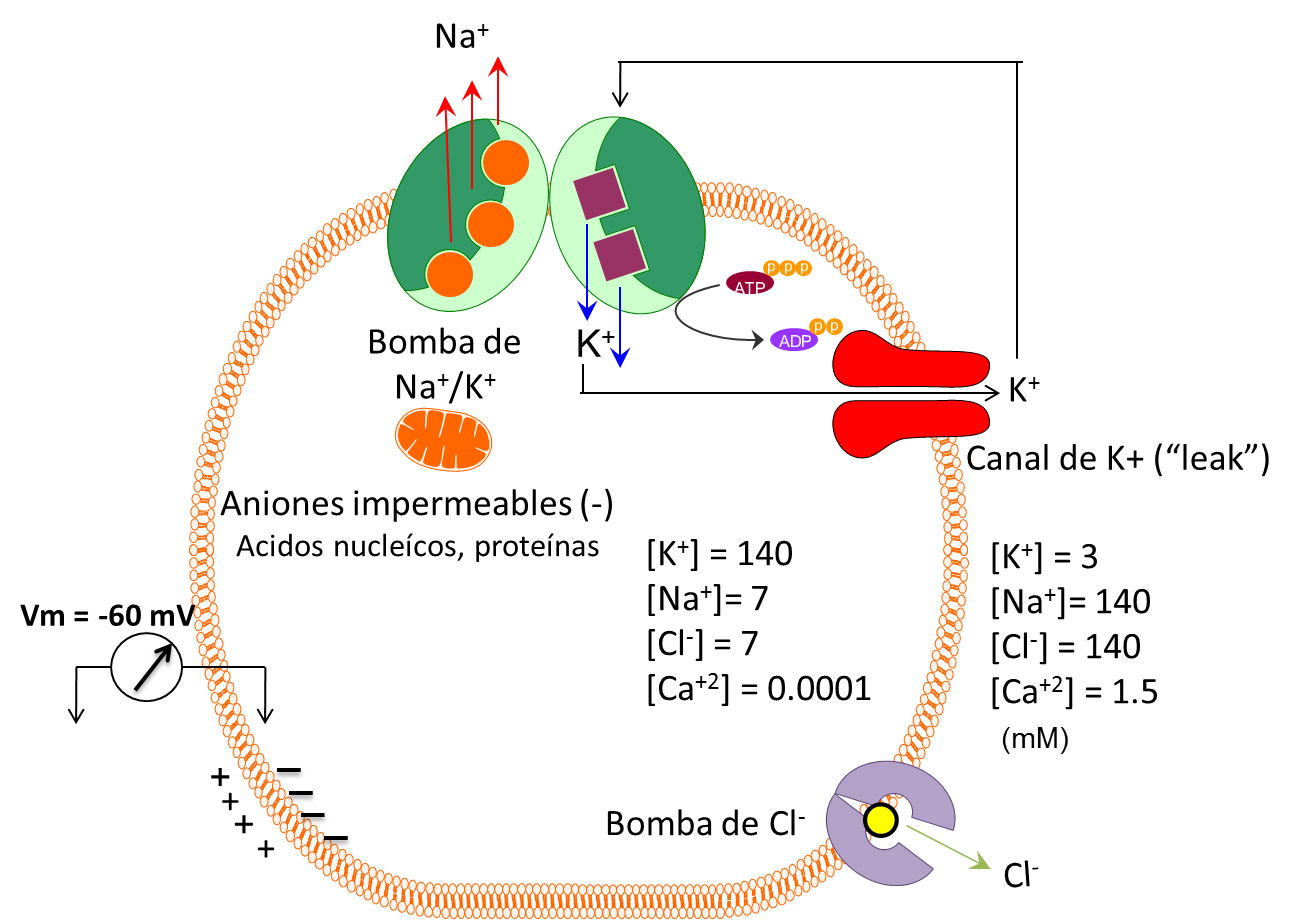


**Figura 6. Distribución de iones en la neurona.**

**Figura 6. Distribución de iones en neuronas**

El segundo factor importante en la generación del Vm de reposo es la permeabilidad de la membrana celular. Esta última es semi-permeable, es decir, posee poros en la membrana formados por proteínas transmembranales produciendo canales iónicos. El canal de K^+^ llamado “Leak K^+^ channel” siempre está abierto y cuando la neurona está en reposo K^+^ fluye hacia el exterior de la neurona debido a su gradiente de concentración. Pérdida completa de K^+^ en el interior de la neurona se previene gracias a interacciones electromagnéticas entre K^+^ y aniones impermeables. Por esta razón siempre hay un exceso de cargas negativas en el interior de la neurona en reposo y al medir el potencial membranal en el interior de la neurona este es negativo cuando lo comparas con el exterior.

El potencial membranal de reposo puede variar dependiendo del estado de la neurona. Si este se despolariza quiere decir que esta menos negativo; si este se hiperpolariza quiere decir que está más negativo.

**Potencial de acción**

Para que una neurona genere un potencial de acción, esta requiere una despolarización inicial.cEsta despolarización puede ser producida luego de la acción de algún neurotransmisor, por la inyección artificial de corriente positiva o simplemente por la entrada de iones de Na^+^ a través de canales de Na^+^. En la generación de esta señal eléctrica se encuentran dos canales iónicos envueltos: Canales de Na^+^ sensitivos a voltaje y canales de K^+^ sensitivos a voltaje. Las proteínas que forman cada uno de estos canales contienen secuencias de amino ácidos que son sensitivas al voltaje a través de la membrana. Cuando este se despolariza esta secuencia de amino ácidos sufre un cambio de conformación que causa la apertura del canal y el flujo de iones ya sea Na^+^ o K^+^, dependiendo del tipo de canal. El canal de Na^+^ sensitivo a voltaje abre cuando el potencial membranal alcanza un valor de -50 mV. El canal de K^+^ sensitivo a voltaje abre cuando el potencial membranal alcanza un valor de 0 mV.

Cuando un electrodo de vidrio es insertado en el citosol de una neurona cerca del “axon hillock” y este se conecta a un amplificador el potencial membranal de reposo se puede observar y este puede ser un valor cerca de -65 mV.


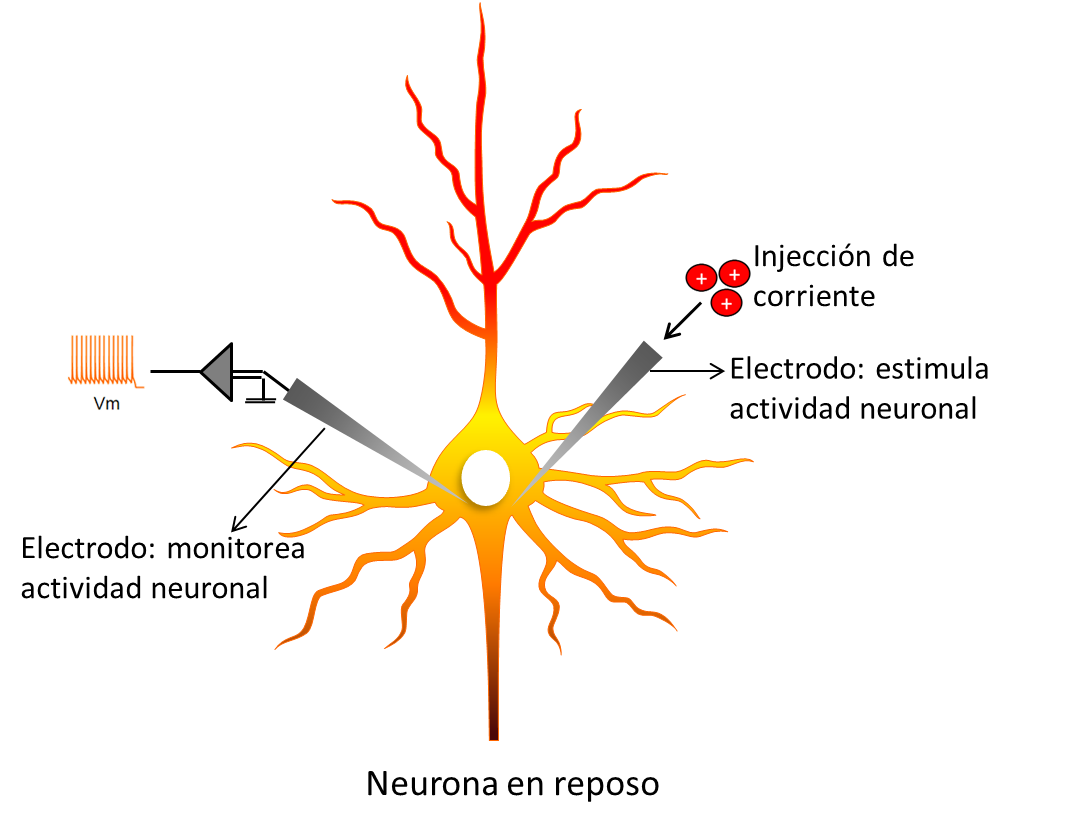


Ahora, si otro electrodo de vidrio se inserta cerca del primer electrodo pero ahora se le inyecta corriente positiva al segundo electrodo el potencial membranal se va a despolarizar (Fig 7). Cuando el potencial membranal llega a -50 mV un potencial de acción se generara. El “threshold” para la generación de un potencial de acción es -50 mV y esto significa que el potencial de la membrana debe ser despolarizado hasta -50 mV para poder generar un potencial de acción. Cualquier despolarización que no llegue a -50 mV fallara en iniciar esta señal eléctrica. La fuerza/magnitud del estímulo despolarizante determinará la frecuencia del potencial de acción.

Principios iónicos del potencial de acción

Cuando el estímulo correcto despolariza el potencial membranal hasta -50 mV, los canales de Na^+^ sensitivos a voltajes abren y Na^+^ entra a la neurona rápidamente. La entrada de Na^+^ causa aún más despolarización y produce el “rising phase” del potencial de acción (Fig 8 (1)). Los iones de Na^+^ siguen entrando (2) a la neurona hasta que equilibrio es alcanzado (lo cual ocurre aproximadamente a 40 mV) esto forma el “overshoot” del potencial de acción (3). Rápido luego que el canal de Na^+^  abre se inactiva y la entrada de más iones de Na^+^ es evitada.

**Figura 7. Ejemplo de una medida electrofisiológica.**

**Figura 8. Generación del potencial de acción por estimulación con corriente.**

Durante el “rising phase” del potencial de acción el potencial membranal requerido para abrir los canal sensitivos a voltaje de K^+^ se alcanza (0 mV), pero este canal no abre hasta después del “overshoot” del potencial de acción generando un atraso (o “delay”) en su apertura. Una vez abierto, K^+^ fluye hacia el espacio extracelular causando la repolarización del potencial membranal y el “falling phase” del potencial de acción (4). Durante el “undershoot” (5) del potencial de acción el potencial membranal está mucho más negativo que el potencial membranal de reposo y esto es causado porque la membrana es mucho más permeable a K^+^ en este momento que cuando está en reposo. Por lo tanto, mucho más K^+^ está fluyendo hacia fuera de la neurona y menos cargas positivas están dentro de la neurona. Esta hiperpolarizacion del potencial membranal cierra los canales de K^+^ sensitivos a voltaje y desactiva los canales de Na^+^ para que estos puedan ser cerrados.


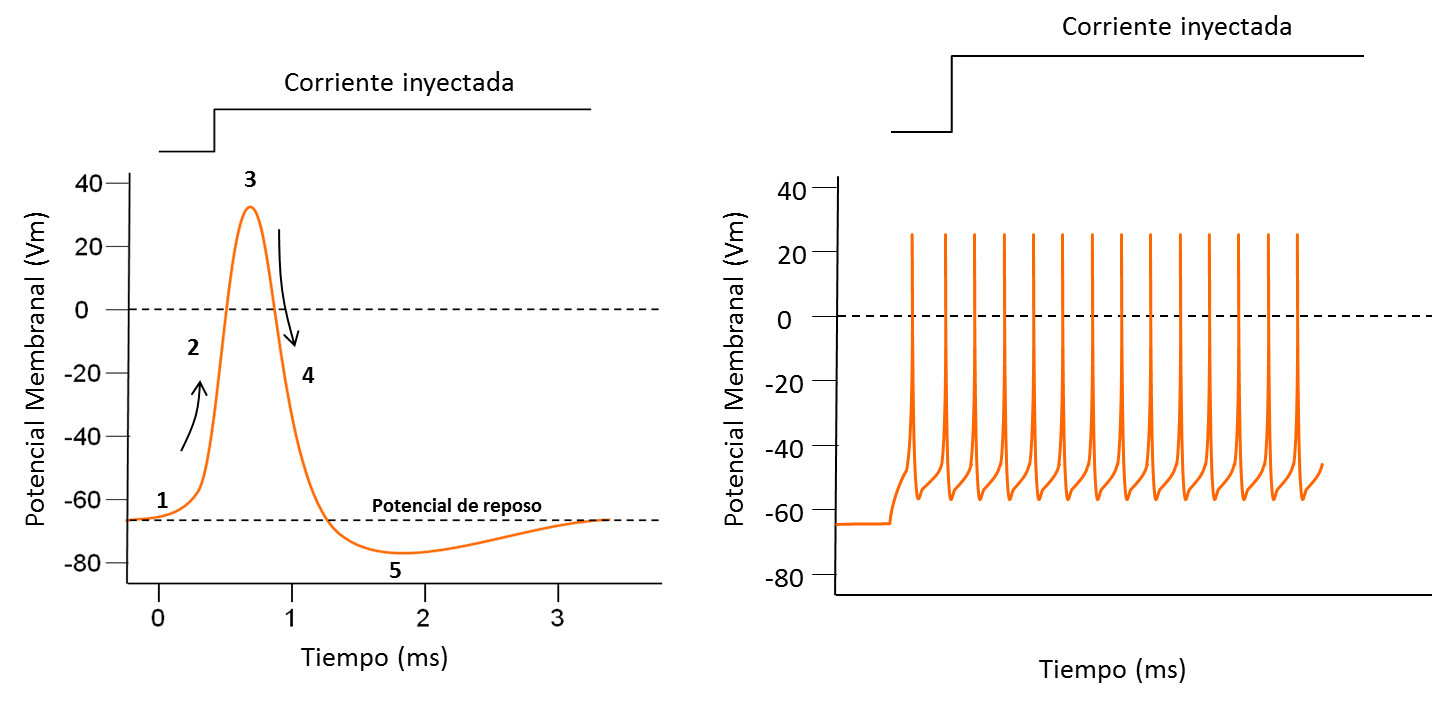


Propagación del potencial de acción

La velocidad de propagación del potencial de acción es factor limitante en la transferencia de información en el sistema nervioso. La propagación del potencial de acción requiere dos tipos de corriente: activa y pasiva. Despolarización del potencial membranal abre los canales de Na^+^ permitiendo la entrada de este ion al axón de la neurona (corriente activa) y generando un potencial de acción. Esta corriente de iones de Na^+^ ahora tiene que pasivamente trasladarse hacia donde se encuentra el siguiente canal de Na^+^ sensitivo a voltaje, y despolarizar el potencial membranal en esa localidad para producir la apertura de este canal de Na^+^, y generar otro potencial de acción en este punto. Mientras más se traslade la corriente de Na^+^ pasivamente más rápido será la propagación del potencial de acción a través del axón. Cuán lejos se traslade la corriente pasivamente está limitado por la cantidad de corriente que se pierda a través de la membrana. Para resolver este problema, las neuronas desarrollaron una estrategia basada en insular el axón con múltiples capas de membranas de células gliales para de esta manera disminuir la cantidad de corriente que se pierde a través de la membrana. Las múltiples capas de membranas se le conocen como la capa de mielina (Fig 9). Axones que no poseen la capa de mielina conducen potenciales de acción a una velocidad de 0.5 a 10 m/s, en cambio axones que si poseen la capa de mielina pueden conducir potenciales de acción tan rápido como 150 m/s.


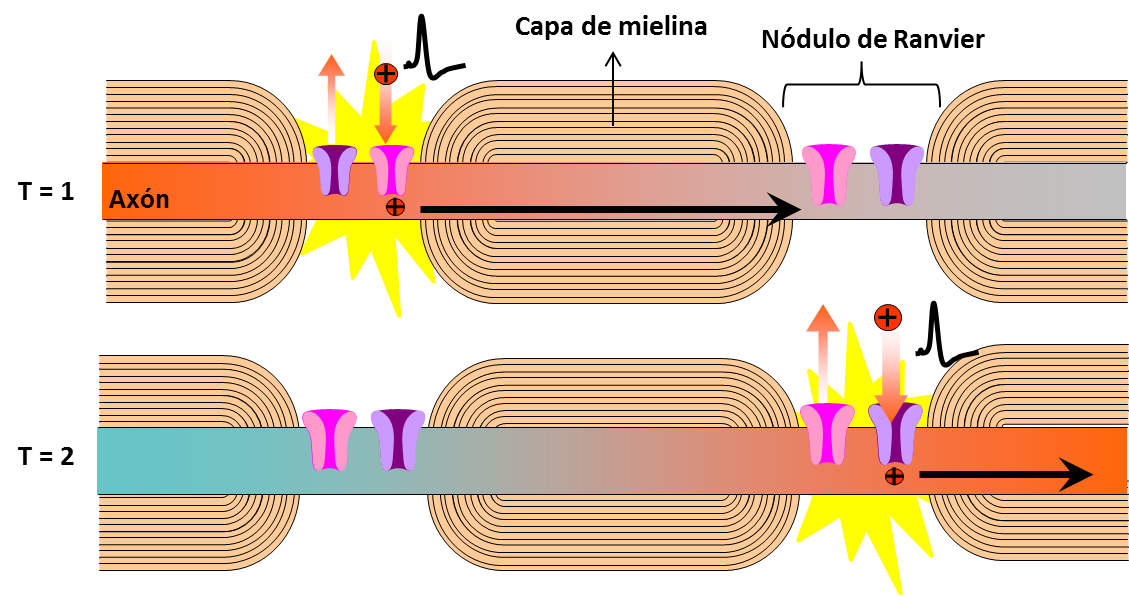


**Figura 9. Propagación de un potencial de acción en un axón múltiples capas de mielina.**

En axones que contienen la capa de mielina, el potencial de acción ocurre en lugares específicos llamados *nódulos de Ranvier*. En estos nódulos no hay mielina y estos contienen una alta concentración de canales de Na^+^ sensitivos a voltaje. La corriente pasiva que se genera luego que ocurre un potencial de acción se traslada hasta el siguiente nódulo de Ranvier en donde se genera otro potencial de acción. El ciclo se repite hasta que el potencial de acción llegue al terminal del axón. Este tipo de propagación recibe el nombre de conducción saltatoria porque el potencial de acción está brincando de nódulo a nódulo. En axones que no contienen la capa de mielina el potencial de acción ocurre en múltiples localidades y esto en el contexto de neuronas es un proceso que demanda mucho tiempo (Fig 10).


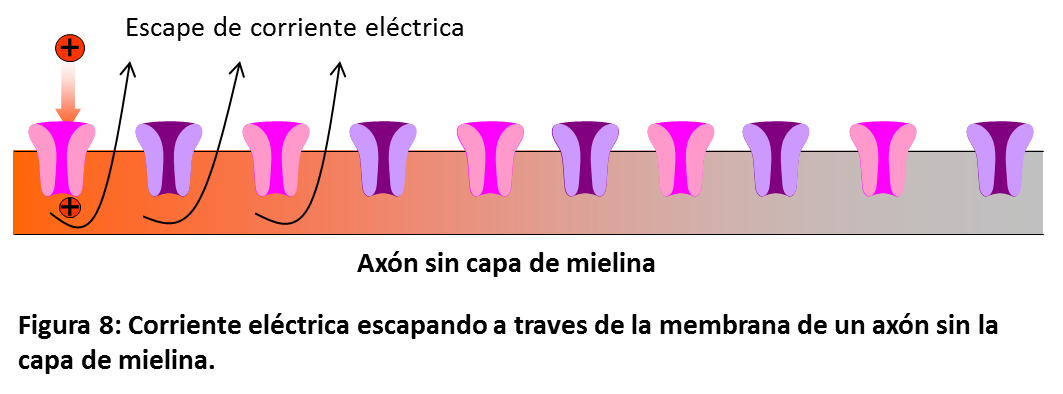


**Figura 10. Corriente eléctrica escapando a través de la membrana de un axón sin la capa de mielina.**

**Características universales de La sinapsis química**

En una sinapsis química, cuando el potencial de acción llega al terminal sináptico o nervioso este inducirá la liberación o exocitosis del neurotransmisor.

Características (Fig 11):

1. Toda sinapsis tiene un componente pre-sináptico y un componente post-sináptico. El componente pre-sináptico es siempre un terminal nervioso (terminal sináptico o terminal del axón). El componente post-sináptico puede ser otra neurona (dendritas, soma, terminal de axón).
2. Los componentes pre- y post-sinápticos están separados por un espacio llamado “synaptic cleft”. Este espacio es de aproximadamente 20 nm y contiene una matriz de proteínas que funcionan para mantener la sinapsis en buen estado.
3. El componente pre-sináptico contiene las vesículas sinápticas que a su vez almacenan los neurotransmisores. Existen dos tipos de vesículas sinápticas: pequeñas con centro claro (50 nm diámetro) y grandes con centro
4. oscuro/denso (100 nm diámetro). Estas almacenan neurotransmisores pequeños como acetilcolina y neuropeptidos, respectivamente.
5. Ambos componentes acumulan proteínas y esto recibe el nombre de diferenciaciones membranales. En este contexto existen dos tipos de sinapsis: Asimétricas (acumulación de proteínas es diferente en ambos componentes) y simétricas (acumulación de proteínas es la misma en ambos componentes). Frecuentemente, sinapsis simétricas son inhibitorias y sinapsis asimétricas son excitatorias.
6. Zonas activas son acumulaciones de proteínas en el componente pre-sináptico y contiene todas las proteínas que son necesarias para la exocitosis del neurotransmisor.
7. La densidad post-sináptica son acumulaciones de proteínas en el componente post-sináptico. Esta densidad contiene los receptores para neurotransmisores los cuales convierten la señal química en una señal eléctrica en la célula post-sináptica.
8. La respuesta del componente post-sináptico depende del tipo de neurotransmisor que se libere y del tipo de receptor que el neurotransmisor active.


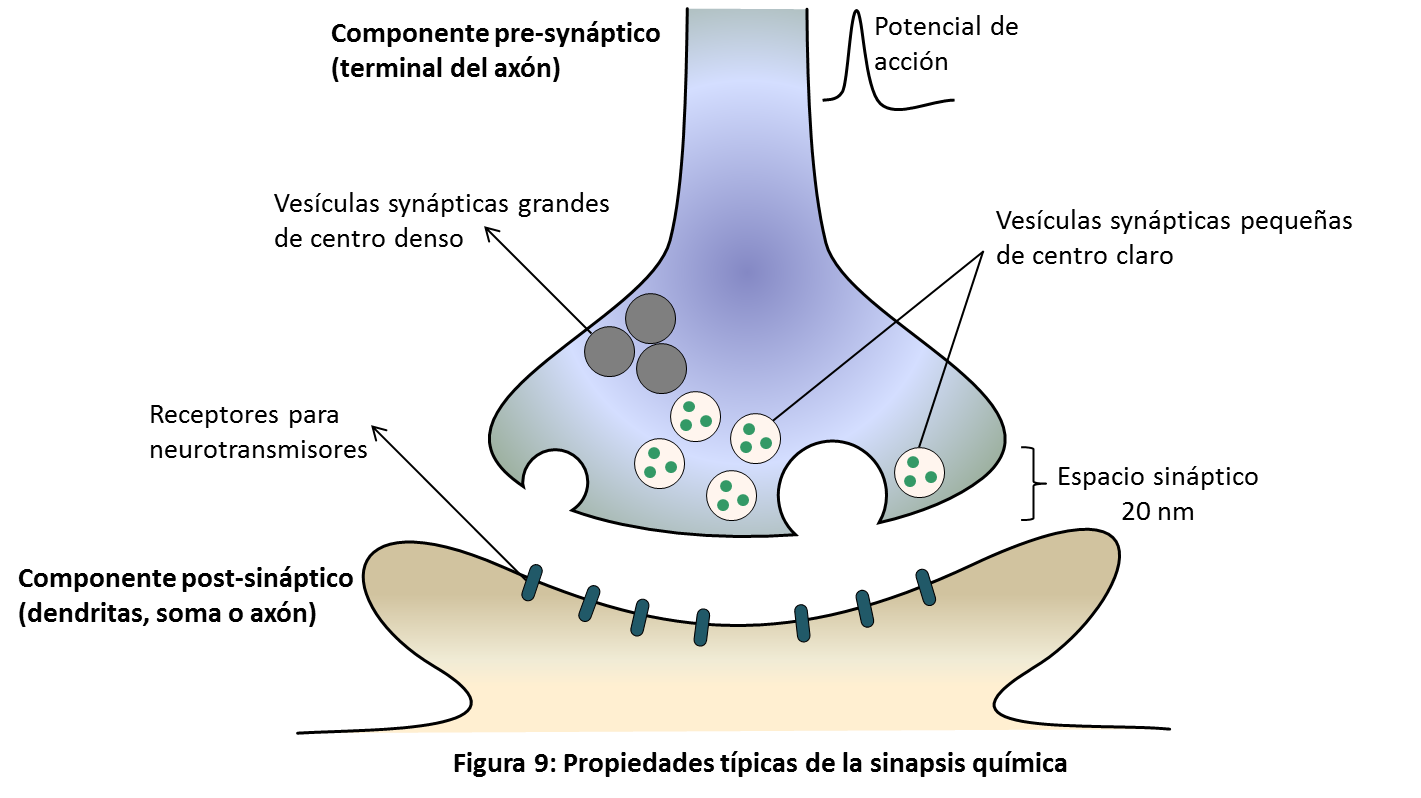


**Figura 11. Propiedades típicas de la sinapsis química.**

**Eventos importantes durante una sinapsis química (Fig 12)**

1. El potencial de acción invade el terminal nervioso de la neurona pre-sináptica.
2. Esto cause un cambio despolarizante en el potencial membranal que causa la apertura de canales de Ca^+2^ sensitivos a voltaje.
3. Ca^+2^ se mueve hacia dentro del terminal nervioso siguiendo su gradiente de concentración.
4. Las vesículas sinápticas que contienen el neurotransmisor son capaces de sentir Ca^+2^ dentro del terminal nervioso y esto es una señal para liberar el neurotransmisor al “syanptic cleft”. La membrana de la vesícula sináptica se funde con la membrana del terminal nervioso en un proceso llamado exocitosis, liberando el contenido dentro de la vesícula sináptica (neurotransmisores).
5. Los neurotransmisores ahora viajan a través del “synaptic cleft” hasta llegar a sus respectivos receptores en la célula post-sináptica.
6. La unión de neurotransmisor-receptor va a causar un cambio en el movimiento de iones en la célula post-sináptica.
7. El potencial membranal de la célula post-sináptica puede ser despolarizado o hiperpolarizado aumentando o disminuyendo la probabilidad de generar un potencial de acción, respectivamente.
8. La acción del neurotransmisor debe ser terminada. El neurotransmisor puede ser:

- Degradado en el “synaptic cleft”
- Re-ciclado por células gliales o por terminal sináptico
- Pasivamente alejado de la sinapsis

1. Reciclaje de la membrana de las vesículas sinápticas.


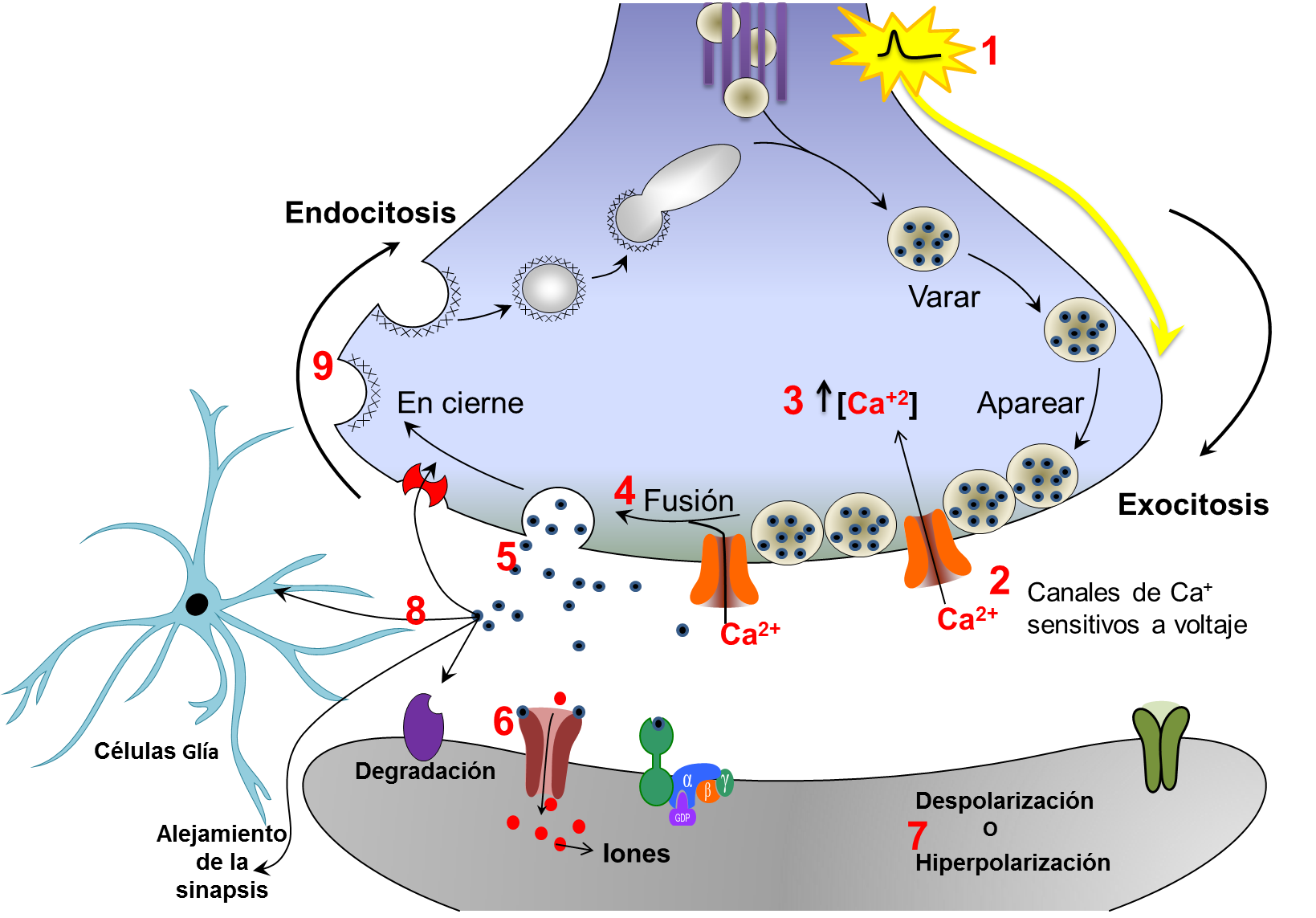


**Figura 12. Resumen de todos los pasos envueltos en la sinapsis química**

**Figura 12. Resumen de los eventos importantes de la sinapsis química .**

**CÓMO TRABAJA TU SISTEMA NERVIOSO?**

**Sistema Sensorial**

**EXPERIMENTO #1: Jellybean Taste Test**

 Usamos nuestro **sistema sensorial** para navegar e interactuar con el mundo que nos rodea. El sistema sensorial humano incluye visión, audición, tacto, gusto, y olfato. El sistema sensorial puede recibir señales de una gran variedad de estímulos. Esta información sensorial es convertida en una señal eléctrica que es retransmitida al cerebro. La mayor parte de la información sensorial es filtrada en un área del cerebro llamada el **tálamo**. La información es procesada aún más en áreas especializadas del cerebro que procesan diferentes tipos de información sensorial (Fig 13). Sin embargo, estas áreas no están aisladas; existen conexiones entre estas áreas del cerebro que te permiten obtener una percepción sensorial completa del ambiente.

En este experimento vamos a explorar cómo los sistemas sensoriales individuales trabajan en sincronía. Los procesos de olfato y gusto surgen cuando moléculas químicas se separan de sustancias y flotan hacia la nariz o llegan a nuestra boca donde se unen a células sensoriales y estimulan células nerviosas. Estas células sensoriales transmiten mensajes a centros en el cerebro que nos hacen percibir olores y/o sabores. Aunque los sistemas de neuronas (células sensoriales, vías de nervios, y centros primarios en el cerebro) son distintos, las sensaciones de sabor y olores usualmente trabajan en conjunto.

**Figura 13. El Sistema Sensorial.**

**MATERIALES:**

- Jellybeans

**MÉTODO:**

Encuentra un/a compañero/a. Deberán turnarse para ser experimentador/a y sujeto. El experimentador le dará un jelly bean al sujeto bajo las tres condiciones especificadas abajo. El sujeto tratará de adivinar el sabor del jelly bean bajo cada condición. El experimentador apuntará dos observaciones: (1) de qué color (sabor) es el jelly bean antes de dárselo al sujeto, y (2) el sabor que el sujeto reporta bajo cada condición. Las tres condiciones son:

(1) Con los ojos y nariz cerrados

(2) Con los ojos cerrados, pero nariz abierta

(3) Con los ojos y nariz abiertos

**HIPÓTESIS:** [Considera las siguientes preguntas: ¿Bajo que condiciones tu crees que tu compañer@ va a poder determinar el sabor del Jellybean correcto? ¿Por qué?]

**OBSERVACIONES:**

| **SUJETO** | **SABOR BAJO CONDICIÓN #1** | **SABOR BAJO CONDICIÓN #2** | **SABOR BAJO CONDICIÓN #3** |
| --- | --- | --- | --- |
|  |  |  |  |
|  |  |  |  |

**CONCLUSIÓN:**

**EXPERIMENTO #2: Discriminación de Dos Puntos**

**A**


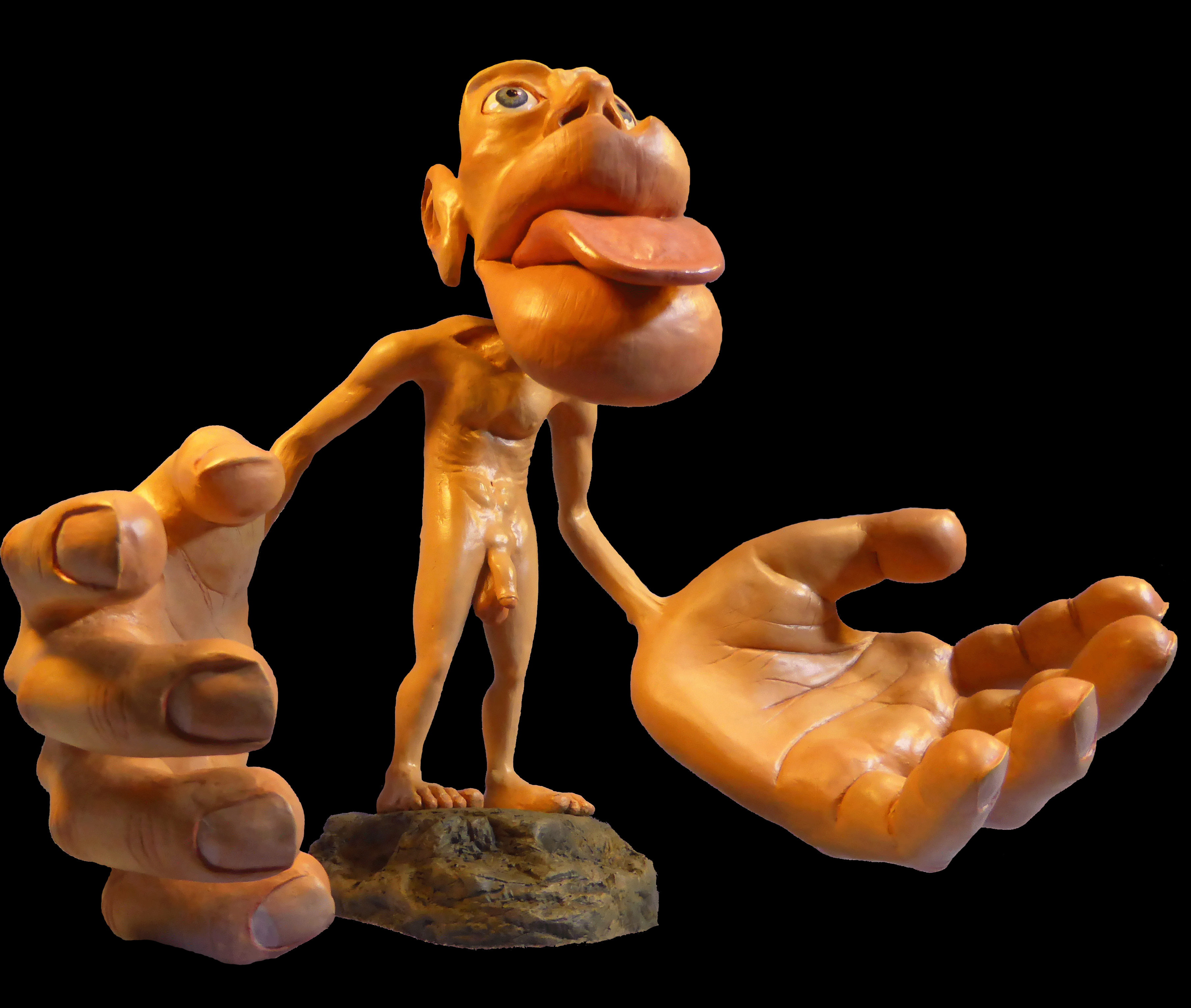
 La información que obtenemos de nuestra piel nos permite identificar varios tipos de sensaciones, tales como tacto, vibraciones, presión, dolor, y temperatura. ¿Qué nos permite distinguir entre estos tipos de sensaciones? Primero, la piel humana contiene diferentes tipos de receptores sensoriales que responden preferentemente a varios estímulos mecánicos, termales, o químicos (Fig 14A). Segundo, las células sensoriales transmiten esta información al cerebro y médula espinal a través de nervios que convierten las energías mecánicas, termales, y químicas a señales eléctricas. La manera que interpretamos sensaciones depende no solo de las propiedades de los receptores sensoriales y neuronas, sino también de pasadas experiencias almacenadas en nuestro cerebro.

La información sensorial que recibe el cerebro se procesa en áreas específicas. La información de cada receptor en la piel se transporta a lo largo de una vía, formada de muchos axones de neuronas, a una parte del cerebro llamada la corteza somatosensorial. Esta área está empacada con neuronas que reciben la información de la piel en diferentes partes del cuerpo. La información sensorial es organizada en el cerebro de manera topográfica. Esto significa que las neuronas que reciben la información de las neuronas sensoriales en el dedo pulgar estarán localizadas en el cerebro en gran proximidad a las que reciben la información del dedo índice. De esta manera, un mapa sensorial de la superficie del cuerpo es creado en una sección de la superficie del cerebro. *El homúnculo sensorial* (Fig 14B) es una imagen que se utiliza para describir un modelo de un humano que refleja el espacio relativo que diferentes partes del cuerpo ocupan en la corteza somatosensorial. Por ejemplo, la punta de los dedos contiene 100 veces más receptores por centímetro cuadrado que la piel en tu espalda, y por tanto más neuronas del cerebro están dedicadas a recibir sensaciones de la punta de tus dedos. Consecuentemente, el área de la corteza somatosensorial que recibe información de la punta de tus dedos es mucho más grande comparada con el área que recibe información de la piel en tu espalda. En este experimento, vamos a examinar el sentido táctil de tu piel, que nos permite distinguir entre diferentes tipos de estímulos en la superficie de tu cuerpo.

**Figura 14. A) Representación de diferentes tipos de receptores y los tipos de sensaciones que detectan. B) El homúnculo sensorial** (imagen por Mpj29 compartida bajo la licencia Creative Commons Attribution-Share Alike 4.0 International).

**B**

**MATERIALES:**

- Compás

**MÉTODO:**

Consigue un compañero/a. Deberán turnarse para ser experimentador/a y sujeto. El sujeto cerrará sus ojos, mientras el experimentador usará el compás para tocar la piel del sujeto ligeramente. El experimentador le preguntará al sujeto si siente uno o dos puntos. El experimentador continuará ajustando el compás hasta que el sujeto reporte que solamente siente un punto. El experimentador apuntará cuál es la distancia más pequeña a la cual el sujeto pudo detectar los dos puntos.

**HIPÓTESIS:**

**OBSERVACIONES:**

| **ÁREA DE LA PIEL EXAMINADA** | **MÍNIMA DISTANCIA DE DISCRIMINACIÓN DE DOS PUNTOS**  En milímetros (mm) |
| --- | --- |
| Frente |  |
| Mejilla |  |
| Antebrazo |  |
| Palma de la mano |  |
| Dedo índice |  |
| Pantorrilla/pierna |  |
| Dedo pulgar |  |

**CONCLUSIONES:**

**PREGUNTAS:**

1. ¿Qué crees que determina tu habilidad de distinguir entre uno y dos puntos?

2. ¿Cuál es la relación entre el número de receptores en la piel y el tamaño de la corteza somato sensorial que está encargada de la interpretación de la sensación de esa parte del cuerpo?

3. ¿Cuánta área de la corteza somato sensorial está dedicada a recibir información sensorial de cada una de las regiones que investigaste? (circula una respuesta):

Dedo pulgar: Grande Mediana Pequeña

Mejilla: Grande Mediana Pequeña

Frente: Grande Mediana Pequeña

Pantorrila/pierna: Grande Mediana Pequeña

Antebrazo: Grande Mediana Pequeña

5. ¿Qué podrías hacer para cambiar la percepción de tacto en este experimento? ¿Harías este cambio a nivel de la piel o del cerebro?

**Sistema Motor**

**EXPERIMENTO #3: Reflejo Rotuliano**

El **Sistema Motor** es la parte del Sistema Nervioso que es responsable por la contracción y coordinación de los músculos. La unión neuromuscular es un área donde el terminal de un axón de una neurona motora conecta con una fibra muscular. Cuando reciben una señal del cerebro, las neuronas motoras liberan neurotransmisores a uniones neuromusculares para causar la contracción de fibras musculares. Mientras más fibras musculares se activan, más fuerte es la magnitud de la contracción.

Hay diferentes tipos de músculos (esquelético, cardiaco, músculo liso) con diferentes funciones en el cuerpo. Por tanto, el Sistema Motor es responsable por diferentes tareas tales como movimientos mecánicos, contracción del músculo cardiaco, contracción del músculo intestinal, entre otras.

**Figura 15. Reflejo rotuliano o patelar.**

El siguiente ejercicio demostrará cómo las contracciones de reflejo muscular ocurren. Los reflejos son movimientos musculares involuntarios y casi instantáneos que tienen un rol importante en la protección del cuerpo humano. Su rápido inicio de acción se debe a que no requieren del análisis e instrucción del cerebro para que ocurran. El reflejo rotuliano o patelar es un tipo de reflejo muscular (Fig 15). Te enseñará cómo el sistema sensorial y motor trabajan juntos en cuestión de milisegundos!

**MATERIALES:**

- Tu mano
- Una persona sentada con sus piernas cruzadas

**MÉTODO:**

Haz que tu compañer@ se sienta con sus piernas cruzadas, de manera que su pierna pueda oscilar libremente. Golpea a tu compañer@ justo debajo de su rodilla con el lado de tu mano. Si lo haces correctamente, la pierna de tu compañer@ dará una patada casi inmediatamente.

**OBSERVACIONES:**

**CONCLUSIONES:**

**PREGUNTA:**

1. ¿Por qué los doctores verifican la función del reflejo rotuliano/patelar?

**EXPERIMENTO #4: Tiempo de Reacción:**

La comunicación eficaz entre tu sistema sensorial y sistema motor te permite protegerte de situaciones perjudiciales. Cuando alguien te tira un bola, tu primer instinto es el de subir tus manos para proteger tu cuerpo. Si tocas algo bien caliente no tienes que pensar dos veces para retirar tu mano. Estos y otros reflejos musculares dependen de la comunicación rápida entre las neuronas del sistema sensorial y las neuronas del sistema muscular. La velocidad de esta comunicación depende en parte de la distancia que la señal tiene que recorrer.

En este experimento vamos a estudiar la coordinación entre el sistema sensorial y el sistema motor. Vas a medir el tiempo de reacción a un estímulo en particular, en este caso la caída de una regla. Cuando hagas este ejercicio ten en cuenta lo importante que es el tener respuestas rápidas a los estímulos que enfrentas cada día.

**MATERIALES:**

- Regla

**METODO:**

Haz que tu compañer@ sostenga una regla. Mantén tus dedos índice y pulgar abiertos en la parte de debajo de la regla (cerca de la marca de 0 pulgadas). Tu compañer@ dejara caer la regla al azar 3 veces y tú tienes que agarrar la regla lo más pronto posible (Nota: Tu compañer@ no debe decirte cuando va a dejar caer la regla). Apunta la marca en pulgadas donde agarraste la regla cada vez. Convierte las pulgadas a segundos con la tabla.

**HIPÓTESIS:**

**OBSERVACIONES:**

| **TIEMPO DE REACCIÓN (PULGADAS)** | | |
| --- | --- | --- |
| **PRUEBA #1** | **PRUEBA #2** | **PRUEBA #3** |

| Distancia (pulgadas) | Tiempo (segundos) |
| --- | --- |
| **2** | 0.10 (100 ms) |
| **4** | 0.14 (140 ms) |
| **6** | 0.17 (170 ms) |
| **8** | 0.20 (200 ms) |
| **10** | 0.23 (230 ms) |
| **12** | 0.25 (250 ms) |
| **17** | 0.30 (300 ms) |
| **24** | 0.35 (350 ms) |
| **31** | 0.40 (400 ms) |
| **39** | 0.45 (450 ms) |
| **48** | 0.50 (500 ms) |
| **69** | 0.60 (600 ms) |

| **TIEMPO DE REACCIÓN (SEGUNDOS)** | | | |
| --- | --- | --- | --- |
| **PRUEBA #1** | **PRUEBA #2** | **PRUEBA #3** | **PROMEDIO** |
|  |  |  |  |

**PREGUNTAS:**

1. Cuales otros factores y/o condiciones crees que podrían afectar el tiempo de reacción?

**CONCLUSIÓN:**

**Sistema Nervioso Autonómico**

**EXPERIMENTO #5: Presión Sanguínea**

El **Sistema Nervioso Autonómico** (SNA) es la división del Sistema Nervioso que es responsable por el control del movimiento de tus intestinos, ritmo cardiaco, y glándulas. Existen dos divisiones del SNA: el **sistema nervioso simpático** y **parasimpático** (Fig 16). Para la mayoría de las funciones corporales, estas dos divisiones trabajan en direcciones opuestas para controlar exactamente la función de tus órganos. Por ejemplo, el sistema nervioso simpático aumenta el ritmo cardiaco, mientras que el sistema nervioso parasimpático disminuye el ritmo cardiaco.

En este ejercicio vamos a explorar como factores externos, como drogas o ejercicios, pueden afectar la función de las dos divisiones del SNA; usaremos medidas de presión sanguínea para determinar esto. Has aprendido en las pasadas lecturas como desarrollar y probar hipótesis. ¡Ahora tienes la oportunidad de formular y probar tu hipótesis! ¿Crees que la cafeína activa el sistema nervioso simpático o parasimpático? ¿Cómo lo comprobarías? ¿Y que tal el ejercicio? ¿Estrés? ¿Algún otro factor?

**Figura 16. Sistema Nervioso Autonómico.**

**MATERIALES:**

- Manga para medir presión sanguínea
- Bebidas con cafeína
- Cronógrafo
- Escaleras
- Sé creativo/a…

**MÉTODO:**

Usando los materiales que tienes disponible o cualquier otro material que se te ocurra, formula una pregunta de investigación que puedas probar. También formula y prueba una hipótesis acerca de cómo un factor externo (drogas (cafeína), ejercicio, estrés) afecta el balance entre las divisiones simpáticas y parasimpáticas del SNA.

**HIPÓTESIS:**

**OBSERVACIONES:**

**CONCLUSIONES:**

**Referencias**

1. Bear MF, Connors BW, Paradiso MA. (2006). Neuroscience Exploring the Brain. Third edition, Lippincott Williams and Wilkins.

2. Kandel ER, Schwartz JH, and Jessell TM. (2000). Principles of Neural Science. Fourth edition, New York: McGraw Hill Health Professions Division.

3. Dana Foundation. www.dana.org

4. Brain Facts. www.brainfacts.org

5. Neuroscience for Kids. www.faculty.washington.edu/chudler/neurok.html
