# Supplementary material for: Bridge to neuroscience workshop: An effective educational tool to introduce principles of neuroscience to Hispanics students
Source: PLoS One. 2019 Dec 12;14(12):e0225116. doi: 10.1371/journal.pone.0225116 (PMC6907774; doi:10.1371/journal.pone.0225116)
Supplement: S6 File — (DOC) [file pone.0225116.s007.doc]

Date:______________________ (Pre/Post) Evaluation #_____

Completion of this test will allow us to evaluate your understanding about important concepts of neuroscience before and after participation in the Bridge to Neuroscience Workshop. Be sure to answer all the questions to the best of your knowledge.

1. Choose the option that better describe the main components of the nervous system:

a. Brain and spinal cord

b. Sympathetic and parasympathetic nervous system

c. Brain and peripheral nervous system

d. Brain, spinal cord, sympathetic division, parasympathetic division, and somatic division

2. Neurons are a unique type of cell because they have specialized structures that allow them to effectively communicate between each other. Draw a picture of a neuron and include the structures (four main structures) they use for receiving and sending information.

3. Two ways in which neurons are different from other types of cells are:

a. Generation of electrical signals and ability to communicate by releasing neurotransmitters (chemical communication)

b. Neurons have a cell soma and cellular organelles

c. Neurons have a plasma membrane and generate electrical signals

d. Release neurotransmitters and use chemical synaptic transmission

4. Two ways in which neurons communicate with each other are:

a. Chemical transmission and neurotransmitters

b. Action potential and electrical signals

c. Chemical transmission (neurotransmitter) and electrical signals (action potential)

d. None of the above

5. Choose the option that better describe the parts of the nervous system that allow us to interact with the environment.

a. Sensory systems: primary somatosensory area, auditory cortex, visual cortex, gustatory cortex, olfactory cortex, and all the receptors that sense the different sensory stimuli.

b. The brain by itself is capable of doing this

c. The nervous system does not play a role

d. Spinal cord

6. The primary somatosensory cortex is the area of the brain processing information about the sense of touch. Choose the option that better describe the organization of this area.

a. There is no organization

b. There is a map of the body surface in this area and sensitivity of different body parts is represented by having a greater amount of brain tissue processing sensory information (Homunculus)

c. It is organized by the shape of the neurons

d. None of the above

7. Resting membrane potential in neurons is around -70 mV and is mainly due to the flow of ______ towards the __________ of the cell.

a. Na+ and inside

b. K+ and outside

c. Na+ and outside

d. Cl- and inside

8. There are two main ions responsible of generating action potentials in neurons. Those are:

a. Cl- and Na+

b. Na+ and Ca2+

c. Na+ and K+

d. K+ and Ca2+

9. Imagine you are swimming in the ocean and you notice a shark approaching. The part of the autonomic nervous system that is activated is __________. Therefore, the function of organs such as the pupils, heart and lungs, will be _________.

a. Sympathetic nervous system and enhanced

b. Parasympathetic nervous system and enhanced

c. Sympathetic nervous system and inhibited

d. Parasympathetic nervous system and inhibited

10. What is the name of the area where a neuron comes in contact with a muscle?

a. Muscle neuron target

b. Neuromuscular junction

c. Post synaptic cell

d. Node of Ranvier

11. The sulci and gyri are found on the surface of the cerebral cortex and their main function is to:

1. No significant function in known
2. Increase the cerebral surface area
3. Make the brain look prettier
4. Enhance communication between neurons

TWO STARS AND A WISH

Please describe two things that you enjoyed today and one thing that you would change.

**WISH**

TWO STARS AND A WISH

Please describe two things that you enjoyed today and one thing that you would change.

**WISH**

TWO STARS AND A WISH

Please describe two things that you enjoyed today and one thing that you would change.

**WISH**
